# High-spatiotemporal-resolution distributed Brillouin sensing with transient acoustic wave

**DOI:** 10.1038/s41377-025-01848-4

**Published:** 2025-06-03

**Authors:** Yin Zhou, Yuan Cheng, Jia Ye, Zonglei Li, Haijun He, Wei Pan, Bin Luo, Lianshan Yan

**Affiliations:** 1 Center for Information Photonics & Communications, School of Information Science & Technology, Southwest Jiaotong University, Chengdu, 610031 Sichuan China; 2 Department of Automation, Tsinghua University, Beijing, 100084 China; 3 School of Artificial Intelligence, Shanghai Jiao Tong University, Shanghai, 200030 China; 4 Department of Electronic Engineering, Tsinghua University, Beijing, 100084 China

**Keywords:** Optical sensors, Fibre optics and optical communications

## Abstract

Real-time wide-area environment sensing is crucial for accessing open-world information streams from nature and human society. As a transformative technique distinct from electrical sensors, distributed optical fiber sensing especially for Brillouin scattering-based paradigm has shown superior bandwidth, power, and sensing range. Still, it suffers from insufficient resolution and timeliness to characterize remote dynamic events. Here we develop TABS—a transient acoustic wave-based Brillouin optical time domain analysis sensor, supporting long-range high-spatiotemporal-resolution distributed sensing. By designing a functionally synergistic sensor architecture, TABS elaborately leverages wideband and time-weighted energy transformation properties of a transient acousto-optic interaction to breaking through Brillouin-energy-utilization-efficiency bottleneck, enabling enhancements in overall sensing performance. In the experiment, TABS has achieved a 37-cm spatial resolution over a 50-km range with 1 to 2 orders of magnitude improvement in temporal resolution compared to prevailing Brillouin sensing approaches. For the first time, TABS is explored for state imaging of evacuated-tube maglev transportation system as an exemplary application, showcasing its feasibility and flexibility for potential open-world applications and large-scale intelligent perception.

## Abstract (web summary)

We develop TABS: a transient acoustic wave-based Brillouin optical time domain analysis sensor for high-spatiotemporal-resolution distributed sensing, exploring the state imaging of evacuated tube maglev transportation system.

## Graphical abstract

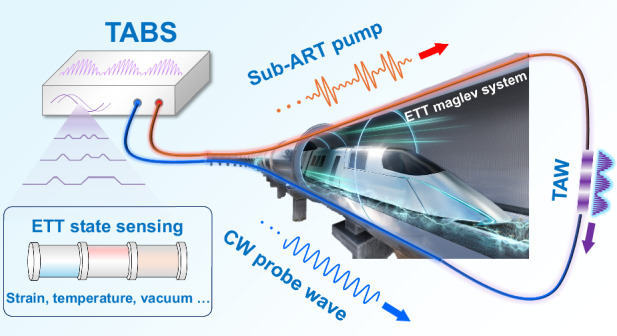

## Introduction

The rapid pace of infrastructure construction and frequent occurrence of natural disasters have led to urgent demands on real-time wide-area perception systems for probing flowing open-world information^1–9^. New-generation sensing networks may possess wide coverage area, high sensing density, and high information throughput to deal with diverse, dynamic, and unpredictable situations in the open-world applications. Traditional electrical sensor networks face certain limitations in bandwidth, power, and energy efficiency, which impede their development towards long range, high resolution, and high speed. Alternatively, owning to the low transmission attenuation of optical fiber, and high energy transformation of stimulated Brillouin scattering (SBS), the SBS-based distributed fiber-optic sensing (B-DFOS)^10–14^ has emerged as a revolutionary technique to reach long-range perception.

In nearly all B-DFOS paradigms, steady acoustic wave (SAW) has been extensively adopted as a fundamental operational mechanism for almost thirty years. This customary practice stems from the widely recognized conclusion that narrow Brillouin gain spectrum (BGS) generated by the SAW can support high spectral resolution, high sensing precision and wide measurement range^12–15^. Based on the SAW, a variety of methods have been presented to improve the sensing range^16–19^, spatial resolution^20–31^, or response speed^32–34^ independently, which drives individual performance of B-DFOS to gradually approach its upper bound. Nevertheless, achieving a B-DFOS with both long range and high spatiotemporal resolution remains a significant challenge. The trade-off between range and resolution stems from the inherent detrimental impacts of the SAW^17,18,35–38^ and inefficient utilization of the SBS energy, as analyzed in Supplementary Section 3. Consequently, the sensing scale, real-time capability, and information throughput are all constrained, hindering the achievement of open-world sensing tasks^1–9^.

While the SAW-based techniques have been extensively studied, transient acoustic wave (TAW) has received comparatively limited attention over the past three decades^39–44^. In refs. ^39–41^, the BGS broadening property under the combined effects of TAW and low-extinction-ratio pump pulse was investigated. In ref. ^42^, a transient Brillouin grating-based method was proposed to measure fiber birefringence distribution. In ref. ^43^, a transient differential pulse pair-based Brillouin fiber sensor was presented to decrease signal-to-noise ratio (SNR) reduction. In ref. ^44^, a rising edge demodulation algorithm in conjunction with the use of transient pump pulse is proposed to improve the spatial resolution significantly. Although these studies have contributed valuable insights into the potential of TAW for enhancing individual sensor performance, the BGS broadening and low spectral resolution induced by the TAW remain significant challenges, limiting its widespread adoption in the B-DFOS.

**Fig. 1:**
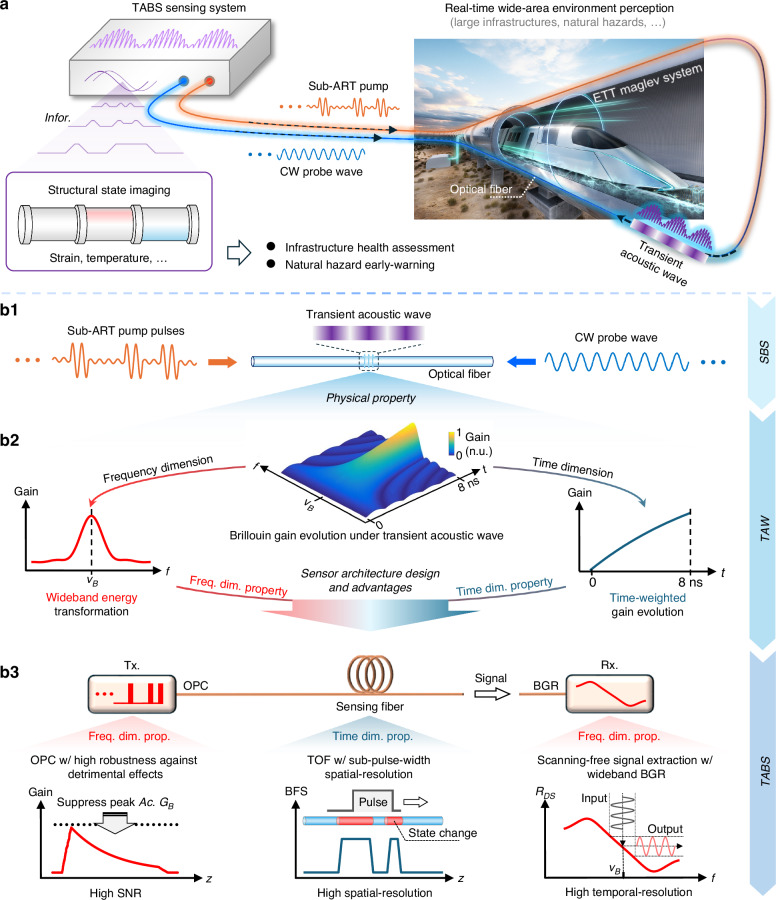
TABS: a transient acoustic wave (TAW)-based Brillouin optical time domain analysis sensor for high-spatiotemporal-resolution distributed sensing. **a** A conceptional illustration of TABS-based state imaging of large infrastructures and natural hazards. As an exemplary application, TABS is explored for state imaging of evacuated-tube transportation (ETT) maglev systems. Infor.: information stream. **b1**–**b3**, Working mechanism of TABS. **b1** The SBS interaction process. ART acoustic response time (~10 ns). The TAW occurs when the pump pulse width is shorter than the ART. **b2** Physical properties of TAW. n.u. normalized unit, *v*_*B*_ Brillouin frequency shift, Freq. dim. Frequency dimension. **b3** Sensor architecture of TABS. Freq. dim. prop. Frequency dimension property, Tx Transmitter, Rx Receiver, OPC optical pulse coding, *Ac. G*_*B*_ accumulated Brillouin gain, TOF time-of-flight location method, BGR Brillouin gain ratio, SNR signal-to-noise ratio. The TABS leverages wideband and time-weighted energy transformation properties of TAW by a synergistic architecture to enhance overall sensing performance

**Fig. 2:**
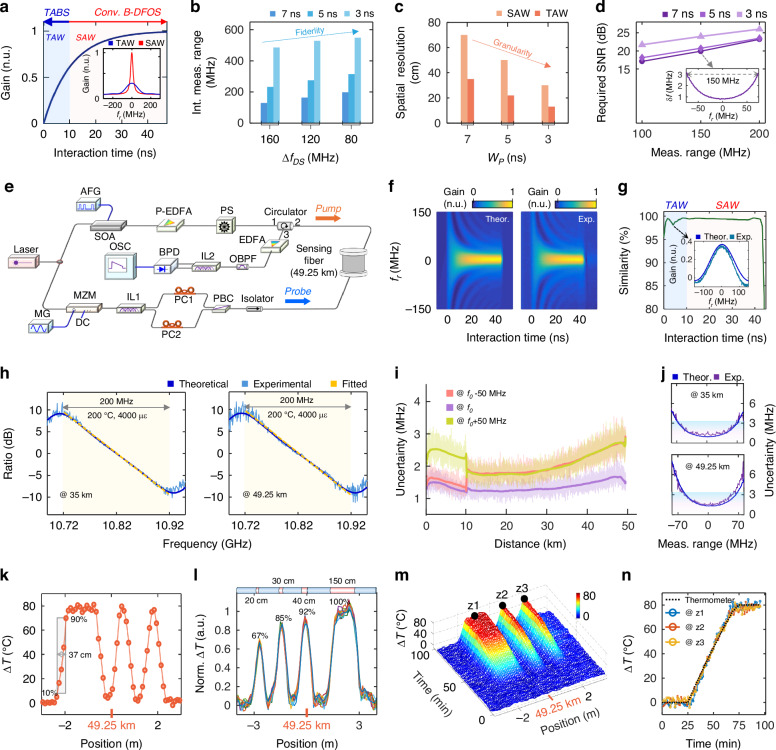
Sensing performance of TABS. **a** Theoretical acoustic wave temporal envelope (real part) at the BFS. The inset compares theoretical BGSs between SAW and TAW when energy accumulation time is 7 ns. Conv. conventional, n.u. normalized unit. Theoretical (**b**) intrinsic (int.) measurement ranges and (**c**) spatial resolutions across different effective pulse widths (*w*_*p*_) and Brillouin gain ratio frequency spacings (∆*f*_*DS*_). **d** Required SNR to achieve the target spatial resolutions and measurement ranges with an uncertainty (δ*f*) of less than 3 MHz. Here, ∆*f*_*DS*_ under different pulse widths are adjusted to maintain the same intrinsic linear region of 200 MHz. Meas. measurement, *f*_*r*_ relative frequency. **e** Experimental setup, the detailed introduction is listed in Materials and methods, and Supplementary Section 1. **f** Comparison of acoustic wave temporal envelopes (real part) between theory and experiment. *f*_*r*_ relative frequency, Theor. theoretical, Exp. experimental. **g** Similarity between theoretical and experimental acoustic wave temporal envelopes (real part), with the inset showing the acoustic wave spectrum at the lowest similarity point. **h** Measured Brillouin gain ratio spectra, where the light orange area indicates the linear region (i.e., measurement range). **i** Measurement uncertainty distribution along the fiber, which consists of two fiber sections with lengths of 10 km and 39.25 km, and BFSs of 10.8 GHz and 10.82 GHz, respectively. *T*_*0*_ intermediate temperature. **j** Measurement uncertainties at 35 km and 49.25 km under different frequency offsets. Measured static temperature variation (∆*T*) distributions for determining the (**k**) spatial resolution and (**l**) extreme sensing capability. In (**l**), the signals in 15-times measurements are overlapped together for verifying the repeatability in the extreme condition. **m** Measured sensor dynamic response. **n** Detailed BFS variation at z1, z2, and z3 positions shown in (**m**)

**Fig. 3:**
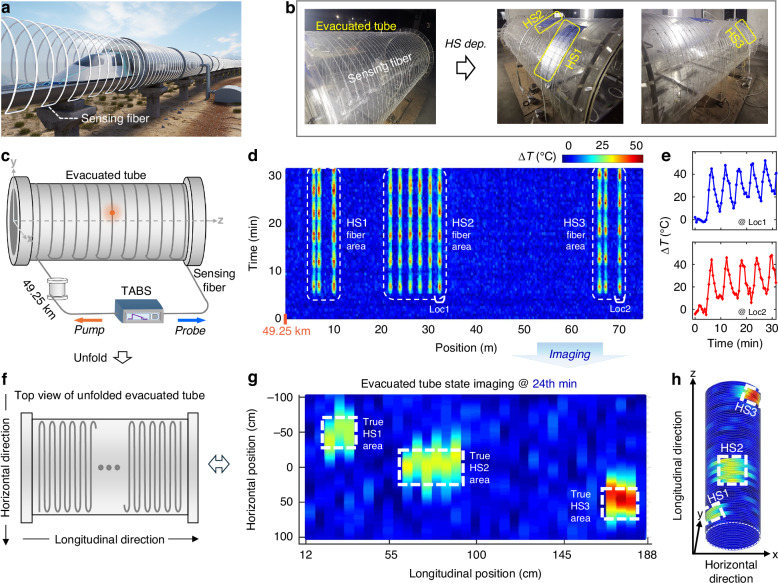
TABS for state imaging of evacuated tube body. **a** Conceptional illustration. The sensing fiber is installed on the evacuated tube body in a serpentine manner. The (**b**) digital photograph and (**c**) schematic diagram of field test. HS dep.: heat source deployment. Three heat sources (HS1, HS2, and HS3) with individually and periodically varied temperatures are deployed on the evacuated tube body. **d** Measured sensing information stream (BFS distribution at different times) in the vicinity of evacuated tube located at the end of the 49.25 km long sensing fiber. ∆*T* temperature variation. **e** Details of BFS variations at locations 1 and 2 (Loc1 and Loc2) as marked in (**d**). **f** Top view of unfolded evacuated tube. Imaged evacuated tube state at 24^th^ min in (**g**) 2D and (**h**) 3D views. The area in white dotted box indicates the true location of the preset heat source

**Fig. 4:**
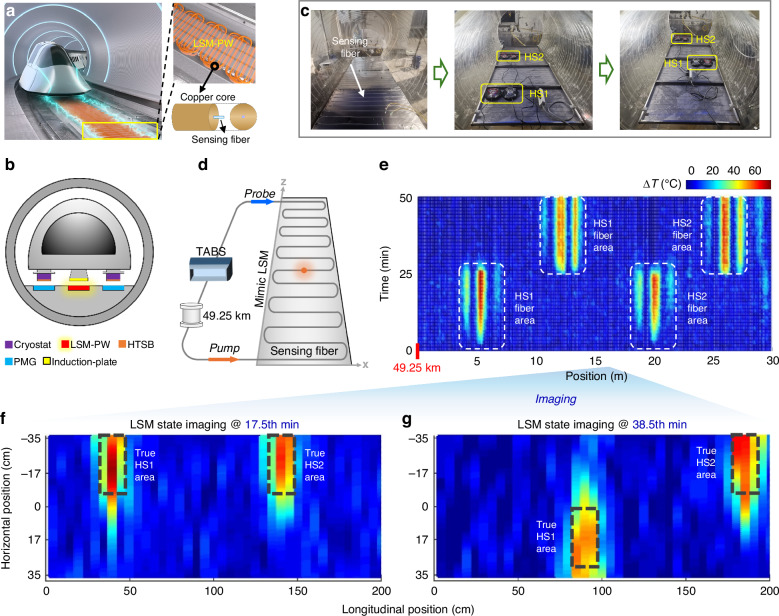
TABS for state imaging of linear synchronous motor. **a** Conceptional illustration. The right inset illustrates the primary windings (key device for vehicle traction) arranged in a serpentine manner. The sensing fiber could be embedded into the primary windings for precise temperature-rise imaging. **b** Sectional view of ETT maglev system. LSM-PW: linear synchronous motor’s primary windings, HTSB: high-temperature superconducting bulk, PMG: permanent magnetic guideway. The (**c**) digital photograph and (**d**) schematic diagram of field test. The sensing fiber cable is installed on the mimic LSM in a serpentine manner. Two heat sources (HS1 and HS2) are deployed on the backside of the side where the sensing fiber cables are installed. **e** Measured sensing information stream (BFS distributions at different times) in the vicinity of mimic ETT located at the end of the 50 km long sensing fiber. The area in white dotted box indicates the fiber BFS variation induced by the temperature rise. Imaged LSM state at **f** 17.5^th^ min and **g** 38.5^th^ min. The area in black dotted box indicates the true location of the preset heat source

**Fig. 5:**
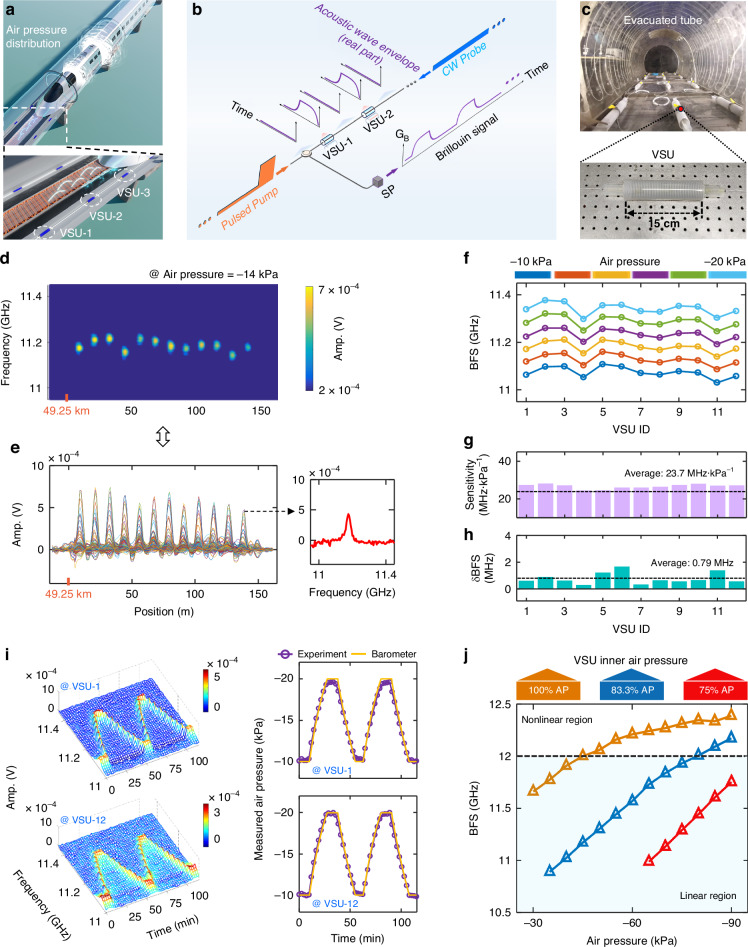
Vacuum sensitive array (VSA)-assisted Brillouin system for ETT vacuum distribution measurement. **a** Conceptional illustration. The sensing system configuration is the same as that of the TABS. The VSA, consisting of vacuum sensitive units (VSUs) in series, is deployed along the ETT line. **b** The working mechanism of distributed vacuum sensing. **c** Digital photograph of the VSA installation along the evacuated tube. Measured BGS distribution in **d** top and **e** side views when vacuum degree is 14% (air pressure = -14 kPa). **f** The BFS variations of the 12 VSUs. The **g** vacuum sensitivity and **h** measurement uncertainty of each VSU. **i** The response to dynamic vacuum variation of the first and last VSUs. **j** Vacuum measurement range under the VSU with different inner air pressure

Here we develop TABS, a **T**ransient **A**coustic**-**wave (TAW)-based **B**rillouin optical time domain analysis **S**ensor, to break through Brillouin-energy-utilization-efficiency bottleneck, facilitating long-range high-spatiotemporal-resolution distributed sensing. We theoretically and experimentally reveal that, by properly designing a functionally synergistic sensor architecture to elaborately leverage the unique physical properties of TAW, including wideband and time-weighted energy transformation, the SBS energy can be efficiently utilized to enhance both the sensing range and spatiotemporal resolution while minimizing detrimental effects (Fig. 1). Compared with prevailing B-DFOS techniques, TABS obtains a high spatial resolution (37 cm) over a long distance (50 km) with 1 to 2 orders of magnitude improvement in temporal resolution (21 s) (Fig. 2, Supplementary Tables S1 and S2 in Supplementary Section 3). The high-resolution wide-area perception capability enables a dense and precise characterization of long-range dynamic targets in both spatial and temporal scales. As a typical application, TABS is explored for state imaging of key components of evacuated tube transportation (ETT) maglev system, including evacuated tube body (Fig. 3), mimic linear synchronous motor (Fig. 4) and air pressure distribution (Fig. 5). The high resolution and fine versatility for sensing tasks in the ETT experiments showcase the feasibility and flexibility of TABS for potential open-world applications

Collectively, TABS stands to offer a new path for achieving overall sensing performance improvements. The high-spatiotemporal-resolution wide-area perception capability makes TABS a promising tool for observing the evolutionary mechanisms of geological activities, climate change, and large-scale infrastructure health, promoting a deeper understanding of the open world.

## Results

### TABS: a high-spatiotemporal-resolution distributed Brillouin fiber sensor

TABS represents an TAW-based Brillouin distributed fiber sensor, as illustrated in Fig. 1a. The optical fiber, serving as the sensing medium, is deployed on various objects in the open-world environment. TABS detects and localizes the sensing information along the fiber through a distributed transient acousto-optic interaction, as illustrated in Fig. 1b1–b3.

The SBS, which forms the physical foundation of TABS, involves a coupling interaction between two optical waves and an acoustic wave^10–14^. When two counter-propagating optical waves, termed pump and probe waves, with a frequency offset close to Brillouin resonant frequency (referred to as Brillouin frequency shift, BFS, *v*_*B*_), collide at a specific fiber position, local acoustic wave starts to stimulate, initiating energy transfer between the pump and probe waves, as shown in Fig. 1b1. The strength of acoustic wave determines the magnitude of pump-probe energy transformation. When the pump-probe interaction time is less than 10 ns, the acoustic wave remains in a transient regime (i.e., the TAW). The temporal and frequency-dependent evolution of the Brillouin gain under the TAW is illustrated in Fig. 1b2, with further details under the BFS condition provided in Fig. 2a. It can be observed that the SBS interaction under the TAW, i.e., the transient acousto-optic interaction, exhibits a *wideband* and *time-weighted* Brillouin gain evolution. The underlying mechanism is analyzed in Supplementary Section 3.

The design philosophy of TABS is to fully leverage the aforementioned two key physical properties of TAW through a synergistic framework to effectively utilize the Brillouin energy, thereby significantly improving overall sensing performance. Figure 1b3 shows the architecture of TABS, which consists of functionally synergistic components of transmitter (*Tx*.), fiber link, and receiver (*Rx*.).

The *wideband* Brillouin gain property of TAW is exploited in both the transmitter and receiver designs, enabling long-range high-temporal-resolution sensing. Specifically, in the transmitter, an optical pulse coding (OPC) pump wave with high robustness against detrimental effects is deployed to extend the sensing range. The wideband Brillouin gain property of TAW endows a more uniform and moderate pump-probe energy transformation over a wider frequency range compared to the SAW, as illustrated in Fig. 1b2. With this property, the TAW forms a perfect complementarity with the OPC: The low peak gain of TAW effectively suppresses the high peak accumulated gain and associated strong detrimental effects of OPC^17,18,35–38^, as illustrated in Fig. 1b3 and detailed in Supplementary Section 4. In turn, the OPC can be utilized with higher effective power of the pump and probe signals to markedly enhance the SNR, improving the measurement accuracy over a longer distance compared to the traditional B-DFOS, as detailed in Supplementary Sections 4 and 7. In brief, the TAW endows TABS with higher robustness against detrimental effects compared to the SAW, thereby allowing for an increase in signal power to enhance the SNR and measurement accuracy.

Simultaneously, in the receiver, a *wideband* Brillouin gain ratio (BGR) is employed to rapidly and precisely extract sensing information. Theoretical calculation reveals that the wideband BGS in the TAW retains nearly the same total spectral power as the narrowband BGS in the SAW, as shown in Supplementary Fig. S6c in Supplementary Section 5. This signifies that the wideband BGS features higher spectral energy than the narrowband BGS when the pump-probe frequency offset is far from the Brillouin resonant frequency, ensuring a wider frequency range for effective gain ratio. With this property, the BGR^45^ of TABS theoretically offers at least a 4-times wider linear region (measurement range) than that of the traditional B-DFOS, ensuring the measurement of large strain and temperature variations (200°C and 4000 με, or larger) with high fidelity and reliability, as detailed in Supplementary Section 5. Given this condition, TABS avoids the time-consuming multiple-point frequency scanning required for the narrowband BGS reconstruction in the traditional B-DFOS^12–14,20^. Instead, it only needs a simple and fast two-point frequency scanning to construct the wideband BGR, and extract the sensing information rapidly and reliably by analyzing the BGR variations, achieving high-temporal-resolution and large-measurement-range distributed sensing, as illustrated in Fig. 1b3 and detailed in Supplementary Section 6.

The *time-weighted* Brillouin gain property also plays a crucial role in achieving high-spatial-resolution distributed sensing. Specifically, in contrast to the SAW with a time-invariant envelope, the TAW exhibits a non-uniform time-varying envelope over the SBS interaction time, as illustrated in Figs. 1b2 and 2a. This time-varying acoustic wave intensity leads to time-weighted pump-probe energy transformation with most energy transferred during the latter half of the pump pulse, where the acoustic wave intensity approaches its peak. As a result, the effective SBS interaction length is shorter than the actual pulse width, as illustrated in Figs. 1b3 and Supplementary Fig. S10. This property allows TABS to break the spatial resolution limitations imposed by the physical bandwidth of signal generator. A higher spatial resolution can be achieved even with a relatively wide pump pulse (i.e., sub-pulse-width spatial resolution, as analyzed in Materials and methods, and Supplementary Sections 8 and 9). More importantly, as the achievement of high-spatial-resolution no longer relies on the SAW, TABS eliminates the need for using long optical pulses (tens of ns) to pre-activate the SAW—a common operation in the traditional high-spatial-resolution B-DFOS systems^20^. Consequently, the improvement in spatial resolution does not stimulate or aggravate detrimental effects, preserving the measurement accuracy and speed, as detailly analyzed in Supplementary Sections 8 and 9.

The sensing performance of TABS under various operational conditions has been theoretically analyzed and predicted. Figure 2b presents the intrinsic measurement ranges across different pulse widths (*w*_*P*_) and dual-slope frequency spacings (∆*f*_*DS*_), as detailed in Supplementary Sections 5 and 9. By reducing the *w*_*P*_ and ∆*f*_*DS*_, the intrinsic linear region of the BGRS can be expanded from 120 MHz to 560 MHz (i.e., temperature and strain measurement ranges of 560 °C and 11200 με, respectively). Figure 2c depicts the spatial resolutions achieved with different *w*_*P*_ values, indicating that TABS can achieve sub-pulse-width spatial resolution with varying pulse widths (Materials and methods, and Supplementary Section 9). While adopting shorter *w*_*P*_ can improve both intrinsic measurement range and spatial resolution, a higher SNR is needed to maintain the same measurement accuracy, as illustrated in Fig. 2d and detailed in Supplementary Fig. S15. Notably, TABS features increased robustness against detrimental effects as the *w*_*P*_ decreases, which allows for further enhancements of SNR and key sensing metrics, as detailly analyzed in Supplementary Section 9.

### Sensing performance of TABS

The sensing performance metrics of TABS, including measurement range, sensing distance, measurement accuracy, spatial resolution, and temporal resolution, have been evaluated experimentally. Figure 2e illustrates the system configuration with practical platform shown in Supplementary Fig. S1 in Supplementary Section 1. The system is properly designed and optimized to obtain a good signal quality over a ~ 50 km long sensing distance. Detailed system design principles are discussed in the Materials and methods, and Supplementary Sections 1, 4 to 8.

The acoustic wave plays a central role in the pump-probe energy transformation and overall sensing performance. Consequently, the first step of the experiment involves measuring the real-part of the acoustic wave temporal envelope and comparing it with theoretical predictions, as shown in Fig. 2f, while Fig. 2g shows their high degree of similarity. The analysis of the acoustic wave temporal envelope is provided in Materials and methods, and Supplementary Section 2. Notably, the experimental waveform closely matches the theoretical one, achieving a similarity of beyond 99%, which underscores the precision and reliability of the theoretical calculations for the acoustic wave and sensing performance predictions above.

Subsequently, we assess the sensing results under specific operational conditions (*w*_*p*_ = 6.8 ns, ∆*f*_*DS*_ = 80 MHz) to further validate the system performance of TABS. As the linear range of BGRS determines the sensor measurement range, Fig. 2h shows the BGRS at 35 km and 49.25 km (fiber far-end) with 1900 signal averages. In agreement with the theoretical analysis in Supplementary Section 5, the BGRS exhibits a 200-MHz wide intrinsic linear region. Correspondingly, TABS can acquire the sensing information rapidly with wide temperature and strain measurement ranges of 200°C and 4000 με, respectively.

The measurement accuracy at each fiber location is evaluated by the measurement uncertainty distribution presented in Fig. 2i. Figure 2j illustrates the measurement uncertainty at 35 km and 49.25 km under various pump-probe frequency offsets. The measurement accuracy is directly proportional to the SNR, while inversely proportional to the frequency offset. To reach high measurement accuracy across a wide frequency range, it is essential to maintain a sufficiently high SNR, as detailed in Supplementary Section 7. Fortunately, TABS demonstrates significant robustness against detrimental effects inherent in the cascaded SBS process. The total pump and probe power can be further increased to enhance the SNR throughout the sensing fiber, achieving the SNR as high as 17.2 dB even at the fiber far-end, as depicted in Supplementary Fig. S5h in Supplementary Section 4. As a result, the measurement uncertainty along the whole sensing fiber remains below 2.7 MHz within a wide measurement range of ~100 MHz. This indicates that with an uncertainty of 2.7 MHz, the measurement range across the whole sensing fiber exceeds 100 MHz (but <200 MHz), corresponding to the temperature and strain measurement ranges larger than 100 °C and 2000 με, respectively.

After validating the measurement range and accuracy, a static temperature measurement is conducted to evaluate the spatial resolution of TABS. The test configuration is shown in Supplementary Fig. S11a in Supplementary Section 8. Three fiber sections, located at 49.25 km, with lengths of 1.7 m, 45 cm, and 45 cm (65 cm spacing) are inserted into a heater set to 95 °C, while the room temperature is 15 °C. Figure 2k displays the measured BFS distribution near the hot spots with a sample rate of 1 GSa·s^-1^, where the spatial resolution is determined to be 37 cm, matching the theoretical calculations illustrated in Supplementary Fig. S11b, c. The theoretical and experimental results demonstrate that a higher spatial resolution (37 cm) is indeed achieved under a wide pump pulse (6.8 ns), due to the time-weighted gain property of TAW.

Another test is implemented to evaluate extreme sensing capability of TABS when the HSs are shorter than the spatial resolution (37 cm), as illustrated in Fig. 2l and Supplementary Fig. S12a in Supplementary Section 8. Four fiber sections, located at 49.25 km, with lengths of 20 cm, 30 cm, 40 cm, and 1.5 m, are inserted into the heater (95 °C). Both theoretical simulations and experimental results reveal that TABS exhibits a good adaptability under the extreme conditions, owing to the time-weighted gain property of TAW, as illustrated in Supplementary Fig. S12. Specifically, when the HS lengths are 20 cm, 30 cm, and 40 cm, TABS can still detect 67%, 85%, and 92% of the true BFS variation, indicating its capability to sense small-size targets.

Moreover, a dynamic temperature measurement is performed to validate the dynamic response of TABS. The test configuration, shown in Supplementary Fig. S11a in Supplementary Section 8, includes a heater with a temperature that automatically increases from 15 °C to 95 °C. Figure 2m shows the measurement results, with Fig. 2n providing the details at positions z1 to z3. At a 1 GSa·s^−1^ sample rate, the one-shot measurement time is 70 s. TABS oversampled the temperature variation by a factor of 50, allowing for detailed recording and reconstruction of temperature evolution. In practice, however, a 300 MSa·s^-1^ sample rate (approximately 1-time sampling of the sensing signal with a 37 cm spatial resolution) is sufficient for acquiring key spatial points with effective sensing information. This leads to an one-shot measurement time (temporal resolution) of 21 s and the highest frequency response of 0.0238 Hz^46^.

Altogether, based on the TAW, TABS demonstrates a superior overall performance (sensing distance = 50 km, spatial resolution = 37 cm, temporal resolution = 21 s, temperature or strain measurement range ≥ 100 °C or 2000 με, and measurement uncertainty = 2.7 °C or 54 με). The sensing performance of TABS is compared with the prevailing high-spatial-resolution B-DFOS, as illustrated in Supplementary Table S2. Even over a longer sensing distance, TABS still achieves comparable spatial resolution and 1 to 2 orders of magnitude enhancement in temporal resolution compared to previous B-DFOS paradigms. As a case in point, compared to a Golay-coding-enhanced high-spatial-resolution Brillouin optical time domain analysis (BOTDA) sensor^27^, TABS achieves an approximately 53-fold increase in sensing speed while offering a longer sensing distance and a higher spatial resolution. Besides, the observed consistency between experimental and theoretical results for acoustic wave temporal envelope and key sensing metrics confirms the accuracy and reliability of theoretical predictions in Fig. 2b–d. This indicates that the performance of TABS can be flexibly adjusted and further enhanced by employing alternative operational conditions.

### TABS for state imaging of evacuated-tube maglev transportation system

With its long-range high-spatiotemporal-resolution distributed sensing capability, TABS holds promise for open-world sensing applications. As an exemplary application, TABS is explored for state imaging of key components in the evacuated tube transportation (ETT) maglev system.

The ETT represents a next-generation high-speed rail system that achieves speeds exceeding 1000 km·h^−1^ through the application of advanced magnetic levitation and evacuated tube technologies^47–50^. While the ultra-high speed significantly improves transport efficiency, the extremely short emergency response time makes early warning essential, particularly for critical infrastructures along the ETT line. However, the unique environment of the ETT—large span, high vacuum, strong electromagnetic interference, small internal space, non-transparent, and complex structure, presents significant challenges for conventional monitoring methods, such as remote sensing, infrared thermography, radar, Lidar, and other electrical sensors, as discussed in Supplementary Section 10. Given its sensing capability, TABS could offer a solution for this complex situation.

The first application involves state imaging of the evacuated tube body, a key component for maintaining air tightness of the ETT system^47–50^, as shown in Fig. 3a. The structure health of the evacuated tube body is critical for vehicle safety and stability, as it is constantly exposed to external atmospheric pressure, temperature variation, and mechanical forces over time. Here, TABS is explored to image the state of evacuated tube body for continuous health assessment. A test platform mimicking the ETT system is constructed for field trials, as digital photographs displayed in Fig. 3b, while Fig. 3c presents a schematic of the state imaging process by TABS.

The evacuated tube used in the experiment measures 2 m in length, 1 m in diameter, and 5 cm in thickness. A tight-jacketed sensing fiber cable (white in color) is deployed on the evacuated tube in a serpentine manner, as illustrated in Fig. 3b, c, with adjacent rounds spaced 5 cm apart. Detailed introduction to the sensing fiber cable is listed in Materials and methods section. The serpentine layout, together with the high spatial resolution of TABS, forms a passive sensor matrix^51^ that can not only image and pinpoint the abnormal events in a 3-dimensional (3-D) space, but also improves the ranging accuracy, which is vital to the automatic train control, as detailed in Supplementary Section 11.

During the test, three HSs are deployed on the evacuated tube to mimic anomalous events occurring along the ETT line, such as temperature changes or deformation. Each HS is controlled by a digital temperature controller, to vary individually and periodically from 30 °C to 65 °C at a room temperature of 20 °C. The HSs measure (45 × 13.5) cm^2^, (49 × 28) cm^2^, and (45 × 13.5) cm^2^ with center coordinates at (−42.34, 26.59, 32) cm, (0, 50, 79.5) cm, and (42.86, 25.74, 177) cm (with a 500 MSa·s^−1^ sample rate), respectively. Figure 3d shows the measured sensing information stream, while Fig. 3e provides the details. TABS successfully detected and localized the dynamic BFS variations induced by the three HSs. The zoomed-in view reveals a sawtooth waveform in the BFS variation, with a peak-to-peak change of ~35 MHz and a period of ~5 min, which matches the preset temperature and period of the HSs.

By mapping the fiber location to the tube location, the temperature distribution of the evacuated tube body is effectively imaged, as shown in Fig. 3g, h. Here, the 2-D (unfolded diagram, Fig. 3f, g) and 3-D (Fig. 3h) views of the tube state are displayed for visual clarity. The areas inside the white dotted boxes indicate the true locations of the three HSs, and the temperature-rise areas detected by TABS match well with these locations. The center coordinates are detected to be (−43.08, 23.85, 32) cm, (−6.94, 48.21, 79.5) cm, and (41.55, 26.14, 177) cm, respectively. Compared to the true center coordinates above, the maximal localization errors in the x-, y-, and z-directions are only 6.94 cm, 2.75 cm, and 0 cm, respectively. Detailed localization method is presented in Supplementary Section 11.

Notably, the fiber BFS also exhibits a linear relationship with the strain variation^12–14^. In practical, TABS can sense the temperature and strain simultaneously through a multiparameter sensing fiber (or cable)^13,52^, enabling the analysis of local temperature and deformation of the evacuated tube. Moreover, the layout of sensing fiber cable is not limited to the serpentine pattern above. Exploring other layouts (such as a helical layout) and corresponding post-processing algorithms is valuable to further improve the sensing efficiency. Besides, the practical evacuated tube (metal)^48,49^ is more prone to thermal conduction, thermal diffusion, and the propagation of mechanical waves than the experimental evacuated tube (polymethyl methacrylate, PMMA)^53^. These properties of the metal tube may make more fiber sections be exposed to the temperature rise and mechanical waves, thereby enabling the multiparameter fiber sensors^13,52^ to detect and localize the heat, strike, vibration, and vacuum leakage sources more readily.

In addition to monitoring the evacuated tube body, TABS is explored for temperature-rise imaging of the primary windings of a long primary linear synchronous motor (LSM). Figure 4a, b illustrates the internal structure and sectional view of the ETT system, respectively, which is based on high-temperature superconducting (HTS, type-II superconductor) maglev trains^54–56^. The LSM is the key element of vehicle traction and braking, and consists of primary windings on the ground and secondary permanent magnets (induction plates) on the vehicle^54–56^.

The primary windings temperature rise^57^ may be a challenge for the LSM due to high driving power of vehicle and low heat dissipation of the ETT (Supplementary Section 11). To protect the LSM from potential damages caused by abnormal temperature rises, continuous monitoring is crucial. Figure 4c, d shows the photograph and schematic of test setup, respectively, with a metal plate and HSs mimicking the LSM primary windings. The mimic LSM and the HSs measure (200 × 76.5) cm^2^ and (15 × 30) cm^2^, respectively. A sensing cable is arranged in a serpentine manner, with a ~ 5 cm spacing, to mimic the layout of the LSM primary windings while imaging the LSM state. The measurement lasts 51.3 min to record the temperature-rise evolution, while validates the long-term stability of TABS. During the first 25 min, two HSs are placed at (15, 39.9) cm and (15, 140.5) cm (in the (x, z) coordinate), and their temperatures rise gradually from 15 °C to 80 °C. Between the 25^th^ and 51.3^th^ min, the two heated HSs (80 °C) are relocated to positions (53.4, 87.7) cm and (15, 183.2) cm. In this way, the responses to both continuous and instantaneous temperature rise events can be observed. The measured sensing information stream is shown in Fig. 4e, indicating that TABS can rapidly and precisely detect the temperature-rise events.

By mapping the fiber location to the LSM location, the temperature-rise distribution along the mimic LSM is imaged, as shown in Fig. 4f, g, and detailed in Supplementary Section 11. The detected temperature-rise areas closely match the true HS locations, as highlighted in the black-dotted boxes. The center coordinates of the temperature-rise events during *i)* 0^th^ to 25^th^ mins and *ii)* 25^th^ to 51.3^th^ mins are detected to be *i)* (12.7, 40.7) cm and (16.6, 140.5) cm, and *ii)* (52.1, 85.2) cm and (14.6, 180.7) cm, respectively. Compared to true center coordinates, the localization errors in the x- and z-directions are less than 3 and 2.5 cm, respectively. Meanwhile, the measured temperature-rise areas are larger than the preset ones, which is attributed to the high thermal conductivity of metal materials, as illustrated in Supplementary Fig. S19 in Supplementary Section 11. The peak BFS variations corresponded to the two HSs average at 62.1 MHz, which is 94.89% of the theoretical calculation of 65-MHz (65 °C temperature-rise), indicating a high measurement fidelity.

In addition to the aforementioned tangible ETT components, intangible negative air pressure distribution (vacuum degree distribution) along the evacuated tube is also critical to train safety. As the aerodynamic drag increases quadratically with the train speed, any deviation in the vacuum degree will generate additional reactive force and heat to the vehicle running at subsonic or supersonic speed^58^, potentially causing the vehicle body to tremor or deform, as described in Fig. 5a. Accordingly, it is essential to perform distributed vacuum sensing to detect and localize abnormal vacuum leakage events. Unfortunately, the optical fiber is naturally insensitive to the negative air pressure (vacuum degree).

To address this problem, a vacuum-sensitive array (VSA)-assisted Brillouin distributed vacuum sensing is proposed, as working principle illustrated in Fig. 5b. The sensing system configuration is the same as that of the TABS. While the VSA, consisting of cascaded vacuum sensitive units (VSUs), is deployed along the evacuated tube. Each VSU performs as a transducer that converts vacuum degree variations into fiber strain and BFS variations. This allows distributed vacuum sensing by harnessing the high vacuum-to-strain conversion rate of VSU combined with the frequency selectivity of SBS, as detailed in Supplementary Sections 12–14. It should be noted that, different from the distributed temperature (or strain) sensing that needs densely distributed sensing points, quasi-distributed VSUs is employed for distributed vacuum sensing due to the diffusivity of the air. The use of quasi-distributed VSUs also results in a discrete SBS interaction, as illustrated in Fig. 5b. Therefore, we adopt a strategy of combining long pump pulse with lowpass filtering to fully exploit the discrete pump-probe energy transformation property, and further enhance the SNR, as detailly introduced in Supplementary Sections 12–14.

The sensing performance is validated experimentally, as digital photographs of field test and real VSU are displayed in Fig. 5c. In this setup, twelve VSUs (15 cm in length, 1 cm in radius, and spaced at 10 m intervals) located at the end of the 49.25 km-long fiber are installed in the evacuated tube, as detailed in Supplementary Section 15. Figure 5d, e (top and side views) presents the measured Brillouin signals, which feature 12 distinct Brillouin gain peaks (BGPs) corresponding to the 12 VSUs, matching the theoretical results in Supplementary Fig. S22 in Supplementary Section 14. Despite the short length of the VSUs and a signal average number of only 500, the signal quality remains high due to the robustness of the system against detrimental effects (Supplementary Section 12).

Subsequently, the vacuum resolution is investigated. Figure 5f shows the BFS distributions for vacuum degrees ranging from 10% to 20% in 2% increments. The BFS variations across all VSUs are synchronously and linearly related to the vacuum degree. The vacuum sensitivity of each VSU is determined to be approximately 23.7 MHz·kPa^−1^, as illustrated in Fig. 5g. Moreover, the BFS uncertainties of the 12 VSUs average at 0.79 MHz, as illustrated in Fig. 5h. Based on the vacuum sensitivity and BFS uncertainty, the vacuum resolution is calculated to be 33.3 Pa (=BFS uncertainty/vacuum sensitivity), which is comparable to that of the commercial high-precision point barometer (10 Pa resolution).

The dynamic response to vacuum variations is then evaluated, where the one-shot measurement time is 2.4 mins. Figure 5i shows the measured BGSs and extracted BFSs of the first and last VSUs in the dynamic vacuum measurement (measurement results of all VSUs are illustrated in Supplementary Fig. S23 in Supplementary Section 15). The temporal evolutions of the vacuum degrees are consistent, demonstrating high repeatability under dynamic vacuum conditions.

The measurement range of the VSUs is further tested under different vacuum degrees, as shown in Fig. 5j. Three different VSUs, with inner air pressures set to 100%, 83.3%, and 75% of atmospheric pressure (AP), are fabricated to assess the vacuum measurement range (see Supplementary Fig. S24 in Supplementary Section 16 for details). It can be observed that the vacuum measurement ceilings for these VSUs are 45%, 80%, and larger than 90%, respectively. Accordingly, by designing the VSU with proper inner air pressure, the system can be adapted to meet different vacuum sensing requirements.

The series of experimental evaluations on the exemplary ETT application showcase the feasibility and flexibility of TABS for complex open-world perception tasks with tangible and intangible instances.

## Discussion

Taking advantage of the low attenuation of optical fiber and high energy transformation of stimulated Brillouin scattering, Brillouin distributed fiber optic sensing (B-DFOS) has emerged as a promising technique for long-range environment perception. Nevertheless, it remains limited by the inherent detrimental impacts of steady acoustic wave and insufficient utilization of Brillouin energy, leading to low spatial and temporal resolutions. Achieving exceptional performance across all key sensing metrics is a long-term pursuit.

In this paper, we report an exciting finding: transient acoustic wave, long ignored in distributed sensing, could overcome multiple key limitations in the Brillouin distributed sensors, and drive a significant leap in sensing performance. By properly designing a synergistic architecture to effectively leverage the time- and frequency-dimension properties of transient acoustic wave, TABS has achieved a 37-cm spatial resolution over a 50-km long sensing distance with 1 to 2 orders of magnitude improvement in temporal resolution compared to the prevailing B-DFOS paradigms. Future efforts to minimize the signal averaging time are promising for further enhancing the sensing speed.

The high-spatiotemporal-resolution sensing capability of TABS facilitates a transition of long-range high-spatial-resolution Brillouin sensing from static measurement to dynamic perception, unlocking a broader spectrum of real-world applications from natural hazards early warning^1–3^ to real-time infrastructure health assessment^4–6^. Besides, with the growing trend of intelligentization, TABS holds promise for accessing open-world information streams, empowering smart devices with stronger environment-perception and decision-making capabilities. Foreseeably, TABS would play a positive role in accelerating the arrival of the era of intelligent everything.

Our work preliminarily investigated a methodology for leveraging the time- and frequency-dimension properties of transient acoustic wave for building high-performance distributed Brillouin sensing. While the physical mechanisms and potential applications of the transient acoustic wave remain to be further explored. We believe that in the future, with more researches and deeper understanding to the transient acoustic wave, more advanced Brillouin sensors^12–14^, microscopy^59^, integrated photonic devices^60^, and so forth, will be developed to make greater contributions across diverse realms.

The research on ETT health monitoring is in its early stages. Future work on detecting and fusing multi-dimensional state information (such as temperature changes, tube deformation, vacuum degrees, and frequencies of mechanical waves) is desirable to further improve the accuracy of vacuum-leakage detection and localization, as well as the prediction and diagnosis of tube fatigue, aging, and defect. We believe that through collaboration across multiple fields, including sensing, materials science, and artificial intelligence, more advanced perception techniques will be developed to further ensure the safety of ETT system.

## Materials and methods

### System configuration of TABS

The schematic diagram and digital photograph of TABS system are shown in Fig. 2e and Supplementary Fig. S1, respectively. The CW light with a center wavelength of 1549.232 nm is emitted from a narrow linewidth (100 kHz) external cavity laser (Yenista OSICS T100) and then split into two branches by an 80:20 optical coupler.

In the upper branch, the optical signal with 20% of total power is firstly time-gated into a pulse-coded sequence by a semiconductor optical amplifier (SOA, Opeak PLS-LSM-SOA-1550-8) driven by an arbitrary function generator (AFG, Tektronix AFG3000). An aperiodic code^17^ optimized by a correction algorithm^18^ is applied to enhance signal-to-noise ratio (SNR). The effective energy enhancement factor of the aperiodic code is 95^17,18^. The resulting coding gain (i.e., the SNR increment) is 8.3845 dB^17^. The effective pulse width and pulse interval are 6.8 ns and 40 ns, respectively. Then, the coded pulse train is amplified by a pulse Erbium-doped fiber amplifier (P-EDFA, Opeak EDFA-C-ZPL-ns-MB). After that, the polarization of coded pulse train is scrambled by two cascaded polarization scramblers (PS, Opeak OM-PS-R4, General Photonics PCD-003). Two cascaded polarization scramblers are adopted for ensuring a better state of polarization (SOP) coverage uniformity^61,62^ and long-term stability. Then, the coded pulse train is injected into the sensing fiber by a circular. The peak power of the coded pump pulse before injecting into the fiber is around 20 dBm. The sensing fiber in the experiment is a 49.25 km standard single-mode fiber which consists of two fiber sections with lengths of 10 km and 39.25 km, and Brillouin frequency shifts (BFS) of 10.8 GHz and 10.82 GHz, respectively.

In the lower branch, the optical signal with 80% of total power is firstly carrier-suppressed dual-sideband modulated by a Mach-Zehnder modulator (MZM) driven by a microwave generator (MG, R&S SMW200A). The microwave signal from the MG has a frequency of around 11 GHz which is close to the Brillouin gain region. Then, the upper and lower sidebands of the modulated light wave are separated and orthogonally polarized by an optical interleaver (IL1, FIBERWDM C-Band 50 GHz Interleaver) and two polarization controllers (PC1 and PC2), respectively. After that, the orthogonal dual sidebands are recombined using a polarization beam combiner (PBC) and injected into the sensing fiber by an isolator. The probe input power is −4 dBm.

After the pump-probe SBS interaction, the probe wave carrying the Brillouin signal reaches the receiver side through the circulator. In the receiver, the probe wave is firstly pre-amplified by an Erbium-doped fiber amplifier (EDFA, Amonics AEDFA-PA-30). Then, the probe wave is filtered by an optical band-pass filter (OBPF) to suppress the out-of-band amplified spontaneous emission (ASE) noise. After that, the dual sidebands of the probe wave are separated by another optical interleaver (IL2) and detected by a 350-MHz balanced photodetector (BPD, Thorlabs PDB435C). Finally, an oscilloscope (OSC, Lecroy Waverunner 8404 M) is used to convert the detected signal from analog domain to digital domain.

### Direct observation of real part of the acoustic wave temporal envelope

The acoustic wave is central to the working principle and sensing performance of TABS. Therefore, verifying the similarity of acoustic wave temporal envelope between the theory and experiment is crucial. However, due to the in-pulse energy accumulation, the Brillouin gains associated with different acoustic waves at various locations are superimposed and mixed, making direct observation of the real part of the acoustic wave temporal envelope challenging.

To directly observe the real part of the acoustic wave temporal envelope from the measured Brillouin gain distribution, we applied the experimental configuration shown in Supplementary Fig. S2a in Supplementary Section 2. A short fiber section (22 cm in length), located at the center of a 20-m long fiber (BFS ≈ 10.85 GHz), is installed on a strain-generation device consisting of a fixed stage and a moving stage, as illustrated in Supplementary Fig. S2b. The moving stage generates a significant strain of ~11600 με on the short fiber section, resulting in a local BFS change of ~580 MHz. The local BFS (11.4 GHz) is far away from the normal Brillouin gain region of the fiber (a region tens of MHz wide centered at 10.82 GHz). Giving this condition, when the pump-probe frequency offset is approximately 11.4 GHz, the SBS and energy transformation will occur only at the shorter strained fiber section. This setup avoids the cumulation of adjacent Brillouin signals, allowing the real part of the acoustic wave temporal envelope to be directly recorded by the Brillouin gain carried by the probe wave, as illustrated in Supplementary Fig. S2c.

Supplementary Fig. S3a shows the acoustic wave temporal envelopes (real part) at different frequency offsets for both theoretical calculations and experimental measurements. Supplementary Fig. S3b, c illustrates the top and side views, respectively. The pump-probe frequency offset is scanned from 11.25 GHz to 11.55 GHz in 1-MHz steps, with each Brillouin signal (i.e., the Brillouin gain trace) averaged over 50 measurements. The theoretical acoustic wave envelopes closely match the experimental results. By calculating the cosine similarity between the theoretical and experimental gain spectra at each pump-probe interaction time, we determine the similarities between the theoretical and experimental acoustic wave envelopes, as illustrated in Supplementary Fig. S3d. The average similarity across the entire interaction time is approximately 99%. Supplementary Fig. S3e provides details of the acoustic wave spectra at 1.8 ns, 4 ns, 6.4 ns, and 8.8 ns, indicating high consistency. Moreover, we investigate the acoustic wave temporal envelopes under shorter energy accumulation length by shortening the length of the strained fiber section to 12 cm, as illustrated in Supplementary Fig. S3f. The similarity between the theoretical and experimental acoustic waves remains around 99% on average, as shown in Supplementary Fig. S3g–i. All experimental results above have demonstrated that the theoretical calculations are precise, and the working principle and theoretical sensing performance predictions are reliable.

### Spatial resolution of TABS

The spatial resolution of TABS is determined by both the pump pulse width and transient acoustic wave temporal evolution, which could be represented as:

1 $$\Delta z=\gamma \frac{{v}_{g}{w}_{p}}{2}$$

where ∆*z* is the spatial resolution, *γ* is effective pulse width coefficient determined by the acoustic wave temporal evolution (0 < *γ* ≤ 1), *v*_*g*_ is the light group velocity in the optical fiber, *w*_*p*_ is the pump pulse width. TABS localizes the hot spots by using time-of-flight (TOF) method^20^, so its spatial resolution is partly determined by the pulse width (*w*_*p*_). While the time-weighted gain property of transient acoustic wave determines that at each fiber position, 1) the former half of the pump pulse primarily serves to excite the acoustic wave. Since the acoustic wave is still weak at this stage, the resulting pump-probe energy transfer is relatively low, 2) the latter half of the pump pulse contributes most to the energy transformation where the acoustic wave is relatively strong, as detailly analyzed in Supplementary Section 8. Therefore, the effective pulse width in TABS is shortened compared to the given pulse width (*w*_*p*_) by a factor of γ. In the theoretical simulations in Supplementary Section 9, the γ values are determined to be 0.5, 0.44, and 0.4333 for pulse widths (*w*_*p*_) of 7 ns, 5 ns, and 3 ns, respectively. This means that for the pulse width ranging from 3 ns to 7 ns, the spatial resolution of TAW-based TABS is averagely 2 times higher than that of the SAW-based high-spatial-resolution BOTDA.

### Structure of vacuum sensitive unit (VSU)

The VSU is mainly composed of an outermost rigid shell, an elastic polymer spiral inner shell, and the innermost sensing cable. The sensing cable is sealed in the polymer inner shell, while the polymer inner shell is contained within the rigid shell. Due to the constraint of the polymer inner shell’s spiral structure and the guidance of the outermost rigid shell, the vacuum-induced expansion of the polymer inner shell will mainly happen in the longitudinal direction, which causes longitudinal strain to the innermost sensing cable.

The vacuum-to-strain transfer efficiency (or equivalently, vacuum sensitivity) is mainly determined by the cross-sectional area and elasticity modulus of the polymer inner shell. Higher vacuum sensitivity can be reached by employing the polymer inner shell with higher cross-sectional area or lower elasticity modulus. The fabrication process of the VSU is detailed in Supplementary Section 13.

### Materials of the evacuated tube and sensing cable

The evacuated tube in the experiment is a self-design Polymethyl Methacrylate (PMMA) tube. The PMMA material is chosen in the experiment due to its good transparency, impact resistance, and high hardness. The vacuum degree in the evacuated tube is adjusted by a vacuum pump (Fujiwara 280pro) and measured by two high-precision barometers (Mingyu Instrument MY-JG150).

The tight-jacketed sensing cable (YOFC SF1011-A/900E, 900 ± 50 am in diameter) consists of innermost standard single-mode fiber and outermost ethylene-tetra-fluoro-ethylene (ETFT) tight jacket. The working temperature and sustainable strain of the sensing cable are −55 ~ 125°C and 20000 με, respectively.

## Supplementary information

Supplementary information

## Data Availability

The data that support the findings of this work are available from the corresponding author upon reasonable request.
