# Supplementary material for: High-spatiotemporal-resolution distributed Brillouin sensing with transient acoustic wave
Source: Light Sci Appl. 2025 Jun 3;14:210. doi: 10.1038/s41377-025-01848-4 (PMC12130268; doi:10.1038/s41377-025-01848-4)
Supplement: Supplementary file 1 — Supplementary information [file 41377_2025_1848_MOESM1_ESM.docx]

**Supplementary Information for**

**High-spatiotemporal-resolution distributed Brillouin sensing with transient acoustic wave**

Yin Zhou,^1,2,†^ Yuan Cheng,^3,4,†^ Jia Ye,^1^ Zonglei Li,^1^ Haijun He,^1^ Wei Pan,^1^ Bin Luo,^1^ Lianshan Yan^1*^

^1^Center for Information Photonics & Communications, School of Information Science & Technology, Southwest Jiaotong University, Chengdu, Sichuan, 610031, China.

^2^Current Affiliation: Department of Automation, Tsinghua University, Beijing 100084, China.

^3^School of Artificial Intelligence, Shanghai Jiao Tong University, Shanghai 200030, China.

^4^Department of Electronic Engineering, Tsinghua University, Beijing 100084, China.

^†^These authors contributed equally to this work.

*Correspondence to: [lsyan@home.swjtu.edu.cn](mailto:lsyan@home.swjtu.edu.cn)

**Contents**

[**Supplementary Section 1: System configuration of TABS** 1](#_Toc193145672)

[**Fig. S1** 1](#_Toc193145673)

[**Supplementary Section 2: Direct observation of real part of acoustic wave temporal envelope** 2](#_Toc193145674)

[**Fig. S2** 2](#_Toc193145675)

[**Fig. S3** 3](#_Toc193145676)

[**Supplementary Section 3: Introductions to conventional BOTDA and TABS** 4](#_Toc193145677)

[**Table S1** 6](#_Toc193145678)

[**Table S2** 11](#_Toc193145679)

[**Supplementary Section 4: Analyses of the robustness against detrimental effects** 13](#_Toc193145680)

[**Fig. S4** 14](#_Toc193145681)

[**Fig. S5** 18](#_Toc193145682)

[**Supplementary Section 5: Analyses of the Brillouin gain ratio spectrum under the TAW** 21](#_Toc193145683)

[**Fig. S6** 21](#_Toc193145684)

[**Fig. S7** 23](#_Toc193145685)

[**Supplementary Section 6: Analyses of the temporal resolution of TABS** 25](#_Toc193145686)

[**Supplementary Section 7: Analyses of the measurement accuracy of TABS** 27](#_Toc193145687)

[**Fig. S8**: 27](#_Toc193145688)

[**Fig. S9** 29](#_Toc193145689)

[**Supplementary Section 8: Analyses of the spatial resolution of TABS** 32](#_Toc193145690)

[**Fig. S10** 33](#_Toc193145691)

[**Fig. S11** 34](#_Toc193145692)

[**Fig. S12** 38](#_Toc193145693)

[**Supplementary Section 9: Analyses of sensing performance of TABS under other conditions** 40](#_Toc193145694)

[**Fig. S13** 41](#_Toc193145695)

[**Fig. S14** 43](#_Toc193145696)

[**Fig. S15** 46](#_Toc193145697)

[**Supplementary Section 10: Analyses of the ETT monitoring** 48](#_Toc193145698)

[**Fig. S16** 49](#_Toc193145699)

[**Supplementary Section 11: State imaging and localizing of ETT system by TABS** 51](#_Toc193145700)

[**Fig. S17** 51](#_Toc193145701)

[**Fig. S18**: 54](#_Toc193145702)

[**Fig. S19**: 57](#_Toc193145703)

[**Supplementary Section 12: Working principle of VSA** 58](#_Toc193145704)

[**Supplementary Section 13: Fabrication and sensitivity of VSU** 60](#_Toc193145705)

[**Fig. S20** 61](#_Toc193145706)

[**Supplementary Section 14: Acoustic wave stimulation in the VSA-assisted Brillouin sensing** 64](#_Toc193145707)

[**Fig. S21** 64](#_Toc193145708)

[**Fig. S22** 67](#_Toc193145709)

[**Supplementary Section 15: Details of the experiment of vacuum degree measurements** 69](#_Toc193145710)

[**Fig. S23** 69](#_Toc193145711)

[**Supplementary Section 16: Fabrication of VSU with variable vacuum measurement ranges** 71](#_Toc193145712)

[**Fig. S24** 71](#_Toc193145713)

[**References** 73](#_Toc193145714)

# **Supplementary Section 1: System configuration of TABS**

**
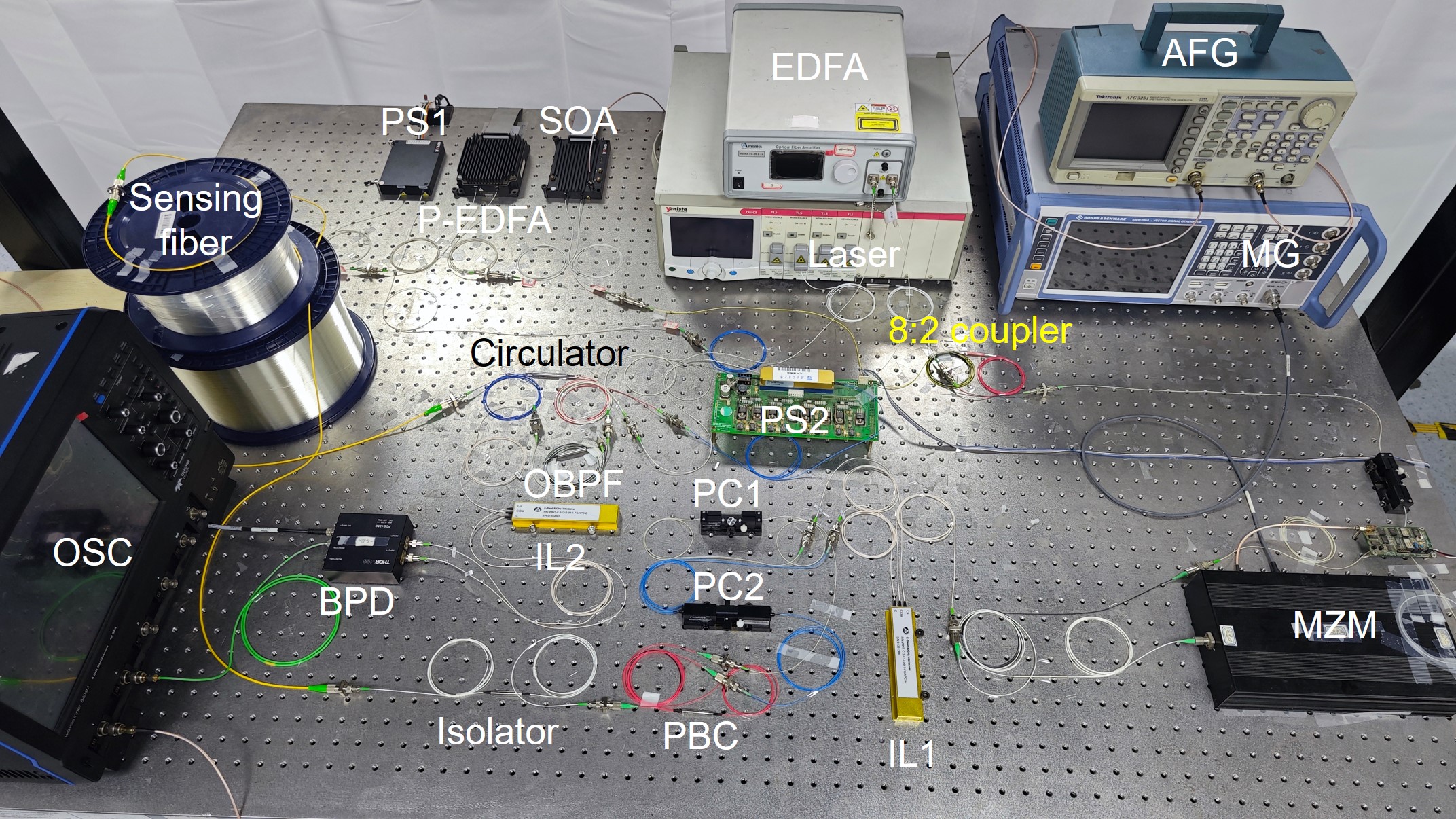
**

**Fig. S1: Experimental setup of the TABS sensing system.** SOA: semiconductor optical amplifier, AFG: arbitrary function generator, P-EDFA: pulse Erbium-doped fiber amplifier, PS: polarization scramblers, MZM: Mach-Zehnder modulator, MG: microwave generator, IL: optical interleaver, PC: polarization controllers, PBC: polarization beam combiner, EDFA: Erbium-doped fiber amplifier, OBPF: optical band-pass filter, BPD: balanced photodetector, OSC: oscilloscope.

# **Supplementary Section 2: Direct observation of real part of acoustic wave temporal envelope**

**
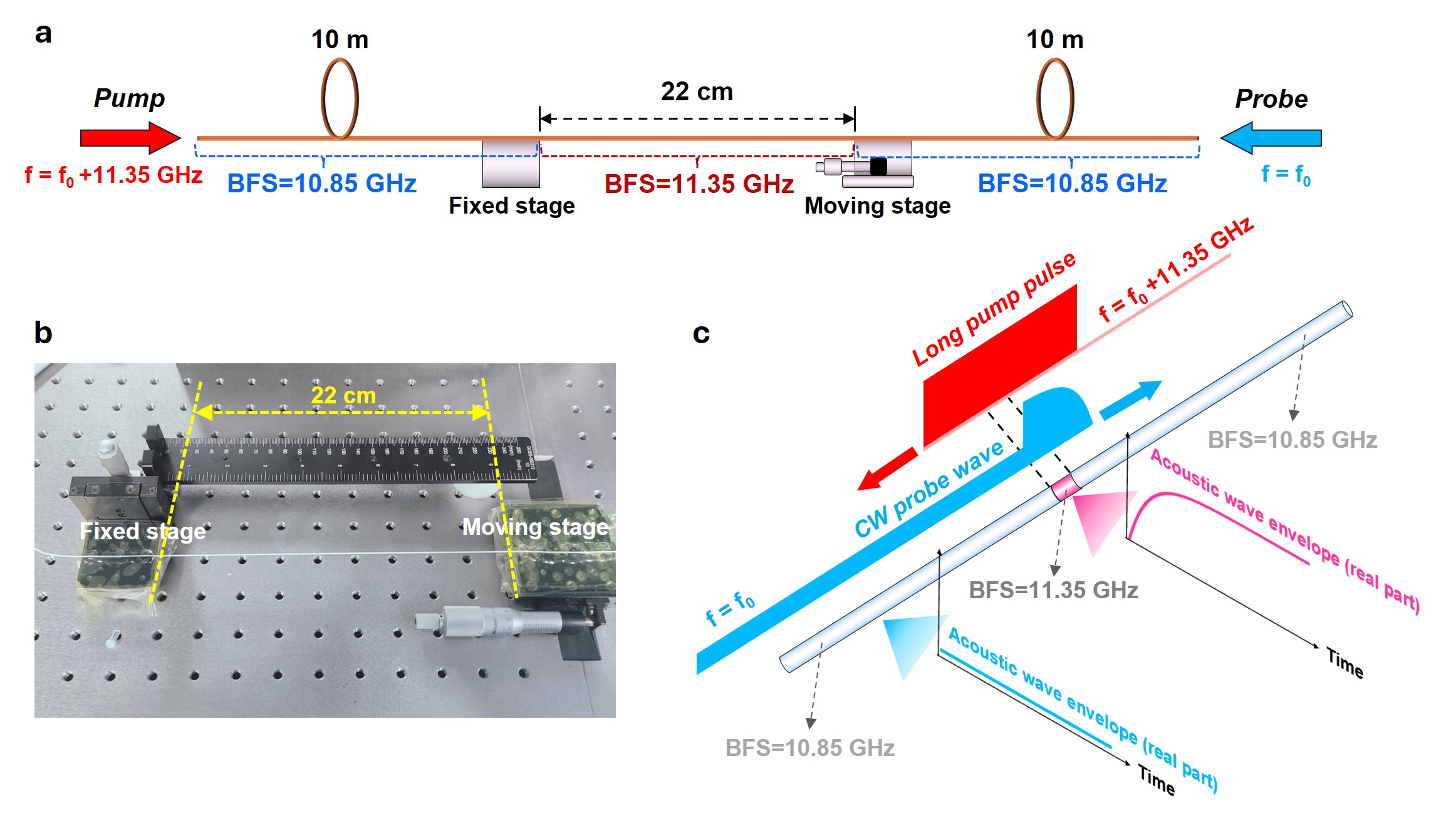
**

**Fig. S2: Experimental configuration and principle for** **direct observation of real part of acoustic wave temporal envelope. a,** Schematic diagram of experimental setup. BFS: Brillouin frequency shift (Brillouin resonant frequency). A large strain of ~11600 με is applied to a short fiber section with a length of 22 cm, which results in a large BFS change of 580 MHz. As a result, the BFS of strained fiber section is around 11.4 GHz which is far way from the normal BFS (~10.82 GHz) of the adjacent fiber (10 m in length).**b,** Digital photograph of fiber strain generating device which consists of fixed and moving stages. **c,** Working principle. The real part of acoustic wave temporal envelope determines the Brillouin gain evolution. As mentioned in (a), the short strained fiber section creates a large BFS change. Due to he frequency selectivity of the SBS, the energy transformation and accumulation will only occur at the short strained fiber section when pump-probe frequency offset is in the vicinity of 11.4 GHz. With the prevention of energy accumulation in adjacent long fiber sections, the acoustic wave temporal resolution (real part) can be recorded and directly observed by the Brillouin gain temporal evolution.


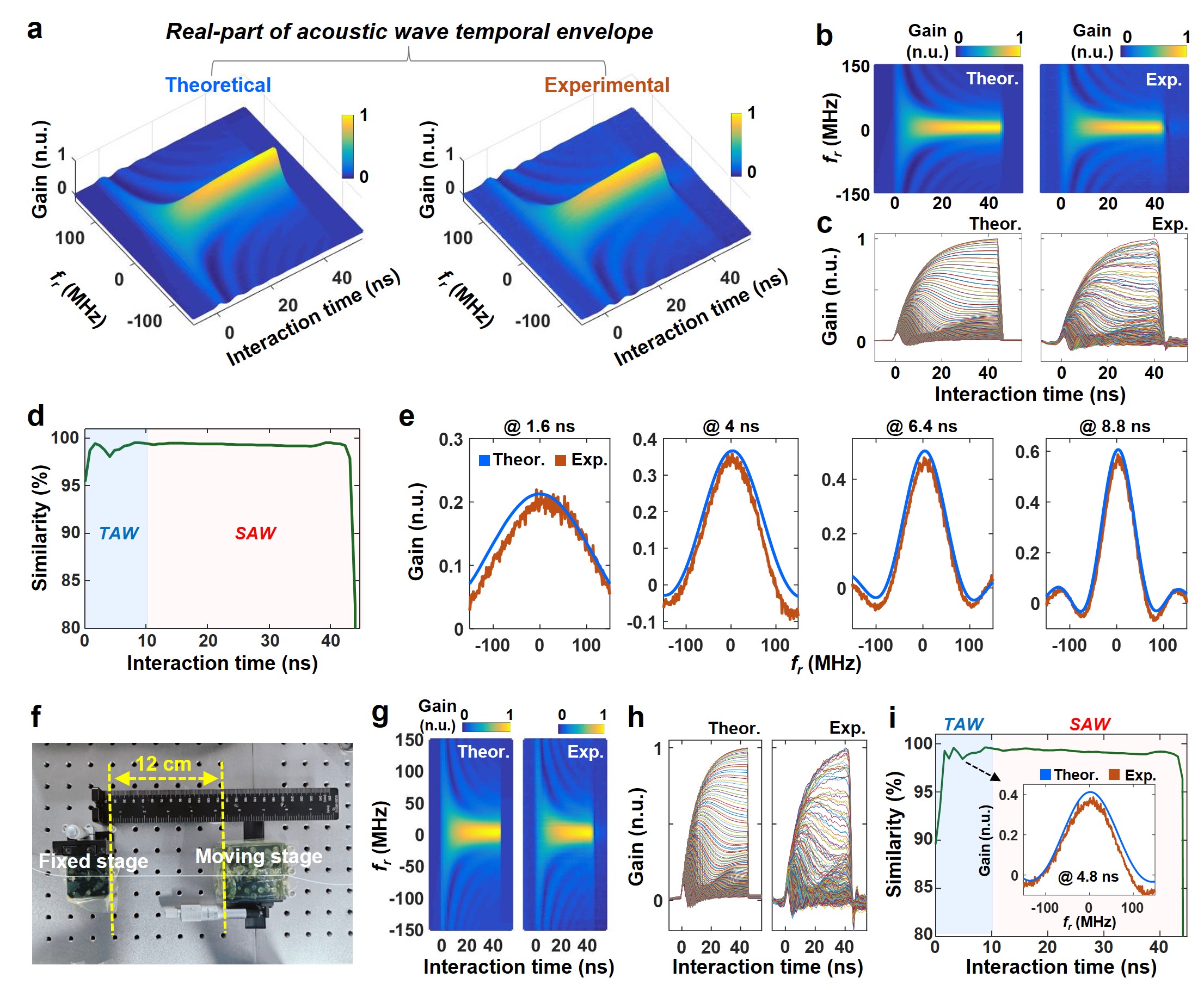


**Fig. S3: Direct observation of real part of acoustic wave temporal envelope. a,** Measured acoustic wave temporal envelopes (real part) in theory and experiment when the strained fiber section is 22 cm in length (corresponding to a 2.2 ns Brillouin energy accumulation time). n.u.: normalized unit, *f_r_*: relative frequency. **b, c,** The (b) top and (c) side views of (a). Theor.: theoretical, Exp.: experimental. **d,** Similarity of gain spectra between theory and experiment at different pump-probe interaction times. **e,** details of the gain spectra at different times. **f,** Digital photograph of the test configuration when the strained fiber section is 12 cm in length. **g, h,** The (g) top and (h) side views of the acoustic wave temporal envelopes (real part) when the strained fiber section is 12 cm in length (corresponding to a 1.2 ns Brillouin energy accumulation time). **i,** Similarity of gain spectra between theory and experiment at different pump-probe interaction times. The inset illustrates the gain spectra in theory and experiment at 4.8^th^ ns where the similarity is relatively low.

# **Supplementary Section 3: Introductions to conventional BOTDA and TABS**

In **Supplementary Section 3**, the differences between conventional Brillouin optical time domain analysis (BOTDA, a representative B-DFOS paradigm) and the proposed TABS are introduced for a better comparison on both principle and performance. Benefitting from high energy transformation of stimulated Brillouin scattering (SBS), BOTDA has shown great advantage in long-distance distributed sensing^12^. However, BOTDA remains hard to reach long range, high spatial resolution, high speed, and high accuracy simultaneously, due to multiple detrimental effects from steady acoustic wave (SAW).

Specifically, the SAW has been widely employed in nearly all existing B-DFOS paradigms (including the BOTDA) for almost thirty years since they were first proposed. The main reason for the long-standing choice is that the SAW can generate narrow Brillouin gain spectrum (BGS) that supports high Brillouin frequency shift (BFS) estimation accuracy when using curve fitting (CF) method^15, 63^. However, more recent researches have found that the use of SAW also brings some drawbacks as follows:

**(i) Strong detrimental effects:** The SAW leads the pump-probe energy transformation to occur intensively in the vicinity of BFS (i.e., Brillouin resonant frequency). As a result, the pump pulse is susceptible to pump distortion effect and systemic errors^35-37^. To alleviate the pump distortion, the pump and probe waves’ total power are both limited, which constrains the SNR and the ceiling of measurement range and spatial resolution. To improve the SNR and extend the sensing distance, optical pulse coding (OPC) methods^17, 23, 27^ have been proposed in recent years. Compared with distributed optical amplification^12, 19, 64, 65^ or digital signal denoising^66, 67^ methods, the OPC can be directly deployed in the current BOTDA framework and will not introduce additional optical noise or spatial resolution decrement in theory, thus it was treated as the promising way to reach significant improvement on the sensing performance. However, more recent investigations have revealed that the OPC may aggravate pump distortion effects^35-37^, introduce additional optical and electrical noises^17, 18, 38^,, and bring other detrimental impacts^68^ due to cascaded SBS interaction and stronger pump-probe energy transformation (see **Supplementary Section 4** for more detailed analyses). Consequently, the SNR increment gained by the OPC is severely degraded or even canceled out. The lack of sufficient robustness against the detrimental effects limits the system SNR and the measurement accuracy in long-range high-spatial-resolution sensing.

**(ii) Trade-off between spatial resolution and signal quality:** The stimulation of SAW requires a long optical pulse with tens of ns width. The long optical pulse leads to long in-pulse energy accumulation time and poor spatial resolution (several meters) which makes it hard to precisely localize the anomalous events. Meanwhile, the long optical pulse also leads to high peak Brillouin gain, especially in the OPC case, which further aggravates the detrimental effects and degrades the SNR.

**(iii) Low measurement speed:** The use of SAW requires a frequency scanning (FS) process to reconstruct the narrowband BGS, which slows down the measurement time^12, 32^. Fast BGS reconstruction methods including optical frequency comb^69^, orthogonal frequency division multiplexing^70^ and optical chirp chain^34^ methods have been proposed in recent years and demonstrated its effectiveness in accelerating the FS process and measurement speed. However, these methods need costly large-bandwidth arbitrary waveform generator (AWG) or other large-bandwidth radiofrequency (RF) devices to generate broadband and fast-switching RF signals^12, 34, 69, 70^, which may increase system costs and reduce their practical advantages in applications. Apart from the fast BGS construction methods above, slope-assisted (SA) methods are another way to accelerate the measurement speed^33, 71, 72^. However, affected by the detrimental effects mentioned above, the SNR of conventional BOTDA is limited, which makes the SA method hard to reach acceptable measurement accuracy just like the CF. Meanwhile, the measurement range supported by the SA methods was relatively small (tens of MHz in general), which may lead to signal distortions and unreliable results when faced with large temperature and strain variations in the applications.

Altogether, although numerous research efforts have been made to improve individual sensing performance to their upper bounds, it is still hard to improve the overall sensing performance by simply stacking these methods due to the trade-off among different methods and different detrimental effects, as summarized in **Tab. S1**.

**Table S1: Comparison of overall sensing mechanism with conventional high-spatial-resolution B-DFOS systems**

| **B-DFOS** | **Type of acoustic wave** | **Energy transformation manner** | **BGS property** | **Characteristics** |
| --- | --- | --- | --- | --- |
| **High-SR BOTDA**  ^20-23, 27-29^ | Steady | - Continuously^Ⅰ^ - Intensively^Ⅱ^ - Uniform gain^Ⅲ^ | - Narrowband - High Ac. G_B_ | - Susceptible to DE^Ⅳ^ - Multiple-point FS for reconstructing the BGS^Ⅴ^ - BGS fitting for extracting the BFS^Ⅵ^ - Pulse width-dependent SR^Ⅶ^ |
| **BOCDA**  ^24-26, 30^ | Steady | - Discretely^Ⅰ^ - Intensively^Ⅱ^ - Uniform gain^Ⅲ^ | - Narrowband - Low Ac. G_B_ | - Robust to DE ^Ⅳ^ - Multiple-point FS for reconstructing the BGS^Ⅴ^ - Multiple-point SS for reconstructing spatial information^Ⅴ^ - BGS fitting for extracting the BFS^Ⅵ^ - Bandwidth-dependent SR^Ⅶ^ |
| **TABS**  (This work) | Transient | - Continuously^Ⅰ^ - Dispersively^Ⅱ^ - Nonuniform gain^Ⅲ^ | - Wideband - Low Ac. G_B_ | - Robust to DE^Ⅳ^ - Two-point FS for constructing the BGR^Ⅴ^ - Wideband BGR for extracting the BFS^Ⅵ^ - Sub-pulse-width SR^Ⅶ^ |

**Note:** Ac. G_B_: accumulated Brillouin gain.

^Ⅰ^Continuously (or discretely): The SBS interaction occurs continuously (or discretely) along the fiber.

^Ⅱ^Intensively (or dispersedly): The SBS interaction occurs intensively (or dispersedly) in the vicinity of narrowband (or wideband) Brillouin gain region.

^Ⅲ^Uniform (or nonuniform) gain: The Brillouin gain is uniform (or nonuniform) along the SBS interaction time.

^Ⅳ^DE: Detrimental effects, including first-, second- and high-order pump distortions^35-37^, polarization pulling^37^, polarization noise^17, 38^, backward Brillouin noise^38^, slow transient response of EDFA^68^, and quantization noise in the ADC^18^.

^Ⅴ^Signal measurement process: 1) Conventional SAW-based B-DFOS requires multiple-point frequency scanning (FS) to reconstruct the BGS, which leads one-time measurement to be too slow to response dynamic events; 2) Correction-domain-based BOCDA requires multiple-point spatial scanning (SS) to reconstruct complete spatial information, which introduced additional measurement time; 3) TABS only needs two-point FS and one-point SS to construct the BGR, which accelerates the one-time measurement significantly, and enables the dynamic measurements.

^Ⅵ^Signal post-processing process: 1) Conventional SAW-based B-DFOS requires time-consuming BGS demonstration approaches to extract the BFS distribution, which further slows down the system response speed; 2) TABS only needs simple and time-saving calculations to extract the BFS distribution from the wideband BGR, which maintains the advantage of the high-speed measurement.

^Ⅶ^SR: Spatial resolution. 1) The spatial resolution of conventional SAW-based B-DFOS strictly follows the pump pulse width (or equivalently, the bandwidth); 2) TABS can reach a short spatial resolution under a wide pulse width (i.e., sub-pulse width spatial resolution) due to the time-weighted Brillouin gain property.

In contrast to the conventional BOTDA, **TABS** leverages transient acoustic wave (TAW) to suppress the detrimental effects mentioned above, enabling the improvements in Brillouin-energy-utilization-efficiency and overall sensing performance (including sensing range, spatial resolution, temporal resolution (speed), and accuracy). The TAW has long been considered less suitable for the B-DFOS since it generates the BGS with larger Brillouin linewidth and lower peak Brillouin gain which is not suitable for CF-based BFS extraction. However, in this work, we find that **the TAW features special energy transformation modes** which can be leveraged to reach overall sensing performance improvement under a properly designed sensor architecture.

Specifically, the **mechanism** of SBS involves a coupling interaction between an acoustic wave and two counter-propagating optical waves (referred to as pump and probe waves). When these two optical waves, with a frequency offset close to the Brillouin resonant frequency (i.e., the BFS), collide at a specific fiber position, the pump-probe interference induces an acoustic wave (a periodic density wave) through electrostriction and enhances the initial acoustic wave by resonating^10, 11^. The periodic density wave (i.e., the acoustic wave) induces a periodic variation in the fiber’s refractive index through the photoelastic effect, forming a moving grating. A portion of the forward-propagating pump wave is scattered by the moving grating. The scattered optical wave experiences a frequency shift by *∆*ν (equals to the frequency offset between the pump and probe waves, *∆*ν is close to the BFS) due to the Doppler effect and amplifies the backward-propagating probe wave (Stokes component). At this point, a single energy transformation between the pump and probe waves is completed.

Afterward, this cycle (i.e., the three-wave coupling interaction) continues until the pump pulse passes through this fiber section. Due to this cyclic interaction, within the first 10 ns (close to the phonon lifetime), new phonons are continuously generated, causing the acoustic wave to grow over time (TAW). This growth results in a time-varying Brillouin gain with increasing magnitude, i.e., the time-weighted gain property of TAW. After 10 ns, as the generation of new phonons and the annihilation of old phonons reach a dynamic equilibrium, the acoustic wave reaches a steady state (SAW), and the Brillouin gain becomes nearly time-invariant. Meanwhile, according to the time-bandwidth relationship^46^, the linewidth of the BGS narrows as the pump-probe interaction time increases, eventually approaching the intrinsic Brillouin linewidth (provided that the interaction time is sufficiently long)^10-14^. Accordingly, the wideband Brillouin gain occurs in the transient regime (TAW).

Collectively, when the pump-probe interaction time is less than 10 ns, the acoustic wave is stimulating and in the transient regime. In this regime, the acoustic wave (i.e., the TAW) offers *wideband* and *time-weighted* Brillouin gain evolution. These two factors are unique traits that can bring good innate conditions for overall performance improvement:

**(1) *Sensing distance and accuracy*:** The BGS broadening in the TAW case enables a more uniform and moderate energy transformation along a wider frequency range: The pump-probe energy transformation is decreased when pump-probe frequency offset is close to the BFS, while increased when the pump-probe frequency offset is far from the BFS. As a result, the pump distortion is largely suppressed, which allows higher probe power to improve the SNR. Meanwhile, the low peak gain of TAW also reduces the high accumulated Brillouin gain (Ac. G_B_) of the OPC. Consequently, the **detrimental effects** including high-order pump distortion, additional optical and electrical noises, and so forth **are all suppressed**, as detailly analyzed in **Supplementary Section 4**. Accordingly, the OPC sequence with higher coding length can be adopted to further improve the SNR, which improves the measurement accuracy or sensing speed (by reducing trace averaging number) over a long distance, as detailly analyzed in **Supplementary Section 7**.

**(2) *Measurement range and temporal resolution*:** the BGS broadening-induced spectral power spreading extends the frequency range for Brillouin gain ratio (BGR^45^). The linear region of Brillouin gain ratio spectrum (BGRS) can be significantly enlarged. Consequently, instead of the time-consuming BGS reconstruction via a multiple-point frequency scanning, the **BFS can be extracted reliably and rapidly** after a two-point frequency scanning and BGR analysis**,** which improves the measurement speed significantly, while does not need the expensive AWG, as detailly analyzed in **Supplementary Sections 5 and 6**.

**(3) *Spatial resolution*:** TABS can reach cm-level spatial resolution naturally since the pump pulse width is shorter than 10 ns. Meanwhile, the time-weighted intensity of the TAW is also a trait that can be exploited to further improve the spatial resolution of TABS. Specifically, the SBS is a three-wave coupling interaction process. In the traditional BOTDA, the envelope of steady acoustic wave is nearly uniform over the whole SBS interaction time. As a result, peak pump-probe energy transformation and accumulation persist over the time scale equal to the pulse width. As a result, the spatial resolution of traditional BOTDA equals *n_eff_***W_p_*/2 (*n_eff_* indicates effective refractive index, *W_p_* is the pulse width which relies on the bandwidth of electro-optical devices, including electrical and optical pulse generators). On the contrary, in TABS, the TAW features non-uniform temporal envelope along the pump-probe SBS interaction time, as illustrated in **Figs. 1b and 2a** in the main text. This kind of time-weighted acoustic wave temporal envelope results in time-varying pump-probe energy transformation: Most of the transferred energy to the probe wave is contributed by the latter half of the pump pulse where the acoustic wave intensity is closer to its peak. This means that the effective pulse width is shorter than the actual pulse width. This property enables a higher spatial resolution to be achieved by a wider pump pulse (**Sub-pulse-width spatial resolution**), which helps to alleviate the bandwidth requirements on electro-optical devices (including electric signal generator and electro-optical modulator) and reduce the system cost, as detailly analyzed in **Supplementary Sections 8 and 9**. More importantly, TABS bypasses the use of long optical pulse for stimulating steady acoustic wave that is commonly required in conventional high-spatial-resolution BOTDA systems. The spatial resolution improvement (up to cm-level) **will not stimulate or aggravate the detrimental effects**, which preserves the increments in measurement accuracy and speed, as detailly analyzed in **Supplementary Sections 4 to 7**.

Altogether, the wideband and time-weighted energy transformation of TAW enables TABS to reach high robustness against detrimental effects, as summarized in **Tab. S1**, and markedly improved performance on all key sensing metrics, including sensing range, spatial resolution, temporal resolution, and measurement accuracy.

The sensing performance of TABS is compared with that of the state-of-the-art (SOTA) high-spatial-resolution B-DFOS, as shown in **Tab. S2**. The researches in **Tab. S2** are representative works about high-spatial-resolution B-DFOS with SOTA performance. It can be observed that even under a longer sensing distance, TABS still achieves comparable spatial resolution and 1 to 2 orders of magnitude improvement in temporal resolution than the previous SOTA methods. Besides, the implementation of TABS is simple and costly, and does not need the expensive AWG device. The high sensing performance as well as the simple system configuration enable TABS to be a promising tool for remote dynamic measurement in diverse real-world applications.

**Table S2: Comparison with state-of-the-art (SOTA) high-spatial-resolution Brillouin fiber sensing systems**

| **Source** | **AW**  **type** | ***L_s_***  **(km)** | **SR**^Ⅰ^  **(cm)** | **Temporal resolution**^Ⅱ^ | | | | | | **σ*υ***  **(MHz)** |
| --- | --- | --- | --- | --- | --- | --- | --- | --- | --- | --- |
|  |  |  |  | ***N_SS_*** | ***N_FS_*** | ***N_Avg_*** | ***N_oper._***^Ⅲ^ | ***t_meas_***^Ⅳ^***.* (min)** | ***t_pp_***^Ⅴ^ |  |
| Approaches in time domain | | | | | | | | | | |
| Li et al.^21^ | S | 1 | 15 | 2 | 200 | 4000 | 1.6×10^6^ | N/A | Long | 2.6 |
| Soto et al.^23^ | S | 60 | 25 | 2 | 27 | 15330 | 8.3×10^5^ | N/A | Long | 1.2 |
| Li et al.^27^ | S | 10.16 | 50 | 2 | 100 | 1024 | 2.05×10^5^ | N/A | Long | 1.4 |
| Foaleng  et al.^22^ | S | 0.04 | 5 | 1 | 256 | 256 | 6.5×10^4^ | 8 | Long | 0.5 |
| Ge et al.^28^ | S | 4.9 | 50 | 1 | 40 | N/A | N/A | N/A | Long | 0.31 |
| Peng  et al.^29^ | S | 5.1 | 43 | 1 | 200 | 1000 | 2×10^5^ | N/A | Long | 1.5 |
| Dong  et al.^43^ | T | 2 | 2 | 2 | 33 | 1000 | 6.6×10^4^ | N/A | Long | 2 |
| Zhou  et al.^44^ | T | 0.011 | 0.8 | 1 | 125 | 1000 | 1.25×10^5^ | N/A | Long | 3.9 |
| Approach in expanded time domain | | | | | | | | | | |
| Youn  et al.^31^ | S | 0.22 | 2 | 52125^*^ | 200 | 16 | 1.67×10^8^ | ~6.67 | Long | 1.2 |
| Approaches in correlation domain | | | | | | | | | | |
| Denisov  et al.^25^ | S | 17.5 | 0.8 | 1023 | 100 | 512 | 5.2×10^7^ | ~189^#^ | Long | 1.8 |
| Zhou  et al.^30^ | S | 72.65 | 5 | 127 | 18 | 1800 | 4.1×10^6^ | ~445 | Long | 2 |
| This work in time domain | | | | | | | | | | |
| **TABS**  (This work) | **T** | **50** | **37** | **1** | **2** | **1900** | **3.8×10^3^** | **~0.35** | **Short** | **2.7** |

**Note:** AW: acoustic wave, S: steady, T: transient, *L_s_:* sensing distance, SR: spatial resolution, *N_SS_*: number of spatial scanning, *N_FS_*: number of frequency scanning, *N_Avg_*: number of trance averaging, *T_meas_*.: measurement time, *T_PP_*: data post-processing time (or interrogation time), συ: BFS uncertainty.

^Ⅰ^Spatial resolution is evaluated by the duration from 10% to 90% of the raising (or falling) edge^23^.

^Ⅱ^Temporal resolution describes sensor response speed which is reversely proportional to the measurement time (*t_meas._*) and data post-processing time (*t_pp_*).

^Ⅲ^*N_oper._* (=*N_SS_*×*N_FS_*×*N_Awg_*) describes the number of operations required to complete a one-time measurement. The temporal resolution (or equivalently, response speed) is reversely proportional to the *N_oper._*, as detailly discussed in **Supplementary Section 6**. Compared with the conventional SAW-based B-DFOS that requires multiple-point frequency scanning to reconstruct the BGS, TABS only needs two-point frequency scanning to construct the BGR, and complete one-time measurement, which markedly reduces the *N_oper._*, and enhances the temporal resolution.

^Ⅳ^Measurement time (*t_meas_*) is directly proportional to the *N_oper._*, sensing distance (*L_s_*), and ADC sample rate, as detailly analyzed in **Supplementary Section 6**.

^Ⅴ^Data post-processing time (*t_pp_*) is mainly determined by BGS demodulation time, as detailed in **Supplementary Section 6**.

*Estimated value: The equivalent conversion from time shifting to spatial shifting is based on the one-to-one correspondence between the received time trace and the sensing position of the BETDA^31^. Notably, unlike the BOCDA approaches, the BETDA does not require a spatial scanning process. While the time-expanding property of BETDA causes the sensing signal from all spatial points to be acquired after multiple time shifts, introducing additional measurement time. For simplicity, time shifting is converted to spatial shifting.

^#^Estimated value: In Ref. 25, a fully optimized measurement of 1,000,000 spatial points (i.e., sensing length = 17.5 km, spatial resolution = 17.5 mm, 100 scanned frequencies) takes 1.5 hours, assuming zero latency in the acquisition system. Therefore, the measurement of 2,100,000 spatial points (i.e., sensing length = 17.5 km, spatial resolution = 8 mm, 100 scanned frequencies) should take 3.15 hours (i.e., 189 minutes)..

# **Supplementary Section 4: Analyses of the robustness against detrimental effects**

To improve the sensing distance and measurement accuracy, it is crucial to enhance the robustness against detrimental effects, thereby increasing the SNR. The detrimental effects in the pump-probe SBS process can be classified into **1)** pump distortion effects (causing signal distortion and systematic errors), **2)** additional optical and electric noises (causing decrements on the SNR and measurement accuracy), **3)** other detrimental impacts caused by optical effects in system components (causing signal distortion and SNR decrement). In the following, we will gradually introduce these detrimental effects and analyze the robustness of TABS to these effects.

Firstly, **the robustness against pump distortion effects** is analyzed. The pump distortion effects^34-36^ result from the excessive absorption (pump depletion) or excessive transfer (pump excess-amplification) of pump pulse energy by the probe wave when pump-probe frequency offset closes to the average BFS of the sensing fiber. Due to the pump distortion effects, the pump pulse will be gradually distorted during its propagation in the optical fiber, which results in increasingly larger measurement errors as the pump pulse approaches the far end of the fiber, as illustrated in **Fig. S4a**. According to the excitation conditions and distortion characteristics, the pump distortion effects can be categorized into three types: first-order^35^, second-order^36^, and high-order^37^. The severity of these three effects is directly proportional to the probe power. Therefore, the most straightforward way to alleviate the pump distortion effects is to decrease the probe output power, which, however, sacrifices the SNR and accuracy. Besides, another way to alleviate the pump distortion effects is to use broadband linear frequency modulated probe waves^16^ that are generated by a large bandwidth AWG. However, the high cost and complexity of the AWG decrease the practicality of these approaches severely.

In contrast to the conventional B-DFOS that addresses the pump distortion effects by adjusting the optical waves, **TABS alleviates the pump distortion effects by leveraging the acoustic wave in the transient** **regime (i.e., the TAW)**. Specifically, in the transient regime, the acoustic wave is stimulating, and the pump-probe energy transformation occurs moderately across a wide frequency range, which prevents intensive pump depletion or excess amplification from occurring within a narrow frequency range. Since the TAW is generated by using the pump pulse with a width shorter than 10 ns, the suppression of the pump distortion effects can be achieved without


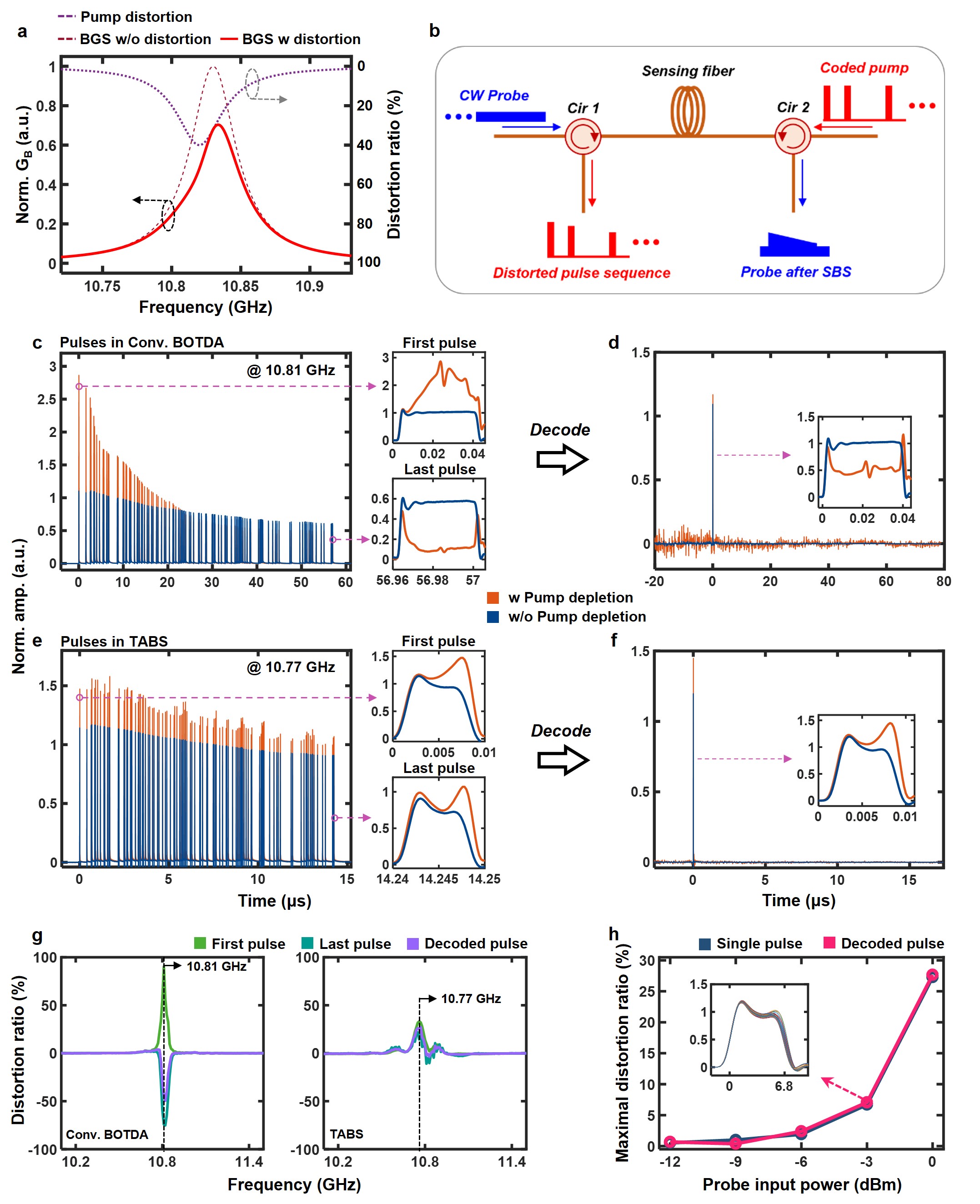


**Fig. S4: TABS’s robustness against detrimental effects. a,** Impact of pump depletion effect. Norm. normalized. **b,** Experimental configuration of pump distortion ratio measurement. **c, e,** Measured coded pump pulse sequences after propagating through the 50 km long fiber in (c) conventional coded-BOTDA and (e) TABS. Norm. amp.: normalized amplitude. **d, f,** Decoded single pulse responses in (d) conventional coded-BOTDA and (f) TABS. **g,** Pump distortion ratio comparison. **h,** Pump distortion ratio in TABS under single-pulse and OPC cases.

the need of decreasing the probe power or using the costly AWG, indicating a higher efficiency, lower cost, and higher practicality, as discussed above and analyzed below. An experiment is carried out to measure the pump distortions in SAW-based conventional BOTDA and TAW-based TABS. The experimental configuration is shown in **Fig. S4b**. The pump pulse sequence is measured at the probe wave output port, where the pump-probe SBS interaction is completed.

**Figures S4c and S4e** show the measured waveforms of pump pulse sequences in the conventional BOTDA and TABS, respectively. Here, a denoising aperiodic code^17^ with an energy enhancement factor of 95 is used to enhance the SNR. The denoising aperiodic code is generated by an aperiodic code correction algorithm^18^. The effective pulse widths (i.e., full width at half maximum, FWHM) in **Figs. S4c and S4e** are 6.83 ns and 39 ns, respectively. The pulse interval in **Figs. S4c** **and S4e** are 40 ns and 160 ns, respectively. Considering the time to construct steady acoustic wave and obtain the BGS with intrinsic linewidth in the classical high-spatial-resolution BOTDA^20^ (such as differential pulse-width pairs (DPP)^21^, acoustic wave pre-activation^22^, deconvolution algorithm^28^ and so forth), a pulse width of 40 ns is employed in the BOTDA. The probe output power is 0 dBm per sideband. The two inserts on the right side of **Figs. S4c** and **S4e** show the zoomed-in views of the first and last pulses of the coded pulse train, respectively. The distorted pump pulse sequences (red lines) in **Figs. S4c and S4e** are measured at the pump-probe frequency offsets of 10.81 GHz and 10.77 GHz, respectively, where the pump distortion reaches its respective peak. The pump pulse sequences without pump distortion (i.e., reference pulse sequences) are measured under the pump-probe frequency offset of 11.5 GHz which is far away from the SBS resonant frequency (i.e., the BFS) of the fiber. From **Fig. S4c**, it can be found that the coded pulse train is first excessively amplified before 10 us and then severely depleted after 10 us, due to the joint impacts of first-^35^, second-^36^, and high-order^37^ pump distortion effects. From the zoomed-in views on the right side of **Fig. S4c**, it can be observed that both the first and last pulses are severely distorted. The consequence of pump distortion in the time direction is the decoding noise, decoding error, and SNR degradation, as shown in **Fig. S4d**. The insert in **Fig. S4d** is a zoomed-in view of the decoded pulse. Evidently, the decoded single pulse is noisy, distorted, and depleted by ~50%.

On the contrary, the pump distortion effect is suppressed significantly in TABS. From **Fig. S4e**, it can be observed that the pump distortion is weak, and the pump pulse sequence is almost unchanged. Meanwhile, from the zoomed-in views on the right side of **Fig. S4e**, the first and last distorted pulses feature nearly the same shape as the reference pulses. As a result, the decoded pulse is clean and nearly undistorted, as illustrated in **Fig. S4f**, indicating a high decoding fidelity.

Subsequently, we further investigate the impact of pump distortion in the frequency direction, as illustrated in **Fig. S4g**. Here, the pulse sequences are measured under the sweeping frequencies from 10.1 GHz to 11.5 GHz with a 10 MHz step. **Figure S4g** shows the pump distortion ratios of the first, last, and decoded pulses in cases of conventional BOTDA (left side of **Fig. S4g**) and TABS (right side of **Fig. S4g**). The pump distortion ratio (*R_DST_*) is calculated by:

$R_{DST}=\frac{Max\left(P_{p}^{total}(f)\right)}{\left[\sum_{i=0}^{N} P_{p}^{total}(f_{ref}+i*\Delta f_{0})\right]/N}-1$ (S1)

$P_{p}^{total}(f)=\sum_{t=0}^{w_{p}} P_{p}(f,t)$ (S2)

where *Max*() denotes taking the maximum value, $P_{p}^{total}(f)$ represents the total power of decoded single pulse at each frequency offset, *P_p_*(*f*, *t*) is the decoded single pulse, *W_p_* represents the FWHM of the decoded pulse. *f_ref_* denotes a reference frequency point that is far away from the overall BFS, where the Brillouin gain and pump distortion are both 0. ∆*f_0_* is the frequency scanning step (the multiple-point frequency scanning is used here to record the pump distortion ratio at different frequency offsets). *N* is determined as 10 in this work for better suppression of random noise. From the left side of **Fig. S4g** (Conv. BOTDA case), it can be found that the maximal distortion ratios in the first and last coded pulses are around 90% (pump excess-amplification, mainly caused by second-order pump distortion effect^35^) and -75% (pump depletion, mainly caused by high-order pump distortion effect^37^), respectively. Consequently, the maximal pump distortion ratio in the decoded pulse is around -50%. In contrast, from the right side of **Fig. S4g** (i.e., TABS case), the maximal pump distortion ratios of the first coded pulse, second coded pulse, and decoded pulse are only 33%, 27%, and 27%, respectively. This demonstrates that the pump distortion effects in TABS are markedly alleviated, thanks to the suppression of Ac. G_B_.

Furthermore, we measured the pump distortion ratios in cases of single-pulse and coded-pulse-sequence under different probe output power, to observe the severity of pump distortion under different probe power more comprehensively. The measured pump distortion ratios under different probe output power are illustrated in **Fig. S4h**. The insert shows the decoded pulse at different pump-probe frequency offsets. It can be found that the pulse coding case has almost the same distortion ratio as that in the single pulse case. When the probe output power is -3 dBm per sideband, the distortion ratio is only ~7%, indicating a negligible impact on the measurement accuracy^35^. **The high robustness against the pump distortion effects enables the increment of probe output power and pulse coding length to reach a higher SNR**.

Apart from the pump distortion effects, there are **other detrimental effects** brought by the cascaded SBS interaction, including EDFA slow transient response^68^, polarization pulling^37^, additional optical and electric noises (O/E noises, including polarization noise^38^, signal-to-pump spontaneous Brillouin scattering wave beating noise^17, 18, 38^, quantization noise^18^ and so on). The impacts of all these detrimental effects are directly proportional to the Ac. G_B_ (representing the magnitude of pump-probe energy transformation)^17, 18, 35-38, 68^. In the conventional coded-BOTDA based on the SAW, the Ac. G_B_ reaches nearly 200% due to the intensive SBS interaction^18, 37^. The ultra-high pump-probe energy transformation magnifies the detrimental effects and introduces high O/E noises^17, 18, 38^ and distortions^68^ to the Brillouin signal. Consequently, theoretical SNR and sensing performance improvements from the OPC are severely degraded or even counteracted^17, 18, 35-38, 68^. On the contrary, in TABS, the pump energy transformation occurs moderately in a wider frequency range. Meanwhile, TABS can reach high spatial resolution without the need of long pump pulse for acoustic pre-activation. As a result, the peak pump-probe energy transformation is suppressed at least 4 times, as illustrated in **Fig. S6b** in **Supplementary Section 6**. In the experiment, the maximal linear Ac. G_B_ is reduced to only 29.5% (measured under the pump-probe frequency offset equals to the average BFS of the sensing fiber). Accordingly, **the detrimental effects including O/E noises and polarization-/EDFA-induced signal distortions are all alleviated significantly, which further improves the SNR**.

**
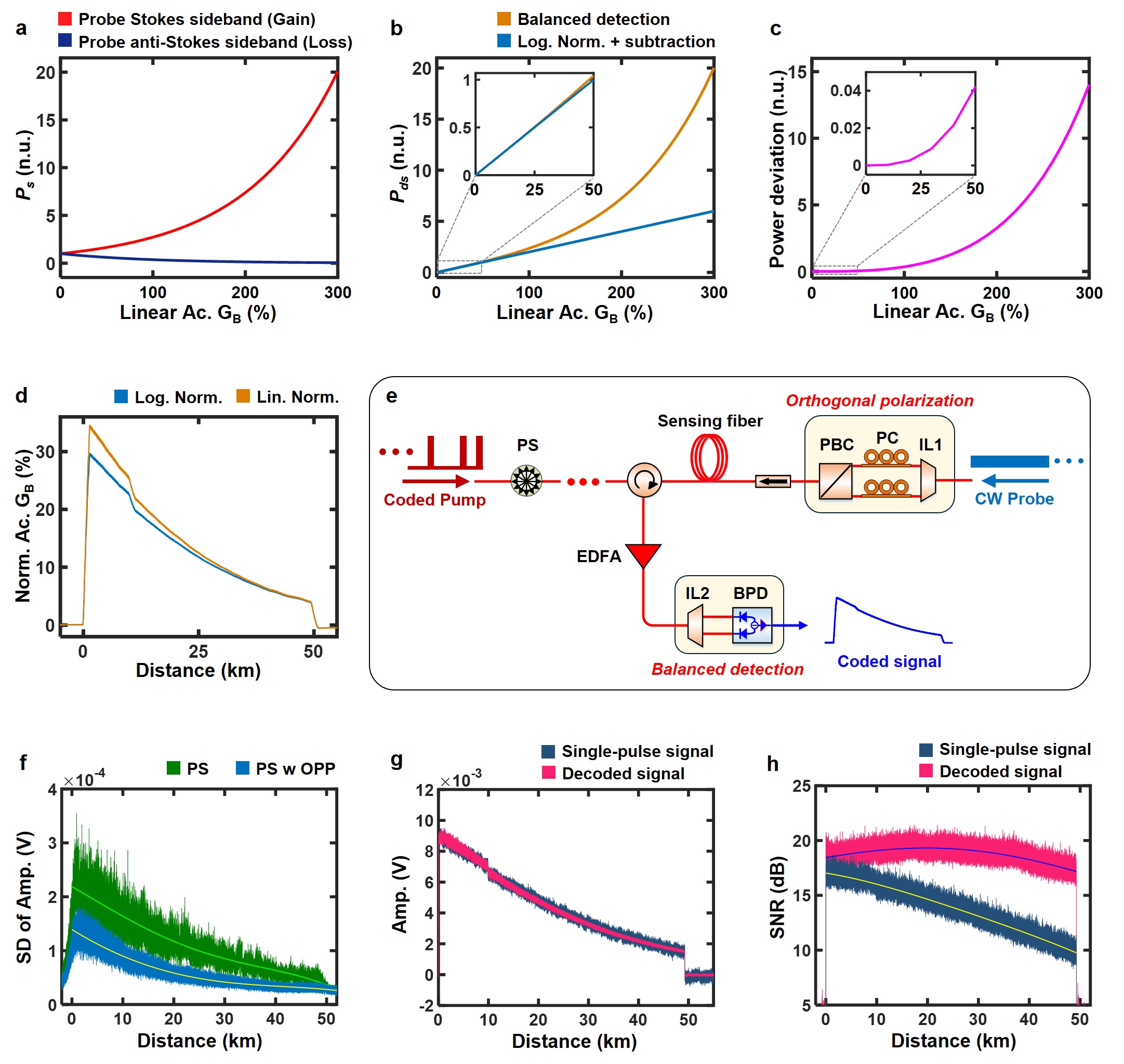
**

**Fig. S5: TABS’s robustness against optical noise**. **a,** Accumulated Brillouin gain and loss under different linear accumulated Brillouin gain and loss in theory. *P_s_*: signal power, n.u.: normalized unit, Ac. G_B_: accumulated Brillouin gain. **b,** Differential signals under linear normalization (i.e., Balanced detection) and logarithmic normalization (i.e., Log. Norm.+subtraction) methods. *P_ds_*: power of differential signal. **c,** Power deviation after balanced detection under different linear accumulated Brillouin gain. **d,** Measured and normalized accumulated Brillouin gain traces under different normalization methods. **e,** Experimental configuration of Brillouin signal measurement. PS: polarization scrambler, IL: interleaver, PBC: polarization beam combiner, EDFA: Erbium-doped fiber amplifier, BPD: balanced photodetector. **f,** Standard deviation (SD) value distributions under polarization fading suppressing methods. **g,** **h,** The (g) Brillouin gain traces and (h) SNR distributions of TABS under single-pulse and OPC cases.

The drastically decreased Ac. G_B_ also brings **two additional advantages** that conventional coded-BOTDA does not have:

**1) The probe signal power before photodetection can be markedly increased to approach photodetector (PD) saturation power, which improves the detection SNR significantly.** For instance, for the conventional coded-BOTDA with the same coding length in the experiment and a pulse width of 2 m, the linear Ac. G_B_ reaches around 200%. In this case, the probe signal power before injecting into the PD in TABS case can be 7.4 dB (e^2^/e^0.3^) higher than that in the coded-BOTDA case, which improves the SNR by nearly 7.4 dB accordingly^73^.

**2)** **Balanced detection and orthogonal probe wave schemes^67^ are allowed in TABS to further improve the SNR.** Specifically, as illustrated in **Fig. S5a**, the signal power carrying accumulated Brillouin gain and loss (i.e., Ac. G_B_ and Ac. L_B_) vary exponentially with the linear Ac. G_B_ and linear Ac. L_B_, respectively. For Ac. G_B_ larger than 100%, the Ac. G_B_ after balanced detection deviates from the actual linear Ac. G_B_ and becomes more and more severe, as described in **Figs. S5b and S5c**. Accordingly, to avoid decoding errors due to exponentially increased Ac. G_B_^37^, the balanced detection is not allowed in the coded BOTDA. However, it can also be found in **Figs. S5b and S5c** that the Ac. G_B_ deviation is lower than 4% when linear Ac. G_B_ is lower than 50%. In TABS, the peak linear Ac. G_B_ is only 29.5% and the peak Ac. G_B_ is 34.3% (matches with the theoretical value of 34.31%), as shown in **Fig. S5d**. In this case, the gain deviation is only 0.9%, indicating a negligible impact on the decoding accuracy. Therefore, the balanced detection and orthogonal probe wave schemes can be adopted to TABS, as described in **Fig. S5e**. The use of balanced detection can not only improve the SNR by 1.5 dB^74^ but also suppress the EDFA slow transient response. In the experiment, the EDFA slow transient response induced signal distortion is reduced from 12.76% (=0.0588V/0.0075V, Brillouin gain without balanced detection) to 1.38% (=0.144V/0.002V), indicating a 9.24-times reduced gain distortion. Besides, by combining the polarization scrambling with the orthogonal probe wave, the polarization noises along the fiber are further suppressed by 2.5 dB on average compared to the case where only the polarization scrambling is used, as illustrated in **Fig. S5f**, which further improves the SNR along the fiber by ~2.5 dB accordingly.

Furthermore, the decoding fidelity is investigated. **Figure S5g** shows the decoded Brillouin signal at 10.82 GHz (around the BFS). The Brillouin signal in the single pulse case is also shown for comparison. The decoded Brillouin signal features nearly the same shape as the single pulse response, indicating a high decoding fidelity.

Moreover, the SNR distribution along the fiber is calculated and shown in **Fig. S5h**. The SNR distribution shows a different evolution from the conventional coded-BOTDA. In the conventional coded-BOTDA, the Brillouin signal is severely polluted by the O/E noise. As a result, the coding gain is degraded and the SNR in the front part of the fiber is commonly lower than that in the single-pulse cas^17, 18, 37^. The OPC can bring significant SNR improvement only at the far end of the fiber. On the contrary, in TABS, thanks to the suppression of the detrimental effects, significant SNR improvement can be achieved along the whole sensing fiber, as shown in **Fig. S5h**. In comparison to the single-pulse case, the SNR is improved by 1.5 dB, 3 dB, 4.65 dB, 5.93 dB, 6.8 dB and 7.418 dB at 0 km, 10 km, 20 km, 30 km, 40 km, and 49.25 km, respectively. **The SNR at the fiber far-end is as high as 17.18 dB.**

All together, benefitting from the wideband and moderate energy transformation of TAW, TABS features significantly enhanced SNR due to **1)** suppressed pump distortion effects and thus higher effective power of probe wave, **2)** decreased Ac. G_B_ and thus *Ⅰ)* enhanced coding length (i.e., higher effective power of pump wave), *Ⅱ)* suppressed O/E noises, *Ⅲ)* improved PD input power (i.e., higher effective power of probe wave), and *Ⅳ)* allowance of the use of balanced detection and orthogonal probe wave to further suppress the O/E noise.

# **Supplementary Section 5: Analyses of the Brillouin gain ratio spectrum under the TAW**


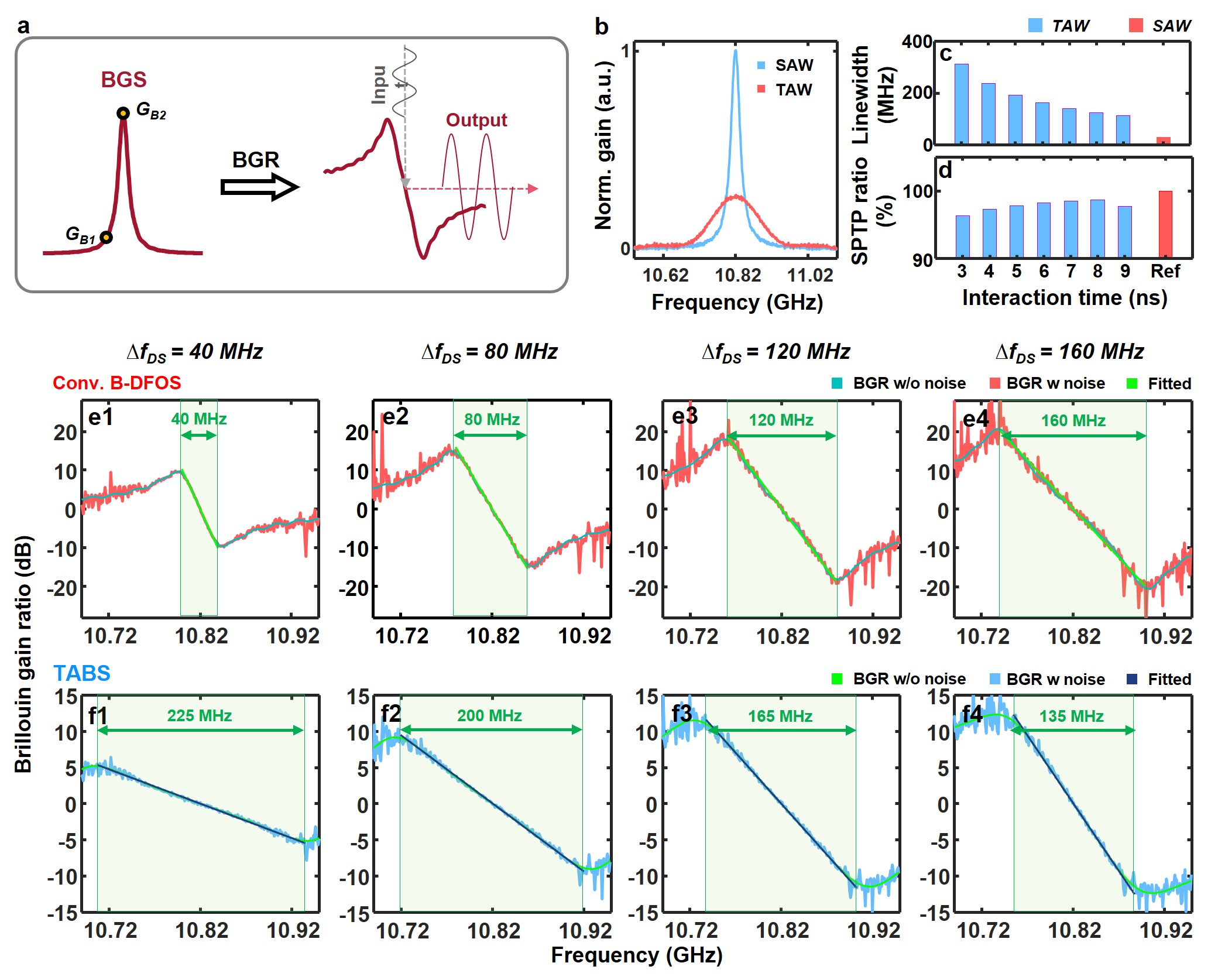


**Fig. S6: Brillouin gain ratio spectra under SAW and TAW in theoretical simulation. a,** Working principle of Brillouin gain ratio method. G_B_: Brillouin gain. **b-d,** Comparisons of (b) shapes, (c) linewidth widths, and (c) spectra total power (SPTP) between the BGSs in SAW and TAW cases. Ref: reference parameters in the SAW. **e,** **f,** Brillouin gain ratio spectra in (e1)-(e4) conventional BOTDA (bases on the SAW) and (f1)-(f4) proposed TABS (bases on the TAW) under different gain-ratio frequency spacing (∆*f_DS_*).

The Brillouin gain ratio (BGR) is a dual slope-assisted method which can extract the BFS value after two consecutive measurements by interrogating the ratio variation^45^. The Brillouin gain ratio spectrum (BGRS) at a certain location z is given by **Eq. S3** and illustrated in **Fig. S6a**:

$R_{B}\left(z,f\right)=\Re\left[10\cdot\log_{10}\left(\frac{G_{B}\left(z,f+\Delta f_{DS}/2\right)}{G_{B}\left(z,f-\Delta f_{DS}/2\right)}\right)\right]=\Re\left[10\cdot\log_{10}\left(G_{B2}/G_{B1}\right)\right]$ (S3)

where *R_B_*(*z*, *f*) and *G_B_*(*z*, *f*) represent Brillouin gain ratio (BGR) and Brillouin gain value at a certain location *z* and detuning frequency *f*, respectively. ℜ denotes taking the real part. ∆*f_DS_* is the frequency spacing between two Brillouin gain points. By dividing two Brillouin gains (*G_B2_* and *G_B1_*) with a specific frequency spacing Δ*f_DS_* and taking the logarithm, the BGRS with a certain linear region can be obtained. The linear relationship between the *R_B_*(*f*) and BFS is established by the linear region of the BGRS. The change in BFS can be directly extracted by analyzing the change in BGR value. The measurement range of the BGR method is determined by the width of linear region.

A numerical simulation is carried out to investigate the BGRS in cases of SAW and TAW. The simulation parameters are 1) BFS=10.82 GHz, intrinsic Brillouin linewidth=30 MHz, pulse width=6.83 ns (equals to the FWHM of pump pulse in the experiment). **Figure S6b** shows the BGSs in the SAW and TAW. Clearly, the BGS in the SAW features a narrower bandwidth and higher peak gain than that in the TAW. At first glance, the BGS of the SAW has a higher peak gain than that of the TAW, giving the first impression that the SAW supports higher energy transformation than the TAW. However, after calculating the total Brillouin gain along the whole frequency range, we find that, for the BGSs with different linewidths (**Fig.6c**), the **TAW features nearly the same spectrum total power as that in the SAW case**, as illustrated in **Fig. S6d**. This signifies that **the spectrum energy in the TAW spreads out to a wider range**. Then, the BGRSs in the SAW and TAW cases under the Δ*f_DS_*=40, 80, 120, and 160 MHz are calculated, as shown in **Figs. S6e1 to S6e4** and **Figs. S6f1 to S6f4**, respectively. Here, random noise is added to the BGS to simulate the realistic situation. The peak SNR in the SAW and TAW are 23.5 dB and 17.7 dB (nearly the same as the SNR at 49.25 km in the experiment), respectively. Notably, same random noise is added on both SAW- and TAW-based BGSs in the simulation, for simplicity of comparison. However, the actual noise in the SAW-based BGS in the BOTDA would be much higher than that in the TAW-based BGS in TABS due to stronger detrimental effects, as analyzed in **Supplementary Section 4** and summarized in **Tab. S1**. From **Figs. S6e and S6f**, it can be found that for all Δ*f_DS_*, the BGRSs in the TAW case feature a significantly wider linear range than that in the SAW. For different Δ*f_DS_*, the linear region in the SAW and TAW has opposite evolutions


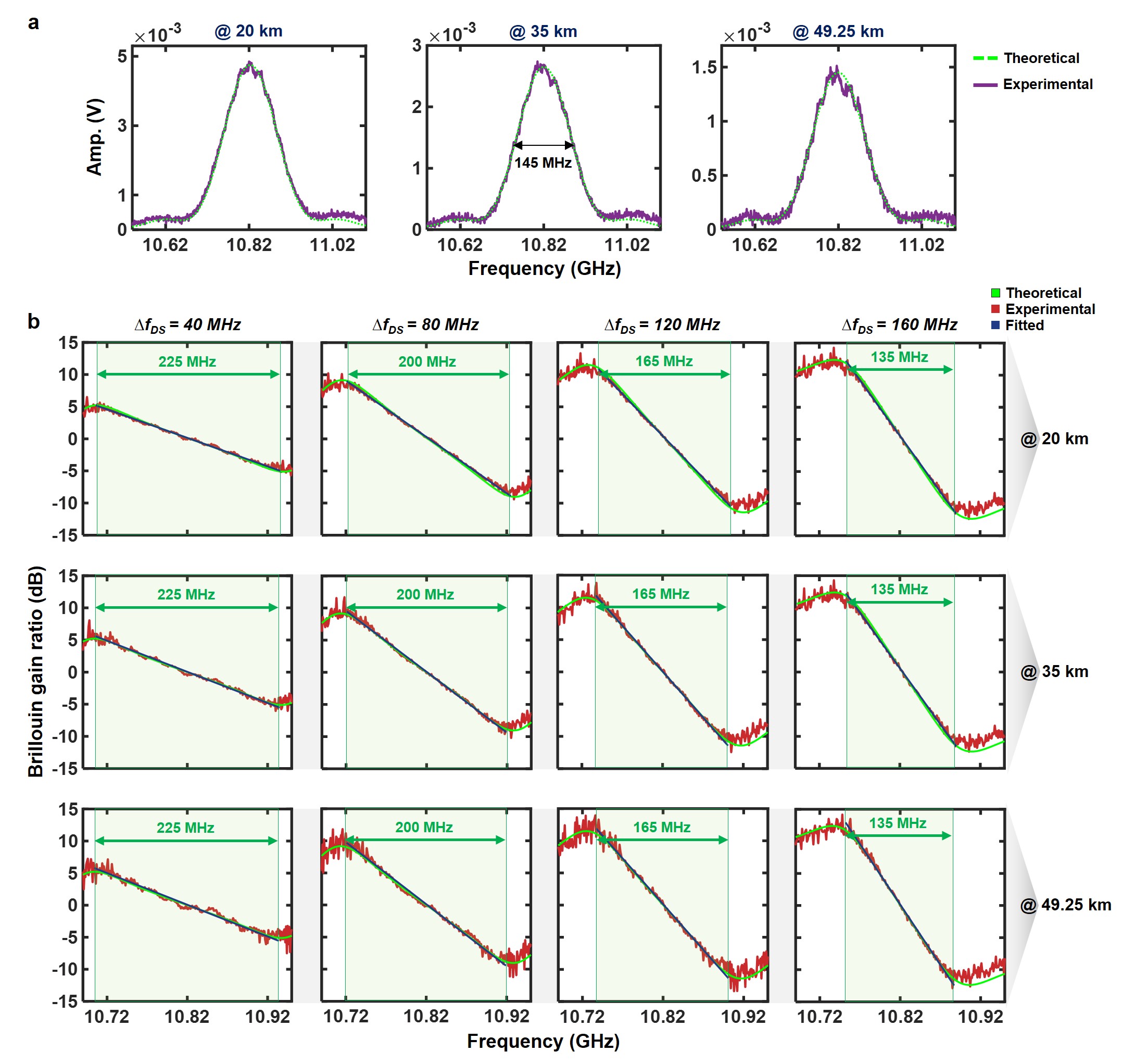


**Fig. S7: Brillouin gain ratio spectra (BGRS) of TABS in the experiment.** **a,** BGSs at different fiber locations. Amp.: amplitude. **b,** BGRSs under different Brillouin-gain-ratio frequency spacings and fiber locations.

due to different Brillouin linewidths. The linear region in the SAW increases with the increase of Δ*f_DS_*, but the noise after the BGR operation increases drastically due to the rapid decrement of Brillouin gain when the pump-probe frequency offset is away from the BFS. On the contrary, the linear region in the TAW decreases with the increase of Δ*f_DS_*. Meanwhile, thanks to the spectrum power spreading, there are still sufficiently high Brillouin gains for an effective BGR even though the pump-probe frequency offset is far away from the BFS. As a result, for the measurement range>100 MHz, the SNR after the BGR operation is higher than that in the SAW.

Furthermore, an experiment is carried out to validate the BGRS in the TAW. Here, the effective pump pulse width is 6.83 ns. **Figure S7a** shows the BGSs at 20 km, 35 km, and 49.25 km, respectively. Clearly, the measured BGSs match well with the theoretical BGS and feature an FWHM of 145 MHz. **Figure S7b** shows the BGRSs at different fiber locations and Δ*f_DS_*. Clearly, for different locations, the BGRSs feature nearly the same shape as the theoretical BGRS. The intrinsic linear regions of the BGRSs are indeed 225 MHz, 200 MHz, 165 MHz, and 130 MHz under the Δ*f_DS_* of 40 MHz, 80 MHz, 120 MHz, and 160 MHz, respectively. This indicates that the **TABS can support intrinsic strain and temperature measurement ranges as high as 4000 με and 200℃, respectively,** when the Δ*f_DS_* is 80 MHz. Altogether, by leveraging the spectrum power spreading in the TAW to enlarge the linear region of BGRS, large measurement range and high measurement speed can be achieved simultaneously. The temporal resolution of TABS is detailly analyzed in **Supplementary Section 4**.

After measuring the BGR distribution along the fiber at the selected frequencies (*f_sel_*+*∆f_DS_*/2 and *f_sel_*-*∆f_DS_*/2), the change in BFS distribution ∆*v_B_* can be extracted as follows:

$\Delta\nu_{B}\left(z\right)=\frac{\left[R_{B}^{0}\left(z,f_{sel}\right)-R_{B}\left(z,f_{sel}\right)\right]-b(z)}{m(z)}$ (S4)

where $R_{B}^{0}\left(z,f_{sel}\right)$ is the reference BGR distribution which is measured with a large amount of averaging (i.e., calibration) before performing the distributed sensing. *b*(*z*) and *m*(*z*) are the intercept on the y-axis (BGR axis) and the slope at different locations (z), respectively. The *b*(*z*) and *m*(*z*) are obtained by linearly fitting the $R_{B}^{0}\left(z,f_{sel}\right)$. The *b*(*z*) and *m*(*z*) are reusable after one-time measurement and linear fitting.

# **Supplementary Section 6: Analyses of the temporal resolution of TABS**

The temporal resolution represents the response time of the B-DFOS, which is determined by**:**

$T_{R}=T_{M}+T_{P}$ (S5)

$T_{M}=N_{SS}\cdot\left\{T_{SS}+N_{FS}\cdot\left[T_{FS}+N_{avg}\cdot\left(T_{RT}+T_{avg}\right)+T_{r\mathrm{e}c}\right]\right\}$ (S6)

$T_{RT}\geq2L_{s}/v_{g}$ (S7)

$T_{M}\propto N_{oper}=N_{SS}\cdot N_{FS}\cdot N_{avg}$ (S8)

$T_{P}=N_{SS}\cdot N_{FS}\cdot\left(T_{nor}+T_{DeC}\right)+T_{BGS-D}$ (S9)

where *T_R_*, *T_M_*, and *T_P_* represent the times for sensor response, measurement, and data post-processing, respectively. *N_SS_* is the number of spatial scanning, *T_SS_* is the spatial scanning time determined by a signal generator, *N_FS_* is the number of frequency scanning, *T_FS_* is the frequency scanning time determined by a microwave generator, *N_avg_* is the number of signal averaging, *T_RT_* is the light round-trip time of flight in the fiber (limited by fiber length), *T_avg_* is the single-shot signal averaging time (including the time for data acquisition, caching, and averaging) determined by data acquisition (DAQ) equipment and data volume, *T_rec_* is the data recording time (averaged data from the DAQ equipment to computer, including data transmission and storage time), *L_s_* is the length of sensing fiber, *v_g_* is light group velocity. ∝ denotes the direct proportionality. *N_oper._* is the number of operations in a one-time measurement, *T_nor_* and *T_DeC_* are the time for signal (at each frequency) normalization and decoding, respectively. *T_BGS-D_* is the time for demodulating all sampled BGSs along the fiber by the curve fitting or other BGS demodulation methods. It is worth noting that **1)** the *T_RT_* is directly proportional to the sensing distance *L_s_*, **2)** the *T_avg_*, *T_nor_*, *T_DeC_*, and *T_BGS-D_* are all proportional to data volume that is determined by the *T_RT_* (∝ sensing distance) and sample rate (∝ spatial resolution), **3)** longer sensing distance brings higher signal attenuation and increased *N_avg_*. Accordingly, **it is harder to achieve dynamic measurement under a longer sensing range and/or higher spatial resolution**.

From **Eqs. S5 to S9**, the measurement speed (=1/*T_M_*) of conventional high-spatial-resolution B-DFOS is severely slowed down by **1)** multiple-point spatial scanning^24, 25, 30^ for fine spatial information reconstruction, **2)** multiple-point frequency scanning for BGS reconstruction^12, 20-31^ and **3)** large number of signal averaging for SNR improvement. Meanwhile, the signal post-processing speed (1/*T_P_*, i.e., interrogation speed) is severely limited by **Ⅰ)** BGS demodulation based on curve fitting, and **Ⅱ)** the spatial and frequency scanning processes introduced additional time for signal normalization and decoding. Altogether, as the **conventional B-DFOS requires too many operations to complete** **the one-time measurement, the response speed of previous high-spatial-resolution B-DFOS is quite low, which limits them to static measurements**.

On the contrary, **TABS can complete the single measurement with** **much fewer** **operations** **(*N_oper._*)**, since **1)** a two-point frequency scanning (*N_SS_*=1, *N_FS_* =2) and **2)** a markedly reduced trace averaging number (*N_avg_*), are only needed. Accordingly, the measurement time (*T_meas._*) of TABS becomes:

$T_{M}=2\cdot\left[T_{FS}+N_{avg}\cdot\left(T_{RT}+T_{avg}\right)+T_{r\mathrm{e}c}\right]\approx2\cdot N_{avg}\cdot\left(T_{RT}+T_{avg}\right)$ (S10)

It can be observed that the measurement time of TABS is significantly reduced and mainly determined by the signal averaging time (*N_avg_*·*T_avg_*) and fiber length (*N_avg_*·*T_RT_*). Minimizing the signal averaging time (*N_avg_* and *T_avg_*) is promising to further enhance the measurement speed. Meanwhile, in the signal post-processing phase, instead of the time-consuming BGS demodulation procedure, **TABS can extract the BFS distribution after the simple BGR calculation** including pointwise division, logarithm, and calculation of signal real part, as shown in **Eqs. S6 and S7**. The time for the BGR calculation is negligible. The signal post-processing time (*T_PP_*) of TABS is:

$T_{P}=2\cdot\left(T_{nor}+T_{DeC}+T_{BGR-C}\right)$ (S11)

where *T_BGR-C_* denotes the time for calculating the BGR values along the fiber (*T_BGR-C_*≪*T_BGS-D_*). From **Tab. S2** and **Eqs. S10 and S11**, it can be deduced that, under the same signal generation (e.g., waveform generator and microwave generator) and acquisition (e.g., analog-to-digital converter) devices (i.e., the same *T_SS_*, *T_FS_*, *T_avg_* (∝data volume)), **the response speed (1/*T_R_*) of TABS is 1 to 2 orders of magnitude higher than the conventional high-spatial-resolution B-DFOS** even though a longer distance and comparable spatial resolution are achieved simultaneously.

# **Supplementary Section 7: Analyses of the measurement accuracy of TABS**


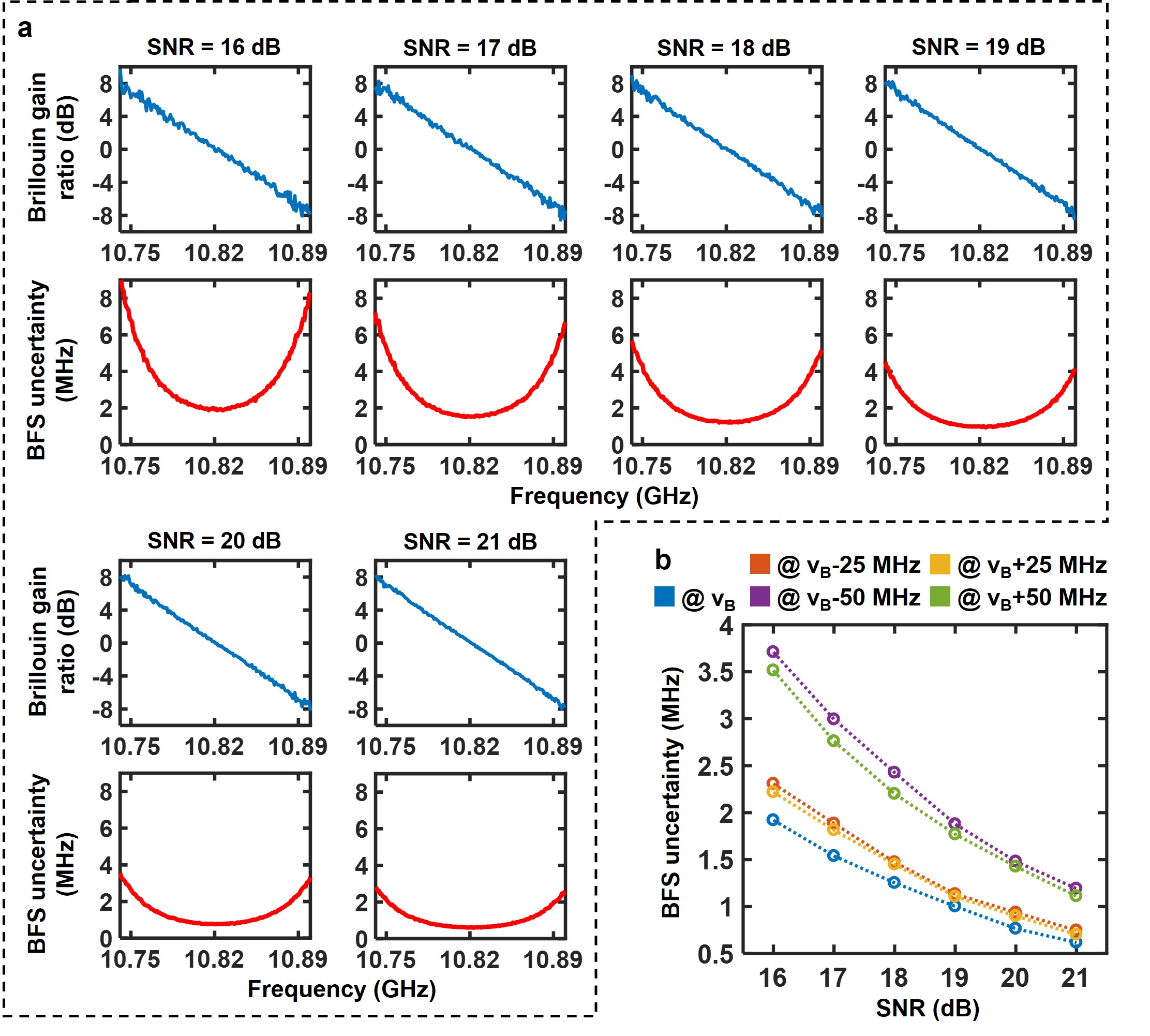


**Fig. S8**: **Theoretical analyses of measurement uncertainty under different SNRs. a,** The BGRSs and measurement uncertainties under different frequency offsets and different SNRs. **b,** Details of the relationship between the SNR and measurement uncertainty. v_B_: BFS.

To investigate the measurement accuracy of TABS, theoretical simulations, and experimental demonstrations are carried out. The measurement uncertainty under different SNR and pump-probe frequency offset is determined by calculating standard deviation (SD) values of 2000 BGRS with distinct random noises. The basic simulation parameters such as intrinsic Brillouin linewidth, pulse width, and so forth, are the same as that in the former simulation as described in **Supplementary Section 5**. The simulation results are shown in **Fig. S8**. From **Fig. S8a**, it can be observed that the measurement uncertainty increases when local Brillouin resonant frequency (i.e., BFS) shifts away from the pump-probe frequency offset. Meanwhile, it can be observed that the measurement accuracy is directly proportional to the SNR with an appropriately linear relationship, as illustrated in **Figs. S8a and S8b**. The above results indicate that to reach a higher accuracy under a wider measurement range, the SNR should be sufficiently high. Fortunately, TABS is highly robust to all detrimental effects parasitized in the pump-probe SBS process, ensuring a high SNR along the whole sensing range, as shown in **Fig. S5h** in **Supplementary Section 4**.

Furthermore, **a theoretical simulation is designed under the experimental conditions** to compare the measurement accuracies between theory and experiment. Specifically, the BFS distribution measured in the experiment is used as the BFS distribution in the simulation. Under the given BFS distribution, the BGS distribution can be calculated. Then, random noise is added to the BGS distribution. The noise power at different locations is calculated according to the peak SNR distribution under the BFS condition (shown in **Fig. S5h**). After the theoretical BGS distribution is constructed, the BGRS can be calculated by **Eq. S3**. The BFS distribution under the noise is then extracted by the BGR method (**Eq. S4**), as illustrated in **Fig. S9a** (BGR-sim.). It can be observed that compared with the original BFS distribution, the extracted BFS distribution is more fluctuated due to the random noises.

To analyze the measurement accuracy quantitatively, the measurement uncertainty is calculated by the moving SD values of the difference between original and extracted BFS distributions. Different from the traditional way that determines the measurement uncertainty by calculating the SD value of the BFS distributions from multiple measurements, the moving SD is adopted here for calculating the measurement uncertainty in both simulation and experiment, since **1)** the data volume of complete BGS distribution under long-range and high-spatial-resolution is so high that the time for calculating or measuring even only one complete BGS distribution is very long; **2)** The SNR of BGS in TABS is sufficiently high, and the BGS distribution extracted by linear fitting method is sufficiently accurate and can be used as the reference signal; **3)** The window size of moving SD is 100 points, corresponding to 20 m in length. The Brillouin signal power within such a short window is nearly the same. In this way, the accuracy and statistical properties of BFS uncertainty calculation can both be ensured.


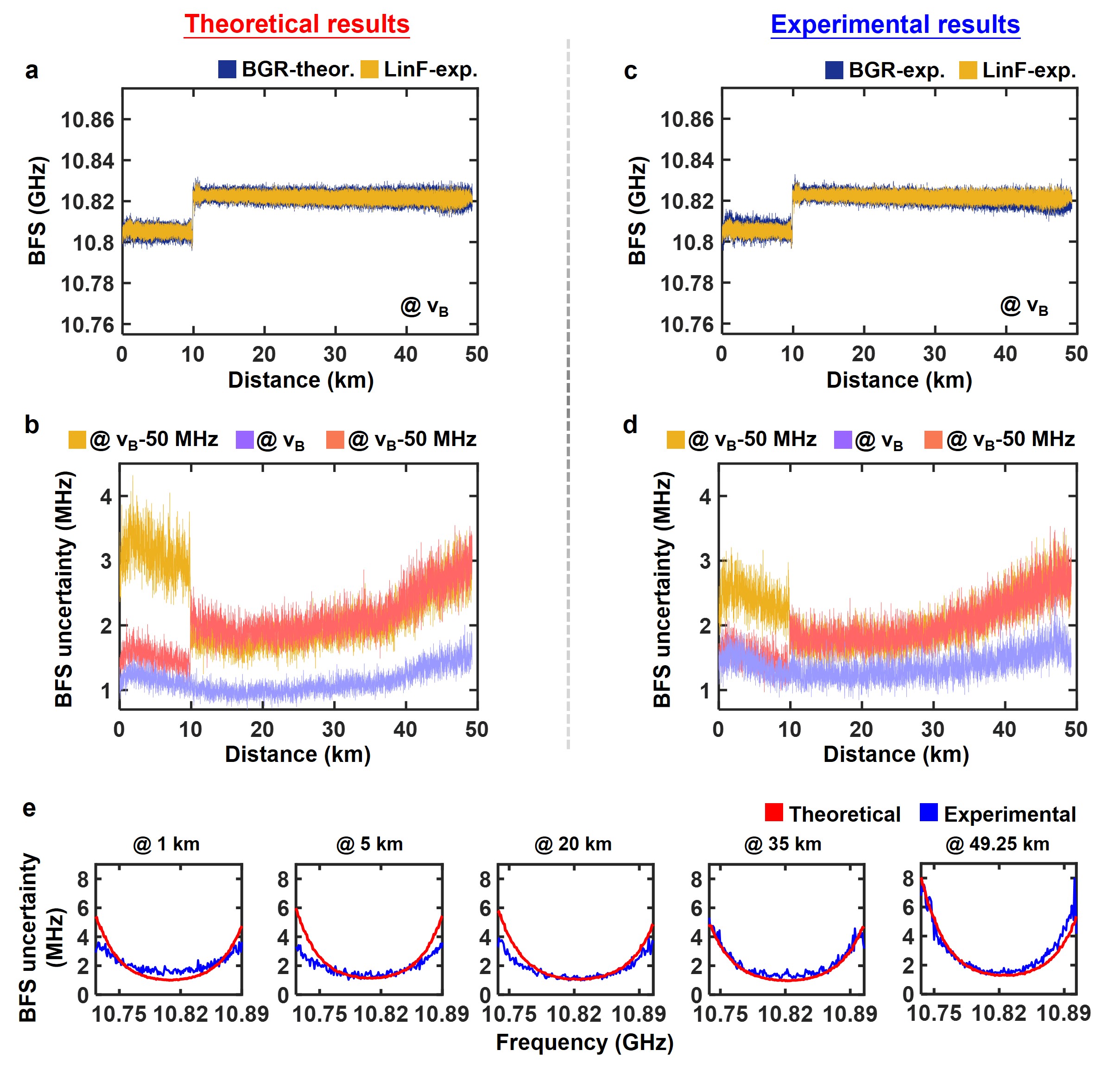


**Fig. S9: Theoretical and experimental results of measurement uncertainty along the fiber.** **a, c,** BFS distribution in (a) simulation and (c) experiment. BGR-theor. (or exp.): theoretical (or experimental) BFS distribution extracted by using the BGR method. LinF.-exp.: experimental BFS distribution extracted by using linear fitting method. **b, d,** BFS uncertainty along the fiber in (b) simulation and (d) experiment. ν_B_: BFS. **e,** Measurement uncertainty as a function of pump-probe frequency offset at different fiber locations in the simulation and experiment.

The measurement uncertainty in simulation is shown in **Fig. S9b**. Matching well with the theoretical results shown in **Fig. S8**, **the measurement uncertainty is highly related to the pump-probe detuning frequency and SNR**. The measurement uncertainty is high in the first 10 km due to the BFS difference between the first (~10 km long) and second (~40 km long) part of fiber. Meanwhile, the BFS distribution and corresponding measurement uncertainty (calculated by the moving SD) in the experiment are also shown in **Figs. S9c and S9d**, respectively. The measurement uncertainty in the experiment has been illustrated in **Fig. 2i** in the main text, it is shown again here for comparison.

**Interestingly, the measurement results (in Fig. S9d) in the experiment show a better certainty in the former part of the sensing fiber than that in the simulation (in Fig. S9b).** Further, the measurement uncertainties under different frequency offsets at different fiber locations are calculated and shown in **Fig. S9e**. It is now clear that when the fiber BFS shifts away from the pump-probe frequency offset, **1)** in the former part of the sensing fiber, the measurement uncertainty increment rate in the experiment is markedly lower than that in the simulation, **2)** in the later part of the sensing fiber, the measurement uncertainty increment rate in the experiment is nearly the same as that in the simulation.

**The *origin* of this interesting phenomenon is that the optical noise is Brillouin-gain-dependent**. As described in **Supplementary Section 4**, higher Brillouin gain brings stronger optical noise (especially polarization noise). In the simulation, the power of random noises for all pump-probe frequency offsets is determined solely by the peak SNR calculated by using the Brillouin signal at the BFS. While the real noise power evolution in the experiment is **1)** when the pump-probe frequency offset is aligned with the fiber BFS, the Brillouin gain and optical noise are strongest; **2)** when the fiber BFS shifts away from the pump-probe frequency offset, the Brillouin gain and optical noise becomes weaker. Therefore, in the former part of the fiber where the optical noise is dominated, the noise shows a non-uniform distribution across the whole frequency range. The noise (mainly optical noise) becomes weaker and weaker when the pump-probe frequency offset is shifted away from the BFS, which brings a lower measurement uncertainty increment rate. In the later part of the fiber, the Brillouin gain becomes lower and the noise is dominated by amplified spontaneous emission (ASE) noise in the EDFA and electrical noises in the PD (such as shot noise, thermal noise, and so forth) which are independent to the pump-probe frequency offset. In this situation, the noise distribution is uniform across the whole frequency range. As a result, the measurement uncertainty in the experiment is the same as that in the simulation.

Collectively, the high robustness against the detrimental effect enables TABS to have a high SNR and measurement accuracy along the whole sensing range.

# **Supplementary Section 8: Analyses of the spatial resolution of TABS**


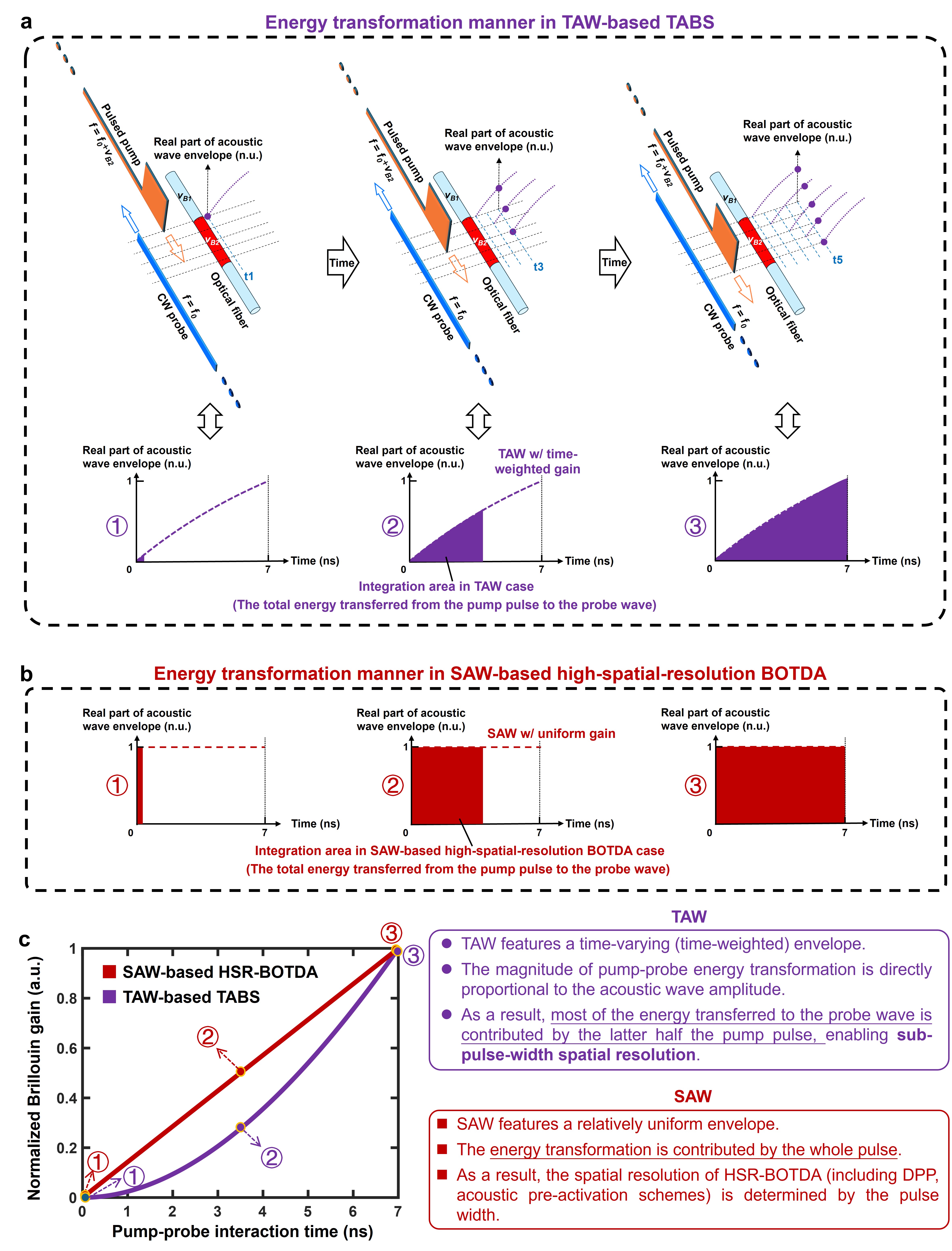


**Fig. S10: Energy transformation manners and spatial resolutions of different B-DFOS. a, b,** Energy transformation manners in (a) TAW-based TABS sensor and (b) SAW-based high-spatial-resolution (HSR) BOTDA sensor. **c,** the edges of Brillouin signals at the BFS in cases of TAW and HSR-BOTDA. Each Brillouin gain point in (c) is the integration result in each time period in (a) and (b).

**Figure S10a** illustrates the energy transformation manner in the TAW-based TABS sensor. TAW features a time-varying gain evolution: with the increase of pump-probe interaction time, the strength of TAW increases accordingly. This time-weighted gain property determines a non-uniform pump-probe energy transformation: **most of the energy transferred to the probe wave is contributed by the latter half of the pump pulse** where the pump-probe interaction has been sustained for a sufficiently long time, and the acoustic wave has become relatively strong. For instance, when the pump pulse starts to collide with the probe wave (t1 time), the acoustic wave is so weak that the pump-probe energy transformation is tiny and the Brillouin gain added on the probe wave is ignorable, as illustrated in **Fig. S10a (left)**. After the probe wave has interacted with half of the pump pulse, the acoustic wave and Brillouin gain becomes relative strong, as shown in **Fig. S10a (center)**. Finally, after the probe wave interacts with the whole pump pulse, the strength of acoustic wave reaches its peak and the Brillouin gain is most significant, as illustrated in **Fig. S10a (right)**. With the time-weighted gain property, the Brillouin signal (i.e., Brillouin gain) carried by the probe wave exhibits a growth trend resembling an exponential increase, as shown in **Fig. S10c**, which shortens the signal edge to a sub-pulse-width length and enables sub-pulse-width spatial resolution.

Meanwhile, in the conventional high-spatial-resolution (HSR) BOTDA (including DPP, acoustic wave pre-activation methods^20^), the acoustic wave is pre-activated to the steady regime (i.e, SAW) which features a uniform temporal envelope. The resulting Brillouin energy transferred to the probe wave is contributed by the whole pump pulse uniformly, as illustrated in **Fig. S10b**. Consequently, the Brillouin signal carried by the probe wave exhibits a linear trend, as illustrated in **Fig. 10c**, and the spatial resolution is determined by the pulse width.

In the following part, the spatial resolution of TAW-based TABS sensor is validated theoretically and experimental.


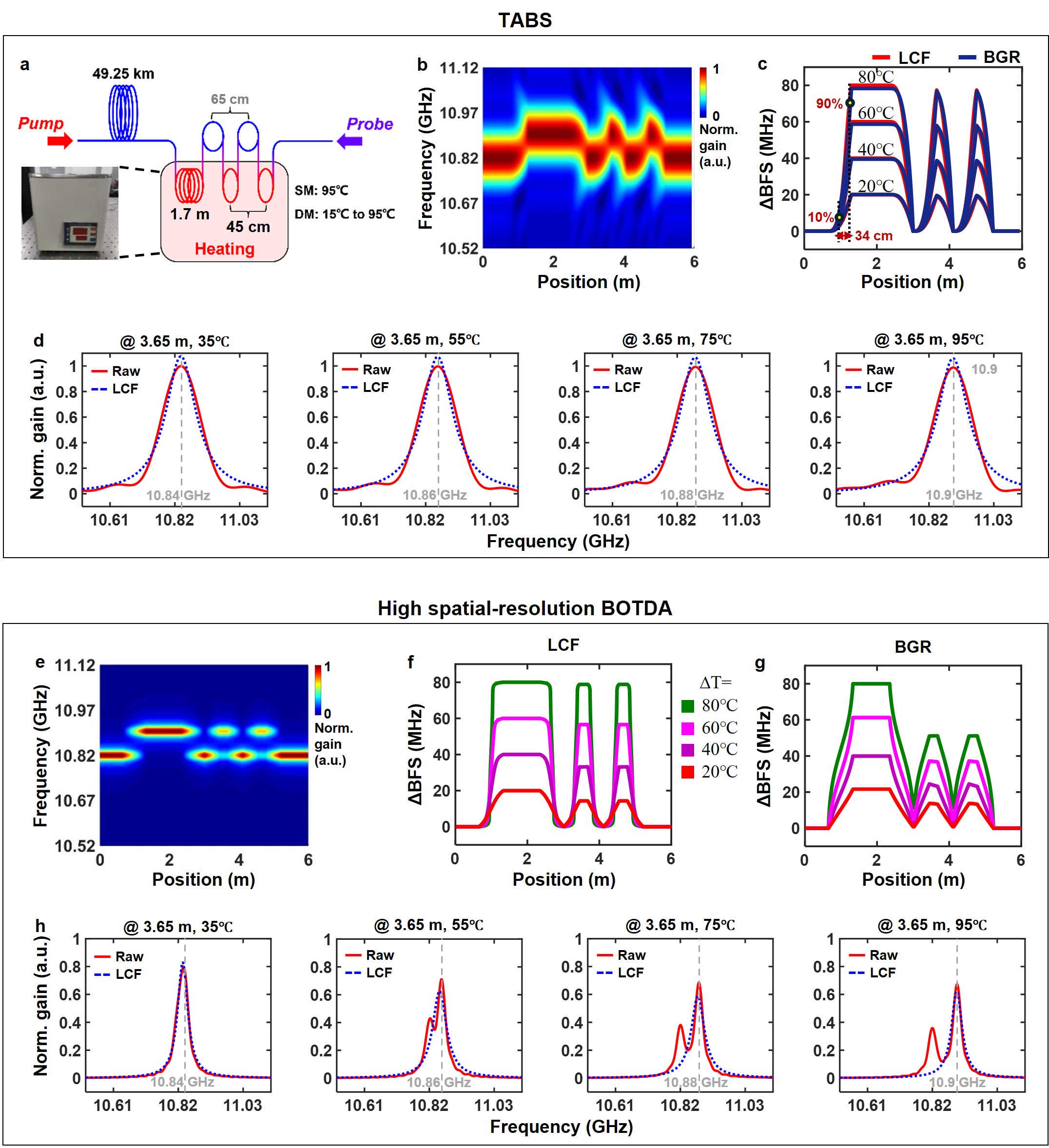


**Fig. S11: Theoretical analyses of the spatial resolution of TABS and conventional high-spatial-resolution B-DFOS. a,** Test configuration in both simulation and experiment. SM: static measurement. DM: dynamic measurement. **b, c,** Theoretical (b) BGS and (c) BFS distributions in TABS. The BFS is extracted by using both Lorentz curve fitting (LCF) and BGR methods for comparison. Norm.: normalized. **d,** The BGSs under different hot spot temperatures at 3.65 m in TABS. **e,** Theoretical BGS in conventional high-spatial-resolution DPP-BOTDA. **f, g,** The BFS distributions extracted by using the (f) LCF and (g) BGR, respectively. **h,** The BGSs under different hot spot temperatures at 3.65 m in the DPP-BOTDA.

To validate the spatial resolution of TABS, theoretical simulations, and experimental demonstrations are implemented. The theoretical simulations are based on the SBS coupled-three-wave equation^10-14^. Here, the test configuration for both simulation and experiment is shown in **Fig. S11a**. Three fiber sections are heated from 15℃ to 95℃, which results in an 80-MHz BFS variation. The lengths of the first, second, and third heating fiber sections are 1.7 m, 45 cm, and 45 cm, respectively. The spacing between adjacent heating fiber sections is 65 cm. In the simulation, the results are calculated in the range from 0 to 6 m with a step size of 1 cm. The pulse width of TABS is 6.8 ns which is the same as that in the experiment and theoretical analysis in **Supplemental Note 2**. The BFS and Brillouin linewidth are set as 10.82 GHz and 30 MHz, respectively. The pump-probe frequency offset is scanned from 10.52 GHz to 11.12 GHz (i.e., frequency range = 600 MHz) with a 1 MHz step. Notably, the BFS is extracted by both the conventional Lorentz curve fitting (LCF)^15, 63^ and the BGR methods to observe the signal evolution at different locations more comprehensively.

**Figures S11b and S11c** show the calculated BGS distribution and extracted BFS distributions, respectively. In the BGR method, the Δ*f_DS_* is 80 MHz which is the same as that in the experiment. **The LCF- and BGR-extracted BFS distributions are almost aligned, indicating that the BGR under the TAW can extract the BFS precisely**. From 10% to 90% of the rising edge, the **theoretical spatial resolution is determined to be 34 cm**^23^ which matches well with the 37 cm spatial resolution measured in the experiment. The difference between theoretical and experimental spatial resolutions stems from that the pump pulse in the experiment is not an ideal rectangular pulse. The raising and falling edges of the pump pulse introduce additional energy superposition, which leads to the degradation of experimental spatial resolution. The theoretically calculated and experimentally measured spatial resolution indicates that when the width of the hot spot is larger than 34 cm, over 90% of the true temperature or strain variation can be acquired. The inference can be validated by measuring the BFS variations of the second and third hot spots with a width of 45 cm. In the simulation, the peak BFS variations of the second the third hot spots are both 76.77 MHz which is ~96% of the true BFS variation (80 MHz). In the experiment, the peak BFS variations of the second the third hot spots are 79.5 MHz (99.38% of the true BFS variation (80 MHz)) and 76.6 MHz (~96% of the true BFS variation (80 MHz)), respectively, as shown in **Fig. 2k** in the main text.

Further, to investigate the BFS change and spatial resolution in other situations, the BGS distributions are calculated under the heat source’s temperatures of 35℃, 55℃, and 75℃ which correspond to the BFS variations of 20 MHz, 40 MHz, and 60 MHz, respectively. The extracted BFS variations are also shown in **Fig. S11c**. It can be observed that for all situations, the raising edge widths are the same. The peak BFS variations at the second (or third) hot spot under 20 MHz (i.e., 35℃), 40 MHz (i.e., 55℃), and 60 MHz (i.e., 75℃) theoretical BFS variations are 19.6 MHz, 38.76 MHz, and 57.68 MHz, respectively. The extracted BFS changes are 98%, 97%, and 96% of the theoretical BFS change of 20 MHz, 40 MHz, and 60 MHz, respectively. Moreover, **Fig. S11d** shows the raw BGSs at the second hot spot location (3.65 m) under 35℃, 55℃, 75℃, and 95℃. It can be observed that for each temperature, there is only one Brillouin gain peak with a gain value larger than 0.988 (the Brillouin gain is normalized to 1). From the above theoretical and experimental analyses, **a 34 cm spatial resolution is achieved under a 6.8 ns wide pump pulse, demonstrating the sub-pulse-width spatial resolution endowed by the time-weighted gain property of TAW.**

Moreover, to compare the spatial resolution with the same pulse width under the SAW, the DPP-BOTDA (a prevailing method for reaching high spatial resolution) is also simulated for comparison. The test configuration is the same as that in **Fig. S11a**. The long and short pulses’ widths are 40 ns and 33.2 ns (differential pulse width = 6.8 ns), respectively. Similarly, the hot spots’ temperatures are set as 35℃, 55℃, 75℃ and 95℃ for comparison. **Figure S11e** shows the calculated BGS distribution under the hot spots’ temperature of 95℃ (room temperature=15℃, theoretical BFS variation=80 MHz). It can be found that, in contrast to the TABS case where the BGS features only one Brillouin gain peak at each hot spot position, **the BGSs at the second and third hot spots’ locations in the DPP-BOTDA case have two Brillouin gain peaks**, due to insufficient spatial resolution (68 cm, determined by the differential pulse width).

Then, the BFS distributions under different hot spots’ temperatures are extracted by using the LCF and BGR methods, as shown in **Figs. S11f and S11g**, respectively. Here, the BGR’s Δ*f_DS_* is set as 100 MHz for a wider measurement range. It can be found that, due to the dual Brillouin gain peaks of the BGS, the BFS distributions extracted by the LCF and BGS are different. On the one side, in the LCF case, the BFS variations of the second (or third) hot spots under 35℃, 55℃, 75℃, and 95℃ are 14.29 MHz, 33.16 MHz, 56.54 MHz, and 78.82 MHz, respectively. The extracted BFS changes are 71.45%, 82.9%, 94.23%, and 98.52% of the theoretical BFS change of 20 MHz, 40 MHz, 60 MHz, and 80 MHz, respectively. On the other side, in the BGR case, the second (or third) hot spots’ BFS variations are extracted to be 13.27 MHz, 23.24 MHz, 36.71 MHz, and 51.11 MHz, respectively, which are 68.6%, 58.1%, 61.2% and 63.9% of the theoretical BFS change of 20 MHz, 40 MHz, 60 MHz, and 80 MHz, respectively.

Clearly, **in the DPP-BOTDA, the LCF- and BGR-extracted BFSs show different evolutions** under different temperature changes. The source of this phenomenon is that

**1) the LCF bases on the feature of BGS.** As shown in **Fig. S11h**, the dual-Brillouin-gain-peaks characteristic determines that the LCF will choose the strongest Brillouin gain peak when the dual Brillouin gain peaks are sufficiently separated from each other. This kind of gain peak selection manner of LCF can work only when the Brillouin gain peak at the changed BFS (due to the increments of temperature or strain) is stronger than the one at the fiber average BFS. However, when the width of hot spot is significantly shorter than the spatial resolution, the Brillouin gain peak at the changed BFS is weaker than the one at the fiber normal BFS. In this situation, the LCF will only fit the stronger Brillouin gain peak at the fiber normal BFS and miss the real signal from the hot spot (this point will be discussed below).

**2) The BGR method bases on the BGR value only.** The insufficient spatial resolution induced BFS deviation has almost the same ratio (~60%) for all temperatures, which matches well with the ratio between the hot spot’s length (45 cm) and differential pulse width (68cm), and demonstrates that the spatial resolution of DPP-BOTDA is indeed 68 cm. Refined from the above results, under the same pulse width, TABS offers higher precision and reliability than the conv. high-spatial-resolution BOTDA due to improved spatial resolution.

In practical applications, strain or temperature variations may occur at very short fiber sections. The capability to measure short hot spots is important. Here, we further investigate the **extreme sensing capability** for short hot spots. **Figure S12a** shows the test configuration in both simulation and experiment. Four heat sources with lengths of 20 cm, 30 cm, 40 cm, and 1.5 m are set. The temperature of all hot spots is 95℃ which corresponds to 80 MHz theoretical BFS change.

**
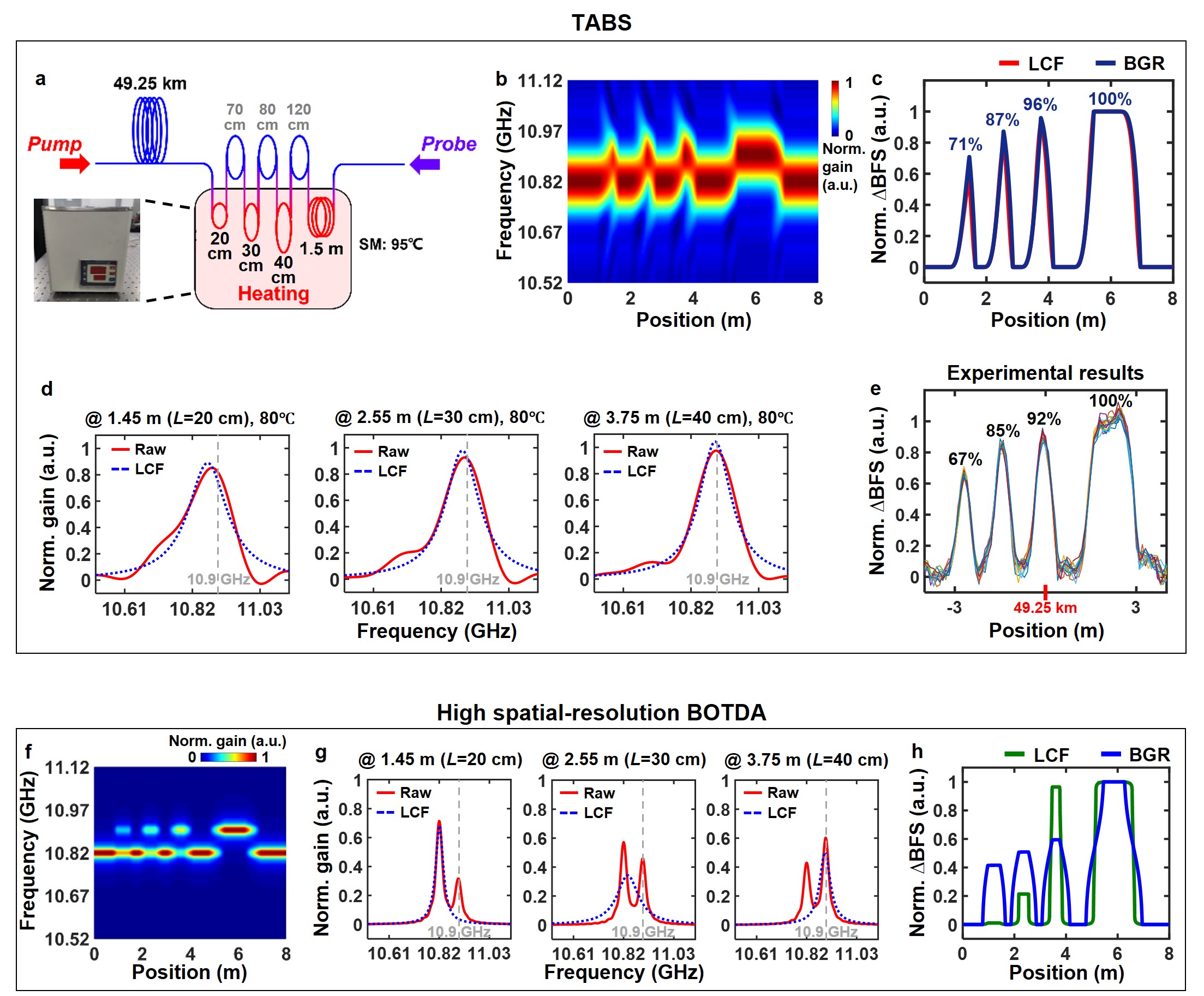
**

**Fig. S12: Theoretical and experimental analyses of extreme sensing capability of TABS. a,** Test configuration in both simulation and experiment. **b, c,** Theoretical (b) BGS and (c) BFS distributions in TABS. **d,** BGSs under 80℃ at 1.45 m, 2.55 m and 3.75 m in TABS. **e,** Experimental result of BFS distributions in TABS. The Brillouin signals in 15-times measurements are here overlapped together for verifying the repeatability. **f,** The BGS distribution in conventional high-spatial-resolution DPP-BOTDA. **g,** The BGSs under 80℃ at 1.45 m, 2.55 m and 3.75 m in DPP-BOTDA. **h,** The BFS distributions extracted by LCF and BGR.

**Figure S12b** shows the calculated BGS distribution in TABS. **Figure S12c** illustrates the BFS distribution extracted by the LCF and BGR methods. The BFS variation is normalized to 1 for visual clarity. **Figure S12d** shows the measured BGS at first (at 1.45 m), second (at 2.55 m), and third (at 3.75 m) hot spots. It can be observed that the BFS variation under 20 cm, 30 cm, and 40 cm are 71%, 87%, and 96% of peak BFS variation at the fourth hot spot with a length of 1.5 m. This means that, **even though the hot spot is as short as 20 cm, TABS can still acquire 71% of the true measurand’s information**. On this basis, an experiment is carried out to verify the sensor capability. The test configuration in the experiment is the same as that in the simulation and shown in **Fig. S12a**. The BFS distribution is extracted by the BGR method. The experimental result is shown in **Fig. S12e**. To verify the repeatability, the BFS distribution is consecutively measured by 15 times. The resulting 15 BFS distributions are shown in **Fig. S12e**. It can be found that the BFS variations at the first, second, and third hot spots are 67%, 85%, and 92%, respectively. **The experimental result matches well with the theoretical analyses, demonstrating the capability to sense small-size targets**

In addition, the BGS and BFS distributions in the case of conventional high-spatial-resolution DPP-BOTDA are also calculated, as illustrated in **Figs. S12f to S12h**. It can be found that **1)** when the LCF method is adopted, the BFS variations at the first (20 cm long) and second (30 cm long) hot spots are quite small and barely perceptible since the Brillouin gain peak at the hot spot is so weak to be fitted by the LCF; **2)** when the BGR is used, although the BFS variations at the first (20 cm long) and second (30 cm long) hot spots are larger than that in the LCF, they are only 41% and 51% of the theoretical BFS variation. Refined from the above analyses, **owning to the time-weighted gain property of TAW, TABS feature not only a sub-pulse-width spatial resolution (34 cm) but also a good adaptability to extreme sensing conditions.**

# **Supplementary Section 9: Analyses of sensing performance of TABS under other conditions**


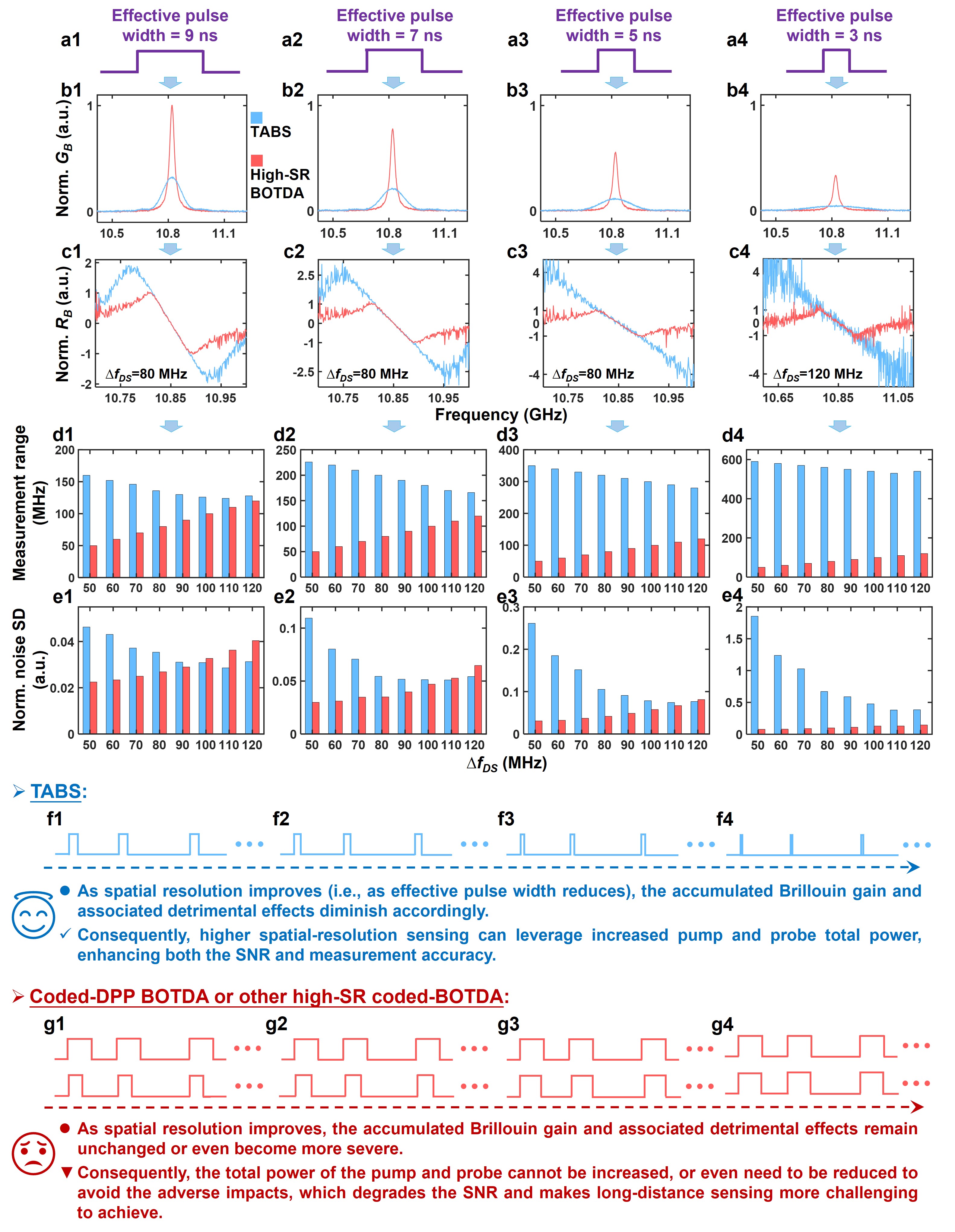


**Fig. S13: Theoretical analyses of the measurement range and accuracy of TABS under different effective pulse widths. a,** Schematic diagram of effective pulse width. **b, c,** The (b1)-(b4) Normalized BGS (G_B_: Brillouin gain) and (c1)-(c4) BGR (R_B_) under different effective pulse widths. Norm.: normalized. **d, e,** The (d1)-(d4) Measurement ranges and (e1)-(e4) normalized noise standard deviation (SD) values under different effective pulse widths and dual-slope frequency spacings. **f, g,** Schematic diagram of optical pulse trains in (f1)-(f4) TABS and (g1)–(g4) coded-DPP BOTDA or other high-SR coded-BOTDA.

The above theoretical and experimental results have demonstrated improvements in robustness against detrimental effects, measurement accuracy, response speed (temporal resolution), and spatial resolution. The theoretical results are in good agreement with the experimental results, demonstrating the high precision of theoretical simulation. In the following part, the theoretical analyses are carried out to further explore the sensing performance of TABS under other conditions.

Firstly, the measurement accuracy under different effective pulse widths and ∆*f_DS_* are investigated, as illustrated in **Fig. S13a**. The effective pulse width stands for the actual time for Brillouin gain accumulation. In DPP- and PSP-BOTDA, the effective pulse width equals the pulse width difference between two differential pulse width pairs^21, 22^. While the effective pulse width in TABS equals to the pump pulse width. Random noise is added to the BGSs of the SAW and TAW under all effective pulse widths, as shown in **Fig. S13b**. Under the random noise, the peak SNR (at the BFS) of TAW-based BGS (effective pulse width=7 ns) is ~18 dB. It is worth noting that, in the current simulation, same random noise is added on both SAW- and TAW-based BGSs for simplicity of comparison. However, as analyzed in **Supplementary Section 4** and summarized in **Tab. S1**, the BOTDA (based on the SAW) is susceptible to multiple detrimental effects due to high Ac. G_B_. Therefore, the actual noise in the SAW-based BGS in the BOTDA would be much higher than that in the TAW-based BGS in TABS. The BGSs under different effective pulse widths in **Fig. S13b** are normalized according to the peak gain of SAW-based BGS. **Figure S13c** shows the BGRSs under different effective pulse widths. The ∆*f_DS_* in **Figs. S13c1 to S13c4** are 80 MHz, 80 MHz, 80 MHz, and 120 MHz, respectively. Since the original BGRSs in SAW and TAW have different slopes, the BGRSs of TAW in **Figs. S13c to S13e** have been multiplied by a coefficient *k* to make the BGRSs in both SAW and TAW have the same slope for comparison and visual clarity. Notably, since the signal and noise are both multiplied by *k* times, the SNRs before and after the multiplication operation are the same. The normalized BGRS in TAW and the coefficient *k* are given by:

$Nor.\text{ }R_{B-MSAW}^{n}(f)=R_{B-MSAW}^{n}(f)*k$ (S12)

$k=\frac{Max\left[R_{B-SAW}^{0}(f)\right]}{R_{B-MSAW}^{0}(f_{SAW}^{\max})}\cdot c$ (S13)

where $R_{B-MSAW}^{n}$ is the original BGRS with random noise in the TAW. $Nor.\text{ }R_{B-MSAW}^{n}(f)$ is the normalized BGRS with random noise in the TAW. $R_{B-SAW}^{0}(f)$ is the BGRS without noise in the SAW case. $f_{SAW}^{\max}$ is the frequency point corresponding to the maximum of $R_{B-SAW}^{0}(f)$. *c* is a variable coefficient (equal to 1 to 1.2 in general) to fine-tune the slopes between the BGRSs in cases of SAW and TAW. From **Fig. S13c**, it can be found that when the effective pulse width is larger than 5 ns, **the BGRS in the TAW features a significantly improved linear range than that in the SAW**, while the noise is nearly the same.

Furthermore, the measurement range and accuracy in the SAW and TAW cases under different effective pulse widths and ∆*f_DS_* are evaluated and shown in **Figs. S13d and S13e**, respectively. Here, the measurement accuracy is evaluated by calculating the SD value of the noise on the normalized BGR at the BFS. The higher Nor. noise SD indicates a lower measurement accuracy. From **Figs. S13d1 to S13d4 and S13e1 to S13e4**, it can be found that even though the noise in the BGS of SAW is assumed to be the same as that in the TAW (i.e., all detrimental effects parasitized in the BOTDA are ignored in this assumption), the TAW’s BGRS still features comparable measurement accuracy and 2-3 times enhanced measurement range than that of the SAW. Meanwhile, it can also be found that the measurement accuracy degrades rapidly when the effective pulse width is 3 ns due to insufficient signal power.

Fortunately, as analyzed in **Supplementary Section 8** and illustrated in **Fig. S13f**, with the decrease of effective pulse width (i.e., the improvement of spatial resolution), the pulse width in TABS decreases accordingly. As a result, the Ac. G_B_ and detrimental effects alleviate significantly, which allows to employ higher probe input power and longer coded pump pulse sequences to further suppress the noise and enhance the SNR. This indicates that **there is a wider room for SNR improvements for lower effective pulse width (or equivalently, higher spatial resolution) in TABS**.

**
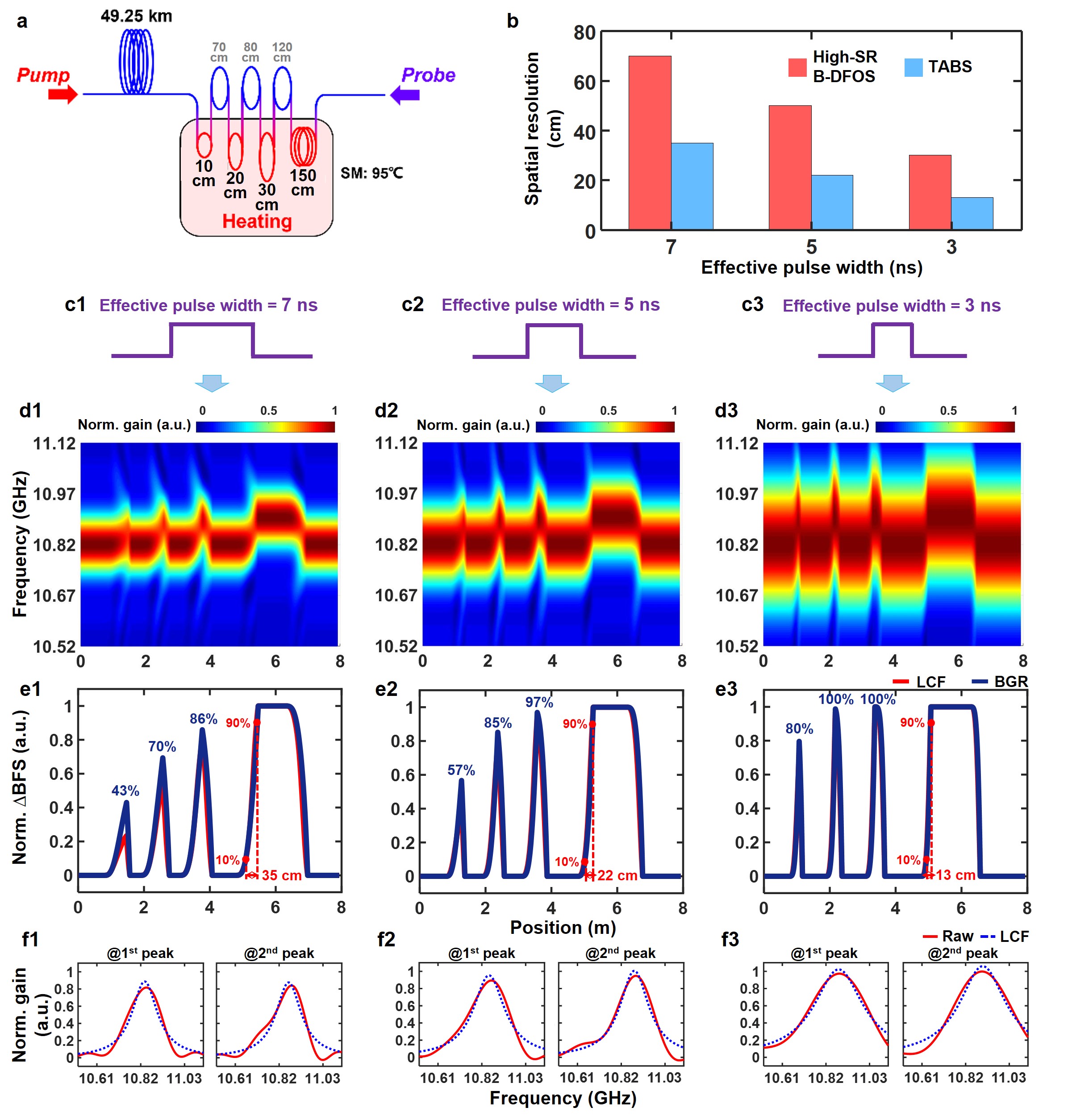
**

**Fig. S14: Theoretical analyses of the spatial resolution of TABS under different effective pulse widths. a,** Schematic diagram of simulation configuration. SM: static measurement. **b,** Theoretical spatial resolution under different effective pulse widths. SR: spatial resolution. **c,** Schematic diagrams of effective pulse width. **d,** BGS distributions, **e,** BFS distributions and **f,** BGSs under different effective pulse widths.

On the contrary, in the conventional high-spatial-resolution BOTDA, the use of long optical pulse is inevitable due to the requirement of steady acoustic wave stimulation, as illustrated in **Fig. S13g**. As a result, no matter how short the effective pulse width (i.e., the pulse width difference in the DPP- and PSP-BOTDA), the optical pulse trains constituted by long optical pulses will always give rise to high Ac. G_B_ and severe detrimental effects, as illustrated in **Figs. S4 and S5**. Consequently, it is getting harder and harder to improve the SNR with the decrease of effective pulse width (or equivalently, higher spatial resolution).

Apart from the measurement range, the spatial resolutions in TABS under other effective pulse widths are also investigated, as shown in **Fig. S14**. **Figure S14a** illustrates the simulation configuration which is similar to that in **Fig. S12a**. The first, second, and third hot spots’ lengths are 10 cm, 20 cm, and 30 cm, respectively. **Figure S14b** shows the theoretical spatial resolutions under the effective pulse widths of 7 ns, 5 ns, and 3 ns. **Figures S14c to S14f** show the detailed theoretical calculation results. It can be observed that the theoretical spatial resolutions of TABS are 35 cm, 22 cm, and 13 cm when the effective pulse widths are 70 cm, 50 cm, and 30 cm, respectively. Accordingly, **for shorter or wider pulse widths, the spatial resolutions of TABS are generally 2-times higher than that of the high-spatial-resolution BOTDA**, thanks to the time-weighted acoustic wave evolution of the TAW. By employing the 3 ns long pump pulse, a 10 cm spatial resolution can be reached.

From the above analyses, it is clear that a larger measurement range and higher spatial resolution can be reached simultaneously by decreasing the pump pulse width. The decrease in pulse width also brings BGS broadening. Theoretical, the wider BGS may need higher SNR to maintain the same measurement accuracy. Here, the required SNR to reach a certainty measurement accuracy is analyzed quantitatively. **Figures S15a1, S15b1, and S15c1** show the BGSs under the pulse widths of 7 ns, 5 ns, and 3 ns, respectively. The corresponding BGRs are shown in **Figs. S15a2, S15b2, and S15c2**. The dual-slope frequency spacings for cases of 7 ns, 5 ns, and 3 ns are 80MHz, 200 MHz, and 470 MHz to obtain the BGRs with the same intrinsic linear region (i.e., intrinsic measurement range) of 200 MHz for comparison. **Figures S15a3, S15b3, and S15c3** show the measurement uncertainties across a 200 MHz measurement range (=100% of the intrinsic measurement range). It can be found that for cases of 7 ns, 5 ns, and 3 ns, the peak SNRs of the BGSs should be higher than 23 dB, 23.4 dB, and 25.8 dB, respectively, to maintain a measurement uncertainty less than 3 MHz. Moreover, the peak SNRs should be higher than 1) 19.7 dB and 16.9


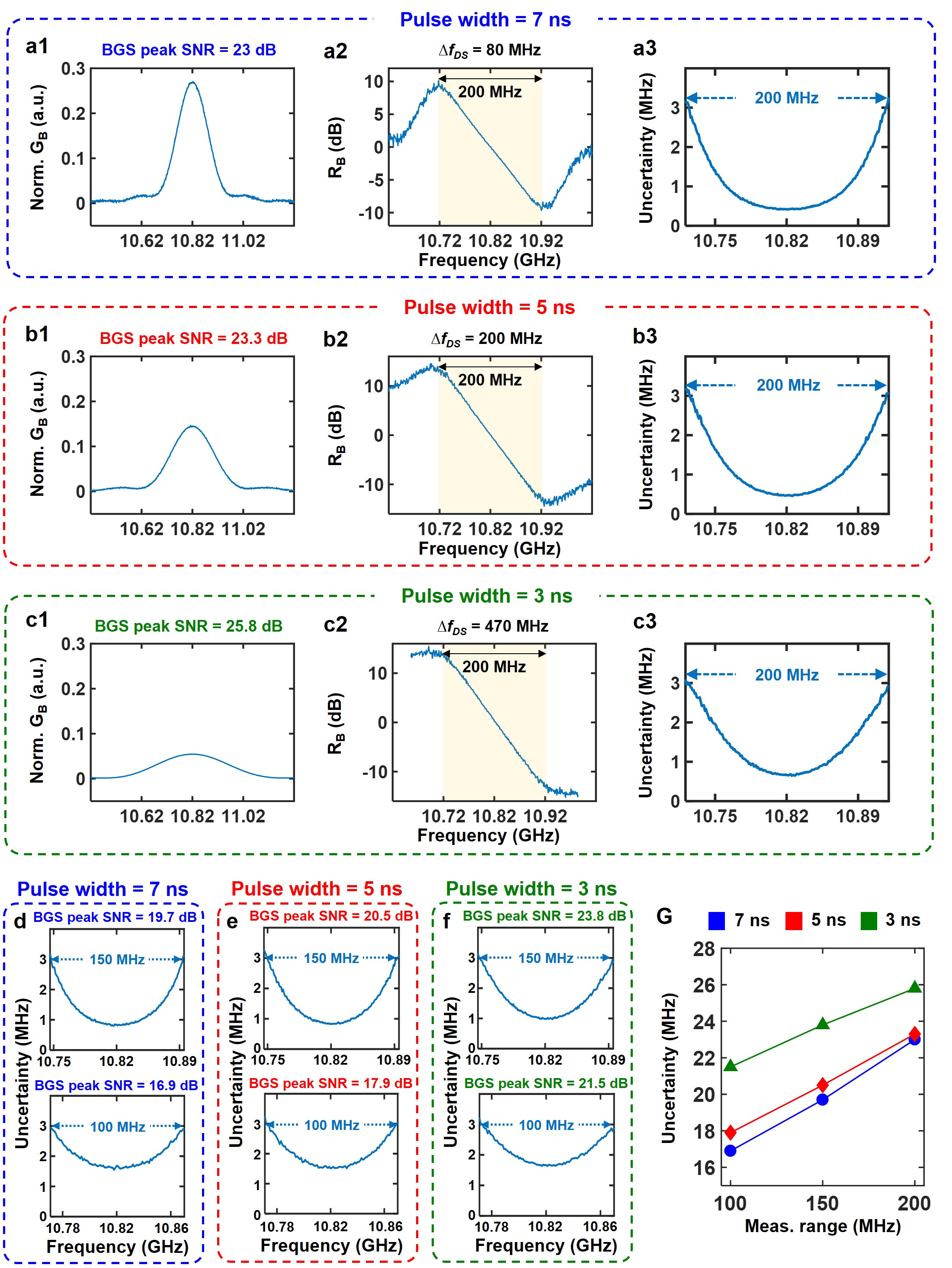


**Fig. S15: Theoretical analyses of the required SNRs to reach the same measurement accuracy under the same intrinsic measurement range and different pulse widths. a1, b1, c1,** The BGSs under different pulse widths (7 ns, 5 ns, and 3 ns) and different peak SNRs (23 dB,23.4 dB, 25.8 dB). **a2, b2, c2,** The BGRs under different pulse widths (7 ns, 5 ns, and 3 ns). The dual-slope frequency spacings (∆*f_DS_*) for cases of 7 ns, 5 ns, and 3 ns are 80 MHz, 200 MHz, and 470 MHz, respectively, which results in the same intrinsic linear range of 200 MHz (corresponding to intrinsic temperature and strain measurement ranges of 200℃ and 4000 με, respectively). **a3, b3, c3,** The measurement uncertainties under different pulse widths (7 ns, 5 ns, and 3 ns). For cases of 7 ns, 5 ns, and 3 ns, the peak SNRs should be higher than 23 dB,23.4 dB, and 25.8 dB to maintain a measurement uncertainty of less than 3MHz across a 200 MHz measurement range. **d,** The measurement uncertainties under the 7 ns pulse width and 200 MHz intrinsic linear range. The peak SNRs should be higher than 19.7 dB and 16.9 dB to maintain a measurement uncertainty of less than 3MHz across 150 MHz and 100 MHz measurement ranges, respectively. **e,** The measurement uncertainties under the 5 ns pulse width and 200 MHz intrinsic measurement range. The peak SNRs should be higher than 20.5 dB and 17.9 dB to maintain a measurement uncertainty of less than 3MHz across 150 MHz and 100 MHz measurement ranges, respectively. **f,** The measurement uncertainties under the 3 ns pulse width and 200 MHz intrinsic measurement range. The peak SNRs should be higher than 23.8 dB and 21.5 dB to maintain a measurement uncertainty of less than 3MHz across150 MHz and 100 MHz measurement ranges, respectively. **g,** The required SNRs to reach a measurement uncertainty of less than 3MHz across 200 MHz, 150 MHz, and 100 MHz measurement ranges under different pulse widths and the same intrinsic measurement range of 200 MHz.

dB (7 ns case in **Fig. S15d**), 2) 20.5 dB and 17.9 dB (5 ns case in **Fig. S15e**), and 3) 23.8 dB and 21.5 dB (3 ns case in **Fig. S15f**), to maintain a measurement uncertainty less than 3MHz across a 150 MHz and 100 MHz measurement ranges (=75% and 50% of the intrinsic measurement range), respectively. The required SNRs for different cases mentioned above are summarized in **Fig. S15g**. **Fig. S15g** is also shown in **Fig. 2d** in the main text**.** It can be observed that the wider BGS shape indeed requires high peak SNRs to maintain the same measurement accuracy, while the difference between the required SNRs for different pulse widths is not high: a ~1 dB difference between the cases of 7 ns and 5 ns, and a ~3 dB difference between the cases of 5 ns and 3 ns. This means that **the impact of BGS broadening on the BGR and corresponding measurement accuracy is small**. However, it must be noted that with the decrease in pulse width, the SBS interaction length is shorter, which leads to lower Brillouin signal intensity. Meanwhile, the higher spatial resolution requires wider detection bandwidth, which may introduce higher electrical noise. Accordingly, with the decrease in pulse width, it becomes harder to reach the required SNR to maintain the target measurement accuracy**. Fortunately, as analyzed above, the TAW-induced BGS broadening also brings increased robustness against the detrimental effects with the decrease of pump pulse. With the high robustness, TABS allows for higher energy injection into the system to further enhance the SNR and measurement accuracy.** One can take at least three measures to further increase the system energy: 1) longer coding length, 2) higher probe power, and 3) higher pre-amplification power. **With the additional energy being injected, achieving the required SNR and target measurement accuracy will become easier.**

All together, benefitting from the transient acoustic wave (wideband and time-weighted Brillouin gain evolution), TABS can reach significantly improved sensing range, spatial resolution, and temporal resolution with comparable accuracy, in comparison to conventional high-spatial-resolution BOTDA.

# **Supplementary Section 10: Analyses of the ETT monitoring**

**
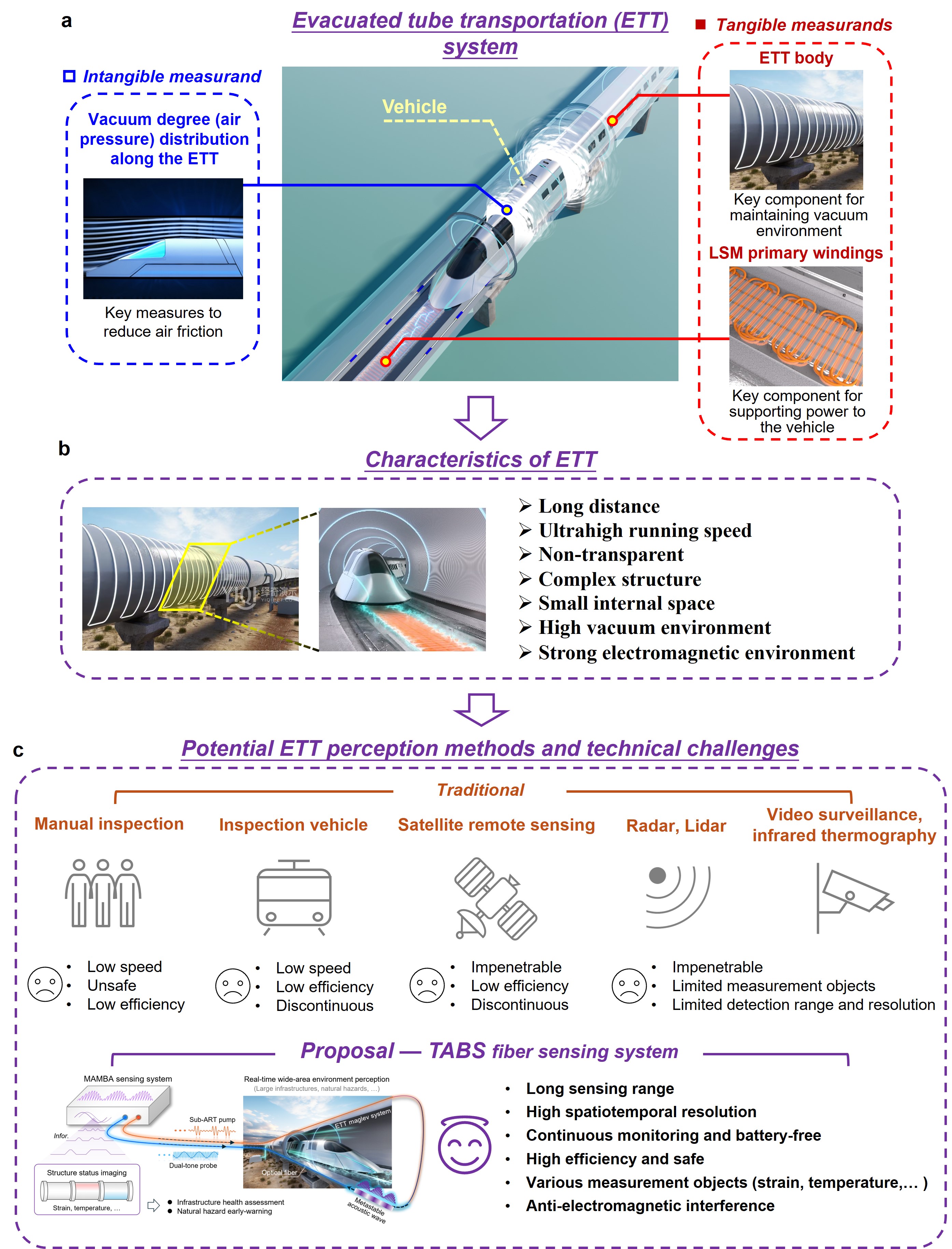
**

**Fig. S16: Evacuated tube transportation (ETT) system. a,** Key components of ETT. LSM: linear synchronous motor. **b,** Characteristics of ETT. **c,** Protentional methods for precepting the ETT system and current technical challenges.

**Evacuated tube transportation (ETT) is a next-generation high-speed train**, which is currently under intensive investigation^47-50^. There have been a lot of prototype systems developed so far, such as Super-Maglev^47, 48^, Hyperloop^49, 50^, Swissmetro, and so forth. The basic idea of ETT is to **1)** utilize magnetic levitation technology to create a certain spacing between the train and guideway to avoid friction, and **2)** adopt the evacuated tube technique to decrease the air density around the train body to reduce the aerodynamic resistance and noise significantly. After overcoming the challenges of friction and air resistance, the train's running speed^47, 48^ is expected to exceed 1000 km·h^-1^, with higher energy efficiency, lower carbon emissions, more convenient transportation, and greater economic benefits, than conventional high-speed railway with 250 km·h^-1^ speed. The key components of ETT are listed and shown in **Fig. S16** The ultra-high running speed can undoubtedly bring much higher transport efficiency, but the emergency response time left to the train control system is extremely short. Any abnormal situation in the ETT system will threaten the train safety and stability. Therefore, **it is essential to** **provide early warning of abnormal situations along the whole evacuated tube system**.

There are many potential techniques for ETT monitoring, including manual inspection, track inspection vehicles, video surveillance, satellite remote sensing, infrared thermography, radar, Lidar, and other electrical sensors^75-80^, as illustrated in **Fig. S16**. Although these techniques have been widely applied in high-speed railway or other scenarios in our daily lives, it may be challenging for them to be applied in the ETT due to the **ETT’s own characteristics**^47-50^.

1. **The evacuated tube extends over a long distance and maintains a high vacuum environment**, it is impractical to employ the traditional manual inspection to finely detect the tube impairments. While the inspection vehicle^75^ has a limited detection speed and has a certain inspection time interval, the detection efficiency is relatively low.
2. **The evacuated tube has non-transparent and complex internal and external structures**, external inspection methods including video surveillance^76^, satellite remote sensing^77, 78^, and infrared thermography^79^ are unable to detect the conditions of internal structures of the ETT system.
3. **The evacuated tube features small internal space and strong electromagnetic environment.** The narrow space inside the tube constrains the detection and radiation angles of the video surveillance, infrared thermography, radar, and Lidar^80^, which makes them hard to achieve long-distance measurements. At the same time, the performance of electrical sensors is challenged by the harsh electromagnetic environment.

Therefore, to effectively monitor the ETT system, a new set of high-performance sensing technologies is highly desirable to **overcome the above difficulties** and realize the high-precision, high-speed, and high-resolution perception of long-range ETT system.

**The ETT is one of the typical applications that needs to measure remote dynamic targets**. In this work, we explore the possibility of the use of TABS sensing system for the state imaging of ETT key components, including **evacuated tube body** and **synchronous linear motor**. The evacuated tube body and synchronous linear motor are chosen in our investigation since they are two key components that relate to air tightness and vehicle traction of the ETT system. Their health condition directly relates to the safety and stability of ETT system.

Apart from the monitoring of the ETT system’s key components above, the **vacuum degree distribution** **(i.e., air pressure distribution)** along the evacuated tube is also an important aspect and has been considered in our investigation. From the technical reports^47-50^, the ETT system will contain a large number of tube flanges, vacuum pumps, exhaust valves, passenger escape doors, and other equipment that exposure to the atmosphere environment. Aging or failure of the equipment or the breakage of the evacuated tube body may cause air leakage and local vacuum degree decrement. Meanwhile, as it is known, aerodynamic drag increases quadratically with vehicle velocity. For the ETT vehicle running at subsonic or supersonic speed (>1000 km·h^-1^ in general), any vacuum degree decrement and non-uniform vacuum distribution will generate additional reactive force and heating to the vehicle^58^, which may give rise to vehicle body tremor or even deformation. Accordingly, the monitoring of vacuum degree distribution is critical to vehicle safety, stability, and comfortability.

# **Supplementary Section 11: State imaging and localizing of ETT system by TABS**


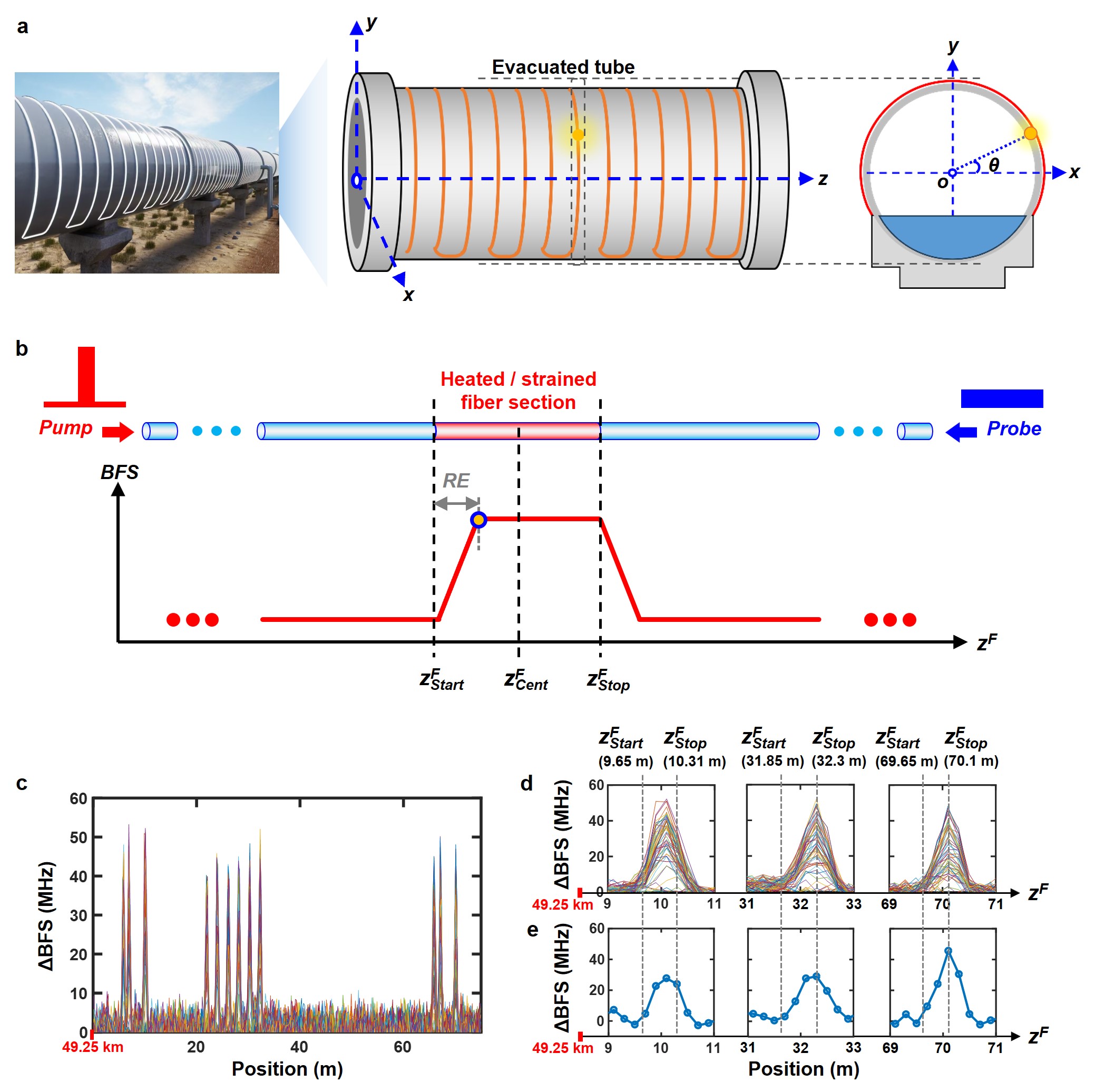


**Fig. S17: Method of evacuated tube three-dimensional coordinate mapping through TABS-measured fiber location. a, b,** Schematic diagrams of (a) three-dimensional and front views of evacuated tube and (b) BFS evolution along the hot spot. RE: rising edge. **c,** Measured BFS variation distribution along the fiber section installed on the evacuated tube. **d,** Determination of hot-spot start and stop locations for pinpointing the positions of anomalous events along the evacuated tube body. **e,** One of the traces in (d).

As depicted in **Fig. S17a**, the sensing cable is installed on the evacuated tube in a serpentine manner. The spacing (Δ*z_0_*) between adjacent rounds of fiber cable is 5 cm. The fiber length per round (*L_per_*) is measured to be 206.9 cm on average. When a structure deformation or temperature rise occurs at any position of the evacuated tube, the fiber cables near this position will respond immediately. The resulting BFS variations and locations will be detected by TABS sensing system. Assuming the TABS-measured fiber location at the center of the structure deformation or temperature-rise section is$z_{Cen}^{F}$, the three-dimensional coordinate (*x*, *y*, *x*) of the position can be deduced by:

$x=r\cdot\cos\left[\angle\theta+\left(N_{round}\%2\right)\pi\right]$ (S14)

$y=r\cdot\sin\left(\angle\theta\right)$ (S15)

$z=\left(N_{round}+1\right)*\Delta z_{0}$ (S16)

where *r* is the radius of the evacuated tube. The *r* is 50 cm in the experiment.$\angle\theta$is the angle corresponding to the (*x*, *y*) coordinate at each location *z*, as illustrated in **Fig. S17b**. *N_round_* indicates the round number of the optical fiber cable that senses the temperature or strain variation, as described below:

$\angle\theta=\frac{2\left(z_{Cen}^{F}\%L_{per}\right)-L_{per}+\pi r}{2r}$ (S17)

$N_{round}=\left\lfloor z_{Cen}^{F}/L_{per}\right\rfloor$ (S18)

$z_{Cen}^{F}=\left(z_{Stop}^{F}-z_{Start}^{F}\right)/2$ (S19)

where $z_{Start}^{F}$ and $z_{Stop}^{F}$ are fiber locations where the BFS starts and stops to vary, as illustrated in **Fig. S17b**. The $z_{Start}^{F}$ can be determined by subtracting the location of the first peak BFS variation (orange-blue dot in **Fig. S17b**) by the spatial resolution. While $z_{Stop}^{F}$ can be determined by finding the first point where the BFS starts to fall. The accuracy of the event positioning is directly proportional to the spatial resolution.


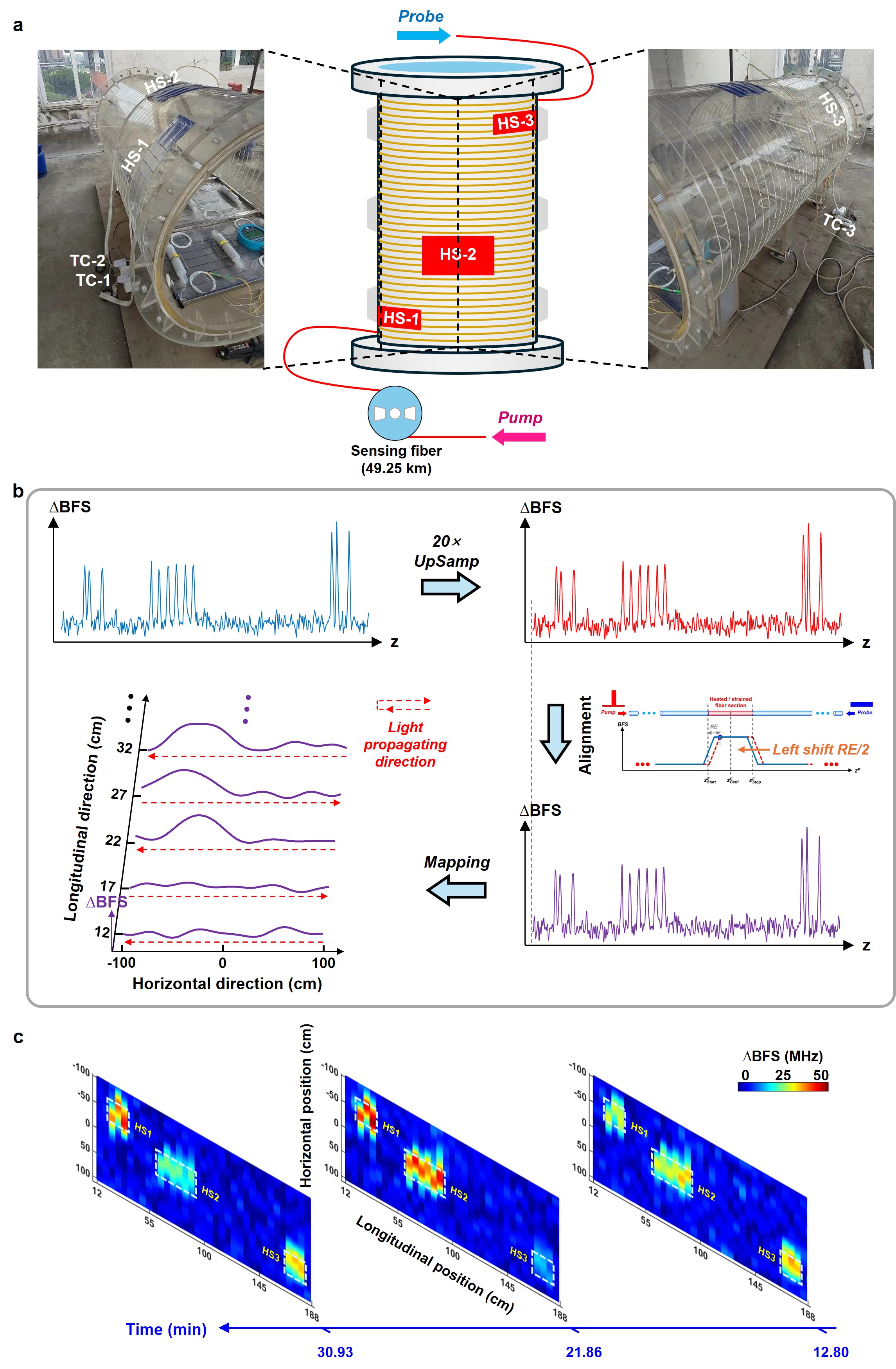


**Fig. S18**: **Analysis** **of evacuated tube state imaging process.** **a,** Schematic diagram and real evacuated tube in the test. HS: heat source, TC: temperature controller. **b,** Signal processing flow of evacuated tube state imaging. **c,** Imaged evacuated tube state at different times. Dashed white box indicates the true locations of the HSs.

In the experiment, the sensing fiber deployed on the evacuated tube is connected to the far-end of the 49.25 km long sensing fiber to mimic long-distance evacuated tube measurement. In this case, the Brillouin signal intensity reaching the evacuated tube is minimal. Three heat sources with sizes of (45×13.5) cm^2^, (49×28) cm^2^, and (45×13.5) cm^2^ are deployed on the evacuated tube at the center locations of (-42.34, 26.59, 32) cm, (0, 50, 79.5) cm, and (42.86, 25.74, 177) cm, respectively. **Figure S17c** shows the TABS measured-BFS variations. **Figure S17d** illustrates the details at the center fiber locations of (49.25 km + 9.98 m), (49.25 km + 32.075 m), and (49.25 km + 69.875 m) of **Fig. S17c**. **Figure S17e** shows the detail of **Fig. S17d** in the 24^th^ minute. The ADC sample rate is 500 MSa·s^-1^, corresponding to a 20-cm interval between adjacent sampling points. From **Figs. S17d to S17e**, it can be found that, by seeking the locations of the first peak point and first falling point, the $z_{Start}^{F}$ and $z_{Stop}^{F}$ can be determined.

**Figure S18a** shows the schematic diagram and digital photograph of evacuated tube in the experiment. **Figure S18b** shows the evacuated tube state imaging process, including the following steps:

**➀ Up-sampling:** the input 1-D sensing signal is up-sampled by N times by using linear interpolation.

**➁ Alignment:** signal alignment operation is performed as shown in **Fig. S18b**. As discussed above, due to the integrator process of SBS, the center of the measured hot spot is delayed by a time equals to the raising edge of sensing signal. Thus, the signal alignment operation is to move the signal forward by half of a raising edge duration, which is achieved by removing the first few elements (with the number equals to half the raising edge duration divided by the sample rate after the up-sampling) of the time-domain sequence.

**➂ Matrixing:** the 1-D sequence is converted to a 2-D matrix to map the TABS-measured location to the unfolded tube location, as illustrated in **Fig. S18b**. It is worth noting that the unfolded tube location (2-D matrix) is used here for visual clarity, and the 3-D coordinate of the tube can be achieved by **Eqs. S17 to S19** above.

After the signal processing above, the state imaging is completed. **Figure S18c** shows the imaged evacuated tube state at different times (the areas in the black dotted boxes illustrate the true locations of the pre-set heat sources). It can be observed that the three heat sources with time-varied temperatures are precisely imaged.

Similar to the evacuated tube body, the **state imaging and localization of the 2-D** **primary windings of long primary linear synchronous motor (LSM)** can be achieved by the methods shown in **Fig. S18**. **Figure S19a** shows the schematic diagram of the primary windings of synchronous linear motor (LSM). The LSM can be seen as a common rotary synchronous motor that has been unfolded (expanded) into a linear structure. A rotary synchronous motor typically consists of a circular stator and a rotor located at the center. In contrast, an LSM unfolds this circular structure, turning the stator into a straight plate (i.e., the primary windings of LSM on the ground. The cross-section of insulated wires in the primary windings is round or rectangular), while the rotor moves forward or backward along the linear stator to produce linear motion (i.e., the secondary permanent magnets (induction plates) on the vehicle).

**The temperature rise may be a challenge for the primary winding of LSM^57^** since 1) the primary windings require high power to drive the giant vehicle. High heat inevitably occurs in the primary windings during its working due to resistance; 2) In contrast to the common atmospheric environment, the primary windings cooling is severely inhibited due to tiny air convection and air conduction in the vacuum environment of ETT; 3) The thick and sealed evacuated tube restricts the diffusion of thermal radiation. To secure the LSM from the damage caused by abnormal temperature rise, it is essential to perform temperature-rise monitoring of the primary windings.

The wire of LSM is installed in a serpentine manner in general. Naturally, the sensing fiber can be embedded into the cable to form the sensing matrix for imaging the state of LSM. Compared with non-contact sensing methods, such as infrared thermography, this kind of embedded sensing approach can fundamentally achieve much higher precisions and much lower latency for the LSM temperature-rise imaging.

Accordingly, **the sensing fiber cable is installed on the mimic LSM in a serpentine layout.** **Figure S19b** shows the mimic LSM and sensing fiber installation. **Figure 19c** shows the mimic


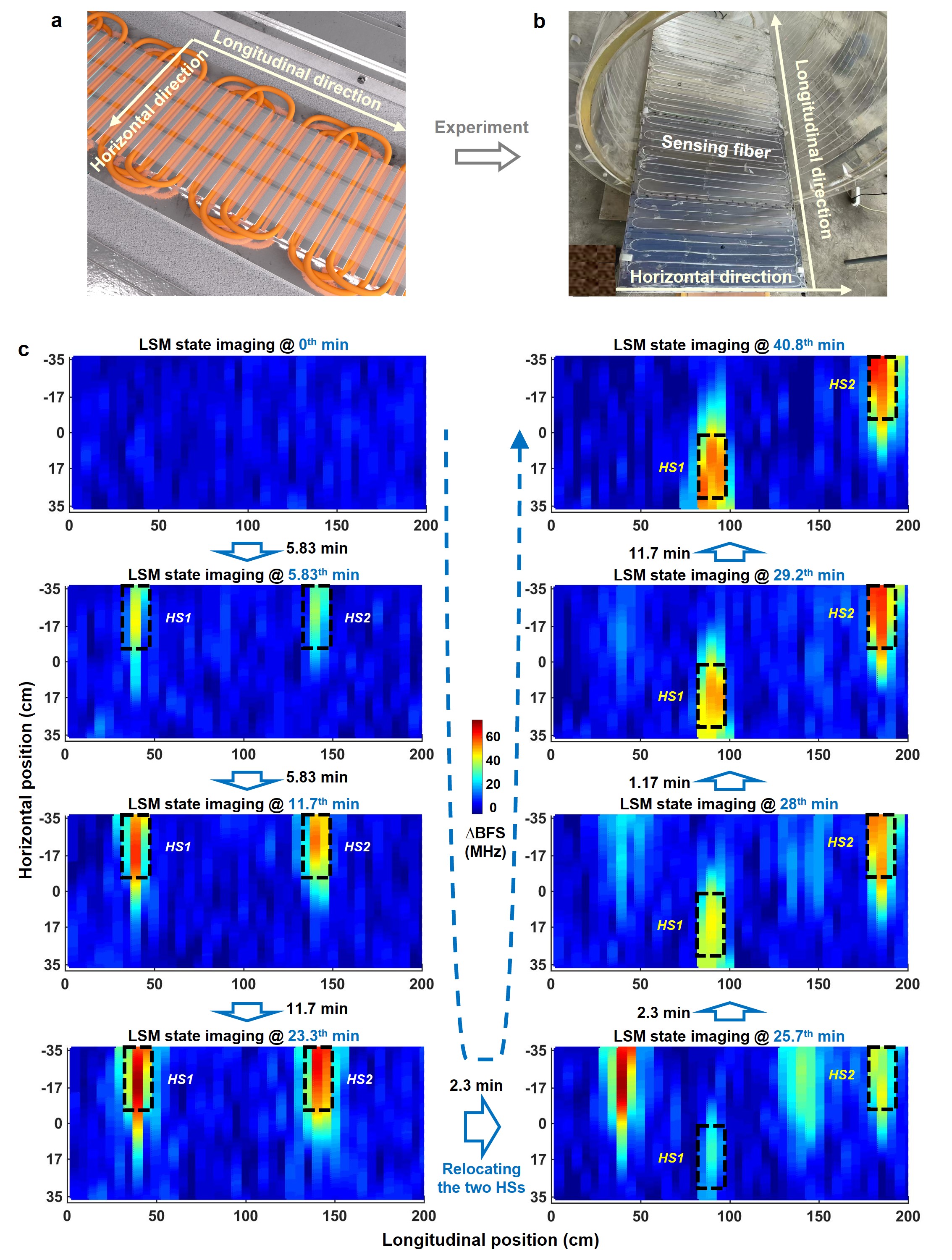


**Fig. S19**: **Imaged LSM** **thermal diffusion at different times. a,** Schematic diagram of primary windings of LSM on the ground. **b,** Test configuration of mimic primary windings temperature-rise imaging. **c,** TABS-imaged temperature-rise variation process of the mimic LSM at different times. In the 0^th^ to 25^th^ min, the two HSs are placed at the center coordinates of (15, 39.9) cm and (15, 140.5) cm (x, z). At this time, the temperature of the two HSs gradually rises from 15℃ to 80℃, to test the sensor’s response to continuous temperature rise. Then, in the 25^th^ to 51.3^th^ min, the two heated HSs (80℃) are relocated to (53.4, 87.7) cm and (15, 183.2) cm positions to test the sensor’s response to sudden temperature rise. Dashed black box indicates the true locations of the HSs.

synchronous linear motor state under different times. It can be observed that the temperature rise area is gradually enlarged from 0^th^ to 20^th^ minute and from 25^th^ to 35^th^ minute, due to the thermal diffusion characteristics of metal materials of the mimic synchronous linear motor. Besides, at 22^th^ minute, the 2 HSs are relocated. The synchronous linear motor temperature evolutions at the past and present two locations can be clearly observed from 22^th^ to 25^th^ minute. From the above results, TABS is capable of recording remote dynamic events thanks to its high spatiotemporal resolution sensing capability.

# **Supplementary Section 12: Working principle of VSA**

To perform the distributed vacuum-degree measurement along the evacuated tube, we propose a vacuum-sensitive-array-assisted TABS system. **Figure 5b** in the main text shows the working principle. The VSA is formed by linearly cascaded vacuum sensitive units (VSUs) which convert the vacuum degree variations to the fiber strain variations. The details about VSU structure and fabrication process are detailly introduced in **Supplementary Section 13**. When the VSUs are deployed along the evacuated tube, the inner and outer pressure difference leads to large longitudinal strain on the VSU’s innermost sensing cable, which results in large BFS variation at the VSU location. Due to the narrowband energy transportation characteristic of the SBS, the acoustic wave can be stimulated only at the VSU locations when the pump-probe frequency offset is aligned to the local BFS at the VSU location, as illustrated in **Fig. 5b** in the main text. As a result, the pump-probe energy transformation will occur only at the VSU location, and the resulting Brillouin gain only relates to the VSU despite the pump pulse width being larger than the VSU length (Theoretical analyses about acoustic wave stimulation manner under the VSU is listed in **Supplementary Section 14**). Even though the pulse width is tens of ns (but shorter than the spacing between adjacent VSUs), the VSU with centimeter-level size can still be measured without sensing information crosstalk. This kind of sub-spatial-resolution measurement capability is unique to the SBS effect and delightful to miniaturize the VSU size.

In the distributed vacuum degree measurement, the VSUs are discretely installed along the evacuated tube in series with a certain spacing. The key to measuring the vacuum degree distribution by the **quasi-distributed VSUs** is that different from the temperature or strain, **the air is diffusive**. Once the air permeation occurs in a certain tube position, the air permeated into the tube will diffuse to adjacent areas rapidly and then be sensed by the nearest VSUs. Accordingly, densely distributing the VSUs is not strictly needed.

In the pump-probe SBS process, the acoustic waves at different VSUs’ locations are stimulated sequentially, which brings periodical energy transportations (i.e., Brillouin gain peaks (BGPs), and BGSs) that only relate to the VSUs, as illustrated in **Fig. 5b** in the main text. The BGP features the same shape as the acoustic wave evolution and a width approximate to the pump pulse width. When the pump pulse width is tens of ns, the BGP features tens of ns width and tens of MHz bandwidth. In this situation, the low-pass filtering can be used to improve the SNR or equivalently reduce the averaging time, meanwhile a low sample rate can be employed to reduce the data-acquisition time. These two factors work together to accelerate the one-shot measurement. It should be noted that the pulse width should be shorter than the VSU spacing to avoid the sensing information crosstalk between adjacent VSUs. Finally, by extracting the BFSs from the BGSs corresponding to different VSUs, the vacuum degree distribution along the evacuated tube can be determined. Moreover, due to the discretely distributed VSUs, the SBS occurs discretely. As a result, total SBS interaction length and total pump-probe energy transformation are reduced significantly, which suppresses the detrimental effects of the SBS process^17, 18, 35-38 68^. The alleviation of the detrimental effects enables high-power pump and probe waves to be used to substantially improve the SNR and measurement accuracy.

Altogether, the VSA-assisted Brillouin sensing system is capable of offering high vacuum sensitivity and high measurement accuracy to reach high-resolution distributed vacuum sensing. Different from the strain and temperature sensing that needs continuously distributed sensing, the vacuum degree measurement only needs quasi-distributed VSUs. As a result, the acoustic wave is discretely stimulated which alleviates the detrimental effects parasitized in the SBS process. Accordingly, the acoustic wave can be stimulated to a steady state to maximize the gain while be free from the impacts of detrimental effects.

# **Supplementary Section 13: Fabrication and sensitivity of VSU**


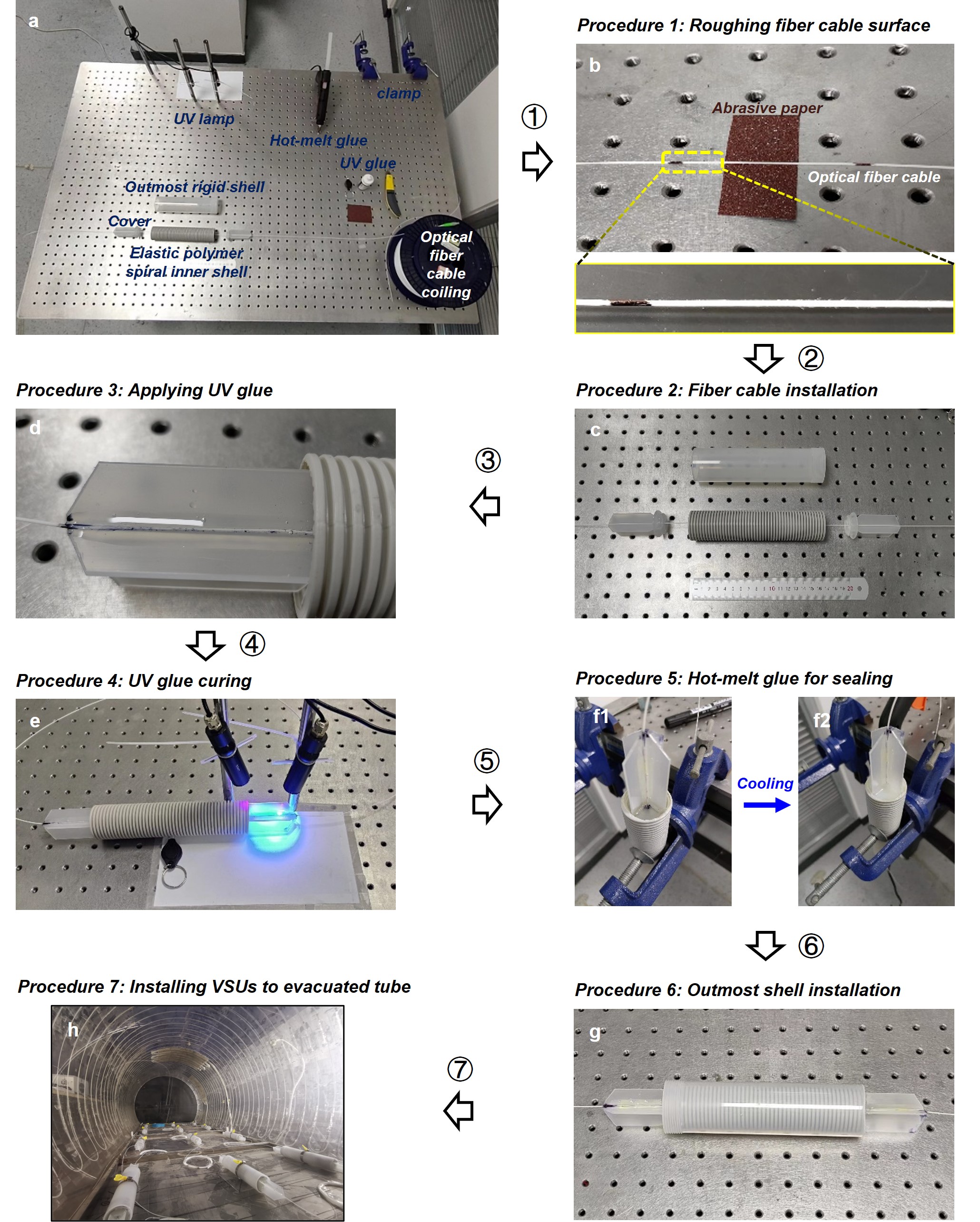


**Fig. S20: Fabrication process of vacuum sensitivity unit. a,** Materials preparation. **b,** Roughing fiber cable surface with an abrasive paper. **c,** Installing optical fiber cable into elastic polymer spiral inner shell. **d,** Securing optical fiber cable to the seal lids by UV glue. **e,** Curing the UV glue with UV light. **f1,** Sealing the VSU by hot melt glue. **f2,** Hot melt glue cooling after approximately 10 minutes. **g,** Installing the elastic polymer spiral shell to the outermost rigid shell. **h,** Deploying the VSUs into the evacuated tube.

**Figure S20** shows the vacuum sensitivity unit (VSU) fabrication process. **Figure S20a** shows the key components of VSU and tools for VSU fabrication. The VSU is constituted by an outermost rigid shell, elastic polymer spiral inner shell, seal lid, and innermost fiber cable, as introduced in the Materials and Methods section.

The VSU fabrication process includes the following steps:

**➀ Roughening the fiber cable**: The surface of the original optical fiber cable is too smooth for high-adhesion glue to bond effectively under large force. Therefore, to enhance friction, the surface of the fiber cable must be roughened. In the experiment, two sections of the fiber cable, each 3 cm in length and spaced 15 cm apart, are roughened, as illustrated in **Fig. S20b**. The fiber cable used is a tight-buffered optical fiber cable (TBF SF1011-A-900E) with an external diameter of 900 ± 50 μm, and a strain endurance of 20000 με, supplied by YOFC Co. Ltd, China.

**➁** **Fiber cable installation**: After roughening the surface, the optical fiber cable is sequentially passed through the seal lids and the elastic polymer spiral inner shell. Then, the two seal lids are inserted into the opposite ends of the elastic polymer spiral inner shell, as shown in **Fig. S20c**;

**➂ Applying the UV glue**: The two roughened sections of the optical fiber cable are secured to the seal lids using UV glue. The UV glue used in the experiment is ergo3800 from Ergo Co., Switzerland, with a viscosity of 1200 mPas, as illustrated in **Fig. S20d**;.

**➃ UV glue curing**: The UV glue cures after approximately 10 minutes of exposure to UV light. The light intensity of the UV LED used in the experiment is 7000 mW·cm^-2^. A small pre-stress is applied to the optical fiber cable before curing the opposite end to ensure that the fiber is straightened, as shown in **Fig. S20e**;

**➄** **Hot melt glue for sealing**: The pre-installed VSU is placed vertically using a clamp. Hot melt glue is then applied to one side of the lid. Once the glue on this side has cooled and solidified, it is applied to the other side, as illustrated in **Fig. S20f**;

**➅ Outermost rigid shell installation**: Once the hot melt glue at both ends has cured, the elastic polymer spiral shell is passed through the outermost rigid shell, completing the VSU installation, as shown in **Fig. S20g**;

**➆ Installing the VSUs into the evacuated tube**: The prepared VSUs are installed into the evacuated tube with a 10 m spacing, as illustrated in **Fig. S20h**.

The VSU is the key element to the distributed vacuum degree measurement. To ensure the elasticity, sensitivity, and long-term durability of vacuum degree measurement, the elastic polymer spiral shell with good longitudinal ductility is adopted for sealing and transduction. When the VSU is placed inside the evacuated tube, due to the difference in internal and external air pressure, the internal air molecules will push the sealing lids at both ends to drive the internal sensing cable to strain. The resulting strain can be described as:

$\Delta\varepsilon=\frac{\Delta P\cdot A_{S}^{T}}{E_{S}^{TJ}\cdot A_{S}^{TJ}+E_{S}^{FCJ}\cdot A_{S}^{FCJ}+E_{S}^{F}\cdot A_{S}^{F}}$ (S20)

where ∆*ε* denotes fiber longitudinal strain, Δ*P* is the VSU internal and external air pressure difference, $A_{s}^{T}$is the cross-sectional area of the elastic polymer spiral shell, $E_{s}^{TJ}$,$E_{s}^{FJ}$, and $E_{s}^{F}$ represent Young's modulus of the elastic polymer spiral shell jacket (equals to the difference between the outer and inner areas), optical fiber cable jacket and optical fiber, respectively. $A_{s}^{TJ}$, $A_{s}^{FJ}$, and $A_{s}^{F}$ are cross-sectional areas of the elastic polymer spiral shell, optical fiber cable jacket, and optical fiber, respectively.

From **Eq. S20**, it can be found that the vacuum-to-strain conversion efficiency is directly proportional to the elastic polymer spiral shell’s cross-sectional area ($A_{s}^{T}$) and inversely proportional to Young's modulus ($E_{s}^{TJ}$, $E_{s}^{FJ}$, and $E_{s}^{F}$) and effective cross-sectional area ($A_{s}^{TJ}$, $A_{s}^{FJ}$, and $A_{s}^{F}$) of the elastic polymer spiral shell, optical fiber cable jacket and optical fiber. By choosing the spiral shell with different materials and cross-sectional areas, the vacuum sensitivity and measurement range can be flexibly shifted to meet different application requirements.

The VSU in the experiment features a 0.474 με·Pa^-1^ vacuum-to-strain conversion factor. Meanwhile, the B-DFOS has a strain measurement certainty of 15.8 με (equals to 0.79 MHz×20με·MHz^-1^). As a result, the VSA-assisted sensing system has a pressure resolution of 33 Pa, which is comparable to the resolution of commercial high-sensitivity barometers (The high-precision barometer in this work features a 10 Pa resolution).

# **Supplementary Section 14: Acoustic wave stimulation in the VSA-assisted Brillouin sensing**


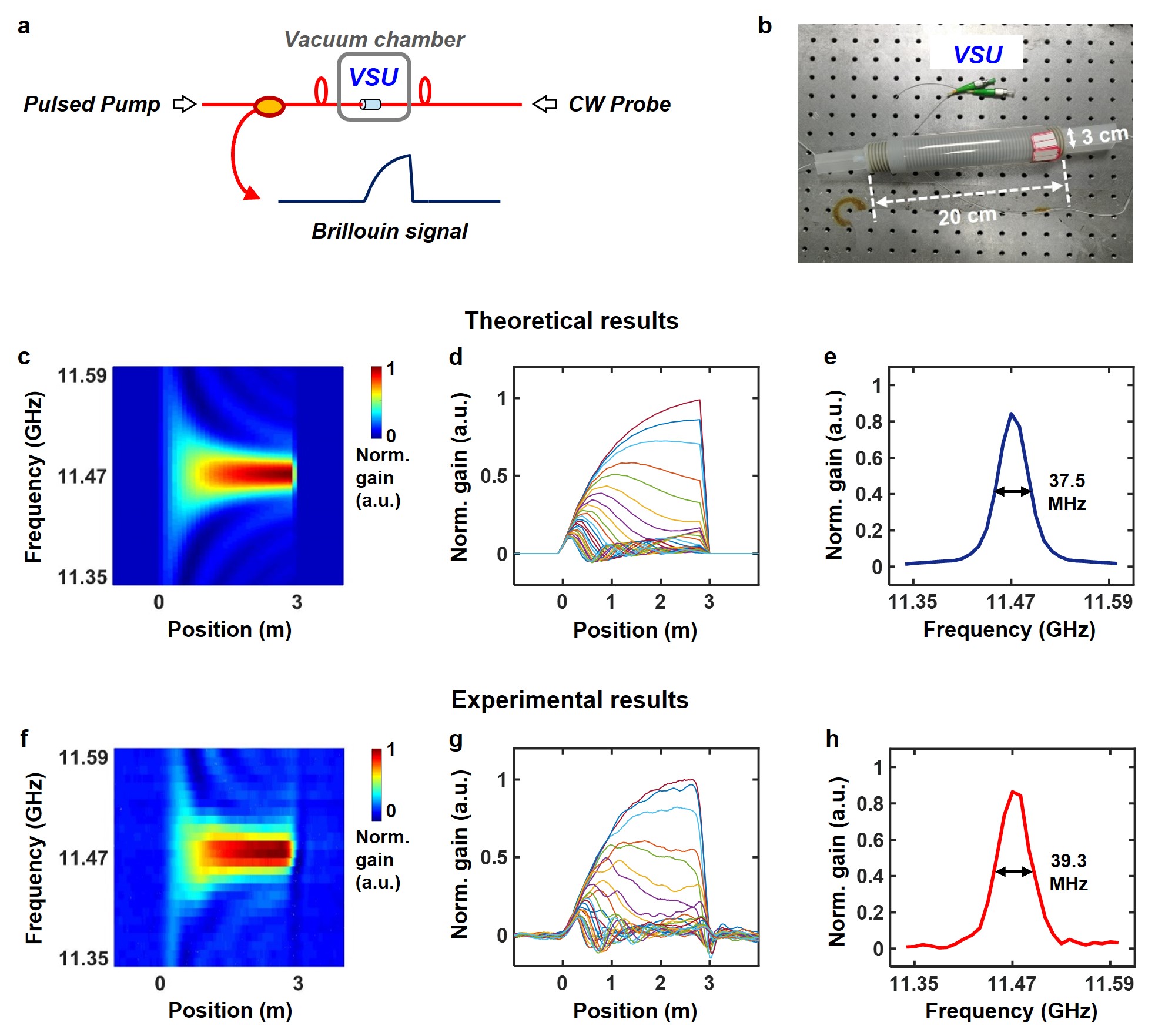


**Fig. S21: Theoretical and experimental analyses of the Brillouin gain evolution along the vacuum sensitivity unit (VSU).** **a,** Test configuration in both simulation and experiment. **b,** Real VSU in the experiment. **c, d, e, f, g, h,** The BGS distributions in (c), (f) top view, (d), (g) side view, and (e), (h) BGS in theory and experiment, respectively.

To investigate the acoustic wave stimulation manner and BGS distribution in the VSA-assisted Brillouin sensing, the numerical simulation and experiment are carried out.

Firstly, the acoustic wave stimulation manner in a single VSU is investigated. The simulation and experimental configurations is depicted in **Fig. S****21a**. The pulse width is 3 m. The VSU under test is shown in **Fig. S21b**. The VSU is 20 cm long and 3 cm in diameter. The lengths of lead-in and lead-out fiber of the VSU are both ~67.2 cm. The VSU is placed in a vacuum chamber. The vacuum degree variation induces fiber strain variation. As a result, the local BFS becomes 11.4721 GHz which is far away from the overall BFS of ~10.82 GHz. The theoretically calculated BGS distribution is shown in **Fig. S21c**. **Figure S21d** is the side view of **Fig. S21c** and shows the time domain traces under different frequencies. **Figure S21e** shows the averaged BGS which is calculated from the BGSs between 1 m and 3 m. Meanwhile, the measured BGS distribution is shown in **Figs. S21f to S21h**. It can be found that the theoretical results match well with the experimental results. As the BFS at the VSU location is far away from the overall BFS, the acoustic wave can be stimulated only at the VSU location even though the length of fiber section in the VSU is much shorter than the pulse width. As a result, **the acoustic wave evolution (from stimulating phase to steady state) is perfectly replicated by the Brillouin gain**, as shown in **Figs S21c to S21d and S21f to S21g**. In both theoretical and experimental results, the width of the Brillouin signal is 3 m which matches well with the pump pulse width. The width of Brillouin signal’s falling edge is 20 cm which is the same as the VSU length. The Brillouin linewidth of the averaged BGS is measured to be 39.3 MHz which matches well with the Brillouin linewidth of 37.5 MHz in the theory.

Furthermore, the BGS distribution in the VSA-assisted Brillouin-distributed air-pressure sensing is investigated. The simulation configuration is shown in **Fig. S22a**. Three VSUs with a length of 15 cm are discretely placed with a spacing of 10 m (the same as that in the experiment). Similarly, the pulse width is 3 m. The preset Brillouin resonant frequency distribution along the fiber in the simulation is shown in **Fig. 22b**. The resonant frequencies at VSU-1 to VSU-3 are 10.95 GHz, 11.15 GHz, and 11.35 GHz, respectively. The resulting BGS distribution along the fiber is theoretically calculated and shown in **Fig. S22c**. The Brillouin gain traces at 10.85 GHz, 11.15 GHz, and 11.35 GHz are shown in **Fig. S22d**. The BGSs at 10 m (z_1_), 20 m (z_2_), and 30 m (z_3_) are shown in **Fig. S22e**. **Figure S22f** shows the zoomed-in view of **Fig. S22e**. It can be observed that the BGS related to each VSU (at, z_1_, z_1_, or z_3_) contains a main peak (relates to the overall BFS of the sensing fiber) and a secondary peak (relates to the BFS of the VSU). The secondary peak is easier to identify as it goes farther away from the main peak (i.e., larger fiber


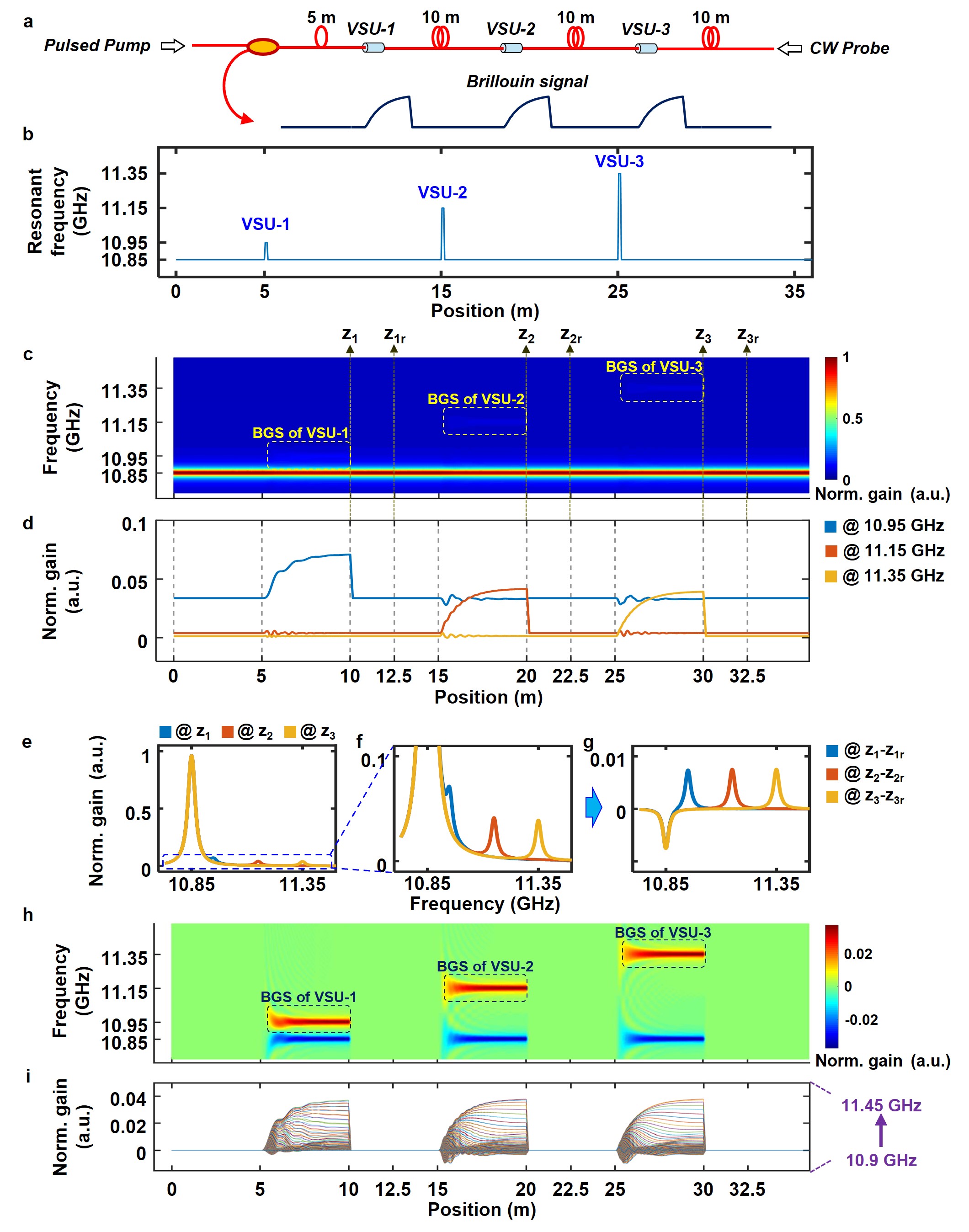


**Fig. S22: Theoretical analysis of the Brillouin gain distribution along the vacuum sensitivity array.** **a,** Configuration in theoretical simulation. **b,** Preset Brillouin resonant frequency distribution along the fiber. **c, d,** Calculated (c) BGS distribution and (d) Brillouin gain traces under different pump-probe frequency offsets. Norm. normalized. **e,** BGSs at z_1_ to z_3_. **f,** Zoomed-in view of (E). **g,** BGS after background elimination operation. **h,** **i,** The (h) Top and (i) sectional view BGS distribution with eliminated background component.

BFS variation induced by the VSU under a higher vacuum degree). While the secondary peak close to the main peak is still affected by a background component from the main peak, which impacts the vacuum degree measurement accuracy.

To eliminate the impact of the background component, one method is to subtract the BGS at the VSU location from the BGS near the VSU. The optical fiber manufactured by modern technology generally has a relatively uniform refractive index distribution, which leads to a relatively uniform BFS distribution along the fiber. Accordingly, the main peaks of the fiber areas close to the VSU location could feature nearly the same BFS as that of the VSU location, which makes it possible to eliminate the impact of background component by subtracting the BGS at the VSU by the BGS near the VSU. **Figures S22g to S22i** show the BGS and BGS distributions after the subtracting operation, the reference points (z_1r_ to z_3r_) of the z_1_ to z_3_ are selected at 12.5 m, 22.5 m, and 32.5 m, respectively, in positions where there are no VSU-related SBS interactions. This background component elimination method is also implemented in the experiment, the result is illustrated in **Figs. 5d and 5e** in the main text. From both simulation and experimental results, the main peak-induced background component is effectively eliminated and the VSUs-related BGSs are quite clean.

The above results demonstrate that the frequency-dependent-gain characteristics of the SBS endow the sensing information of the VSUs to be extracted without crosstalk even though the pulse width (5 m) is much larger than the VSU length (15 cm). This kind of sub-pulse-width spatial resolution is unique to the SBS. It is worth noting that, as the Brillouin signals corresponding to different VSUs are discretely distributed, a low-pass filter (LPF) with a bandwidth of 10 MHz (corresponding to the 10 m spacing between adjacent VSUs) can be adopted to improve SNR without sensing information crosstalk. Meanwhile, as the sensing information within the 50-ns long BGP only relates to the 15-cm long VSU, a sample rate of 10 MSa·s^-1^ (corresponding to a 10 m spatial sample interval) or 20 MSa·s^-1^ (corresponding to a 5 m spatial sample interval) is enough to acquire sensing information belongs to the BGP, which shorten the data acquisition time significantly. In the experiment, a 20-MHz LPF and a 100-MSa·s^-1^ sampling rate are employed, and the resulting Brillouin signal is illustrated in **Fig. 5** in the main text.

# **Supplementary Section 15: Details of the experiment of vacuum degree measurements**

**
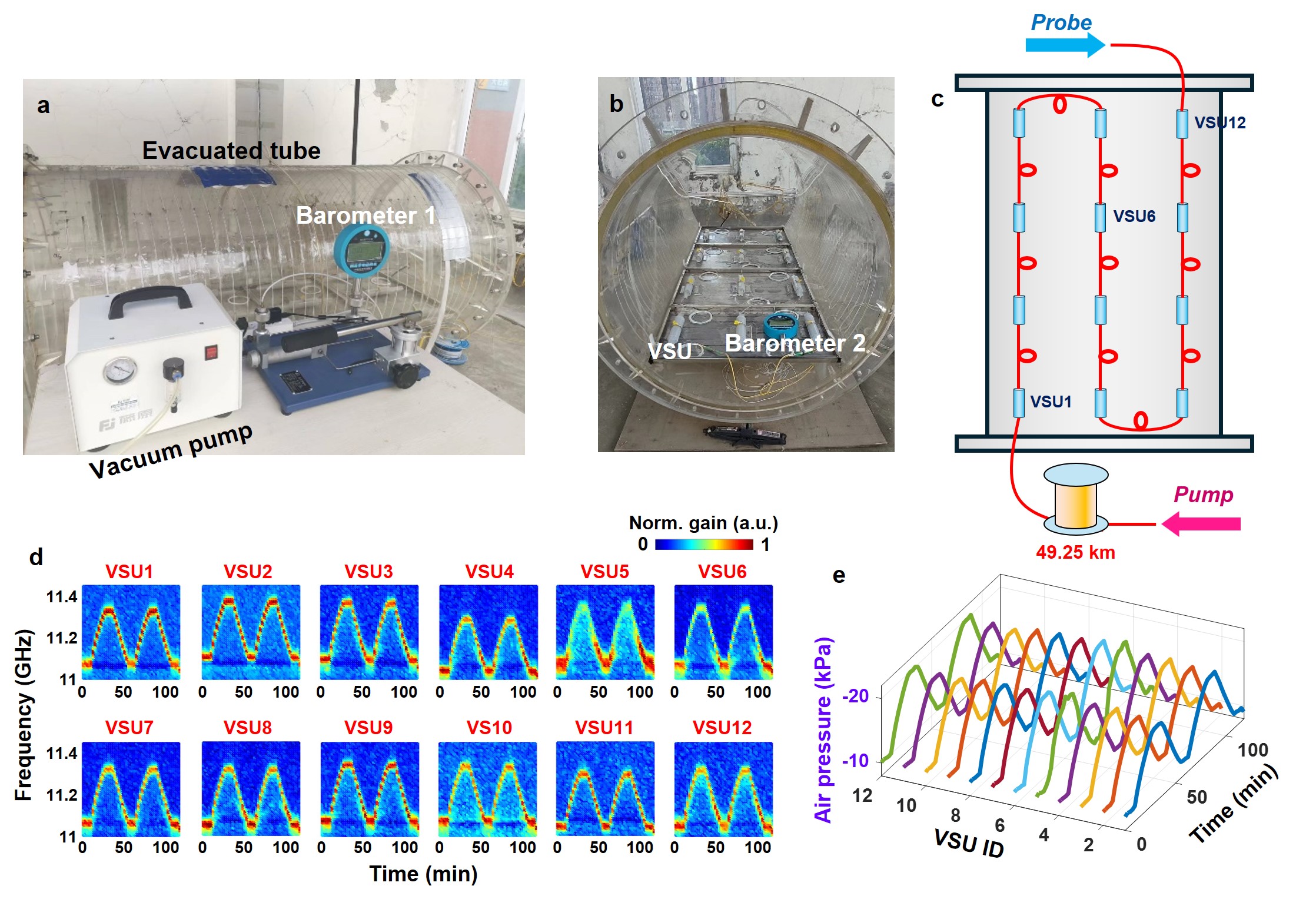
**

**Fig. S23: Details of the vacuum degree measurement in the experiment. a,** Digital photograph of the evacuated tube test system. **b, c,** (b) Digital photograph of the evacuated tube inner structure and (c) schematic diagram of VSA installation. **d, e,** The (d) Measured BGSs and (e) extracted BFS variations of different VSUs at different times.

**Figures S23a and S23b** show the actual field test of the vacuum degree distribution measurement in the experiment. Twelve VSUs with the same spacing of ~10 m are placed at the end of the 50 km long sensing fiber and installed in the evacuated tube, as illustrated in **Fig. S23c**. The VSU in the experiment is 15 cm long and 1 cm in radius. The sensing system configuration is the same as that in the TABS, while the pump pulse width is 50 ns. The bandwidth of LPF, sample rate of OSC, and averaging number are 20 MHz, 100 MSa·s^-1^, and 500, respectively. In the dynamic vacuum degree measurement shown in **Fig. 5i** in the main text, the evacuated tube inner pressure is adjusted by the vacuum pump. The vacuum degree increment and decrement are achieved by turning on and off the vacuum pump, respectively. The measured BGSs and extracted BFSs corresponding to the 12 VSUs are shown in **Figs. S23d and S23e**, respectively, demonstrating that all VSUs feature high repeatability. Notably, the extracted BFSs in **Fig. S23e** are corrected by the 12 VSUs’ vacuum sensitivities shown in **Fig. 5g** in the main text.

# **Supplementary Section 16: Fabrication of VSU with variable vacuum measurement ranges**

**
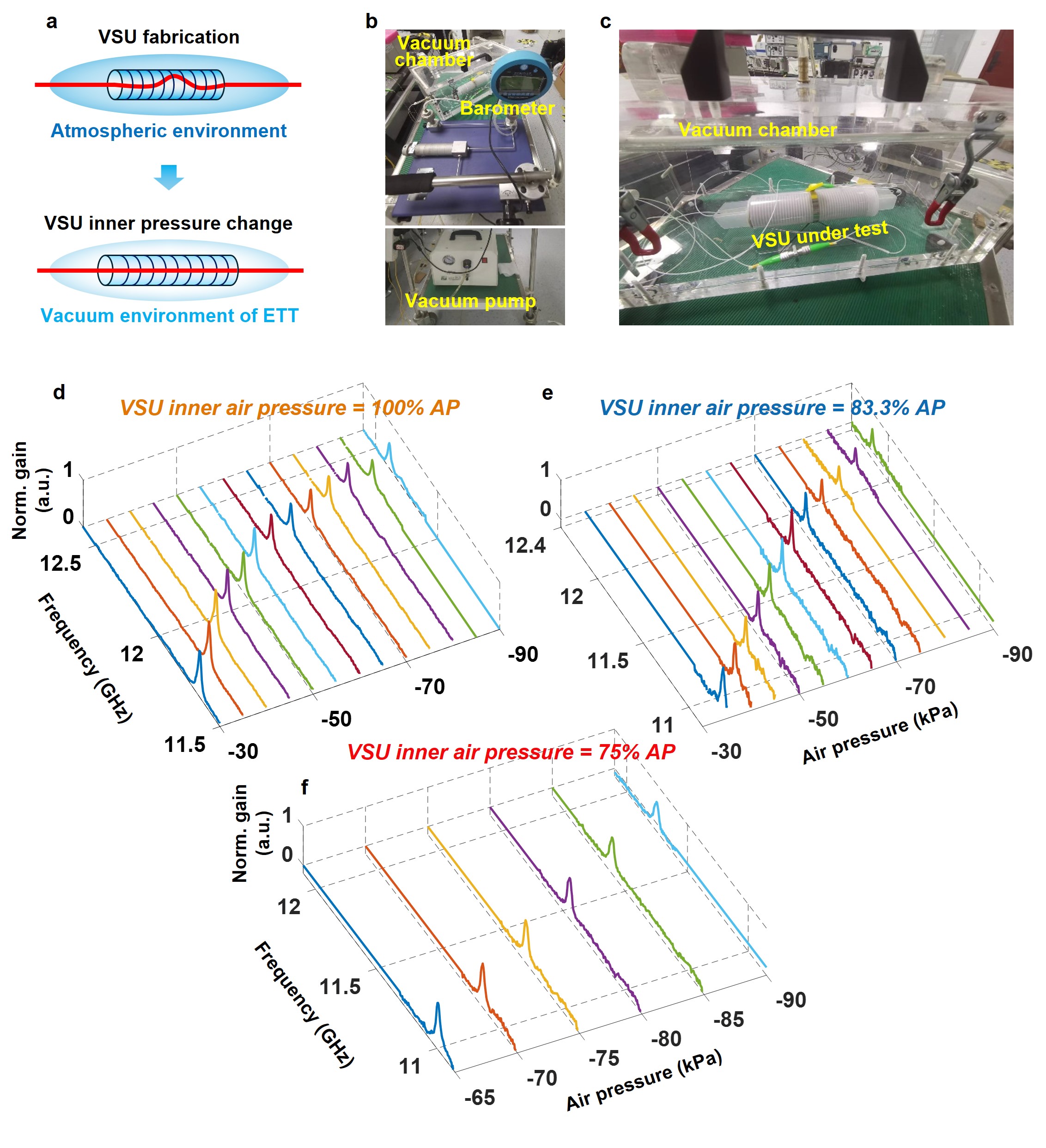
**

**Fig. S24: Details of the VSU fabrication with different vacuum measurement ranges.** **a,** Schematic diagram of the VSU fabrication. **b, c,** Digital photographs of (b) test setup and (c) vacuum chamber in the test. **d, e, f,** Measured BGSs of the VSUs with inner air pressures (d) 100%, (e)83.3%, and (f) 75% of atmospheric pressure (AP).

**Figure S24a** shows the fabrication of VSU with different vacuum measurement ranges. The key to improving the vacuum measurement range while retaining the vacuum sensitivity is to change the VSU inner air pressure. To reach this goal, during the VSU packaging process in the atmospheric environment, the fiber length in the VSU is a bit longer than the VSU length. As a result, when the packaged VSU is installed in the evacuated tube, the VSU will expand and the VSU’s inner pressure decreases accordingly. The fiber starts to be strained until the VSU length equals the inner fiber length. As the vacuum-induced strain variation is only related to the internal and external air pressure difference, the VSU inner pressure decrement will increase the ceiling of vacuum degree measurement while maintaining the vacuum sensitivity. As illustrated in **Fig. 5h** in the main text, by using the 15-cm long VSUs with 15-cm, 18-cm and 20-cm long inner fibers, the VSU inner air pressures are 100%, 83.3% and 75% of atmospheric pressure (AP), which enables the ceilings of vacuum degree measurement to be ~45% (-45 kPa), ~80% (-80 kPa) and 100% (-101 kPa), respectively. Moreover, **Fig. S24b and S24c** show the actual test configuration of vacuum measurement range. The VSU is inserted into a vacuum chamber with variable vacuum degrees. The measured BGSs and extracted BFSs under different vacuum degrees corresponding to the VSUs with 100%, 83.3%, and 75% of AP are shown in **Figs. S24d to S24f** in the supplementary text and **Fig. 5h** in the main text, respectively.

# **References**

1. Stuart-Smith, R. F. et al. Increased outburst flood hazard from Lake Palcacocha due to human-induced glacier retreat. *Nature Geoscience* **14**, 85-90 (2021).
2. Shugar, D. H. et al. A massive rock and ice avalanche caused the 2021 disaster at Chamoli, Indian Himalaya. *Science* **373**, 300-306 (2021).
3. Zhao, J. et al. Forest fire size amplifies postfire land surface warming. *Nature* **633**, 828-834 (2024).
4. Hjort, J. et al. Impacts of permafrost degradation on infrastructure. *Nature Reviews Earth & Environment* **3**, 24-38 (2022).
5. Liu, K. et al. Global transportation infrastructure exposure to the change of precipitation in a warmer world. *Nature Communications* **14**, 2541 (2023).
6. Ferdowsi, A., Zolghadr-Asli, B. & AghaKouchak, A. Dangers of aging water infrastructure. *Science* **386**, 158-158 (2024).
7. Li, J. X. et al. The break of earthquake asperities imaged by distributed acoustic sensing. *Nature* **620**, 800-806 (2023).
8. Glisic, B. Introduction to Strain-Based Structural Health Monitoring of Civil Structures. (Chichester: John Wiley & Sons, 2024), 368.
9. Yang, Z. Y. et al. A vision chip with complementary pathways for open-world sensing. *Nature* **629**, 1027-1033 (2024).
10. Kobyakov, A., Sauer, M. & Chowdhury, D. Stimulated Brillouin scattering in optical fibers. *Advances in Optics and Photonics* **2**, 1-59 (2010).
11. Eggleton, B. J. et al. Brillouin integrated photonics. *Nature Photonics* **13**, 664-677 (2019).
12. Motil, A., Bergman, A. & Tur, M. [INVITED] State of the art of Brillouin fiber-optic distributed sensing. *Optics & Laser Technology* **78**, 81-103 (2016).
13. Hartog, A. H. An Introduction to Distributed Optical Fibre Sensors. (Boca Raton: CRC Press, 2017), 472.
14. Lu, P. et al. Distributed optical fiber sensing: Review and perspective. *Applied Physics Reviews* **6**, 041302 (2019).
15. Soto, M. A. & Thévenaz, L. Modeling and evaluating the performance of Brillouin distributed optical fiber sensors. *Optics Express* **21**, 31347-31366 (2013).
16. Dong, Y. K. et al. 150 km fast BOTDA based on the optical chirp chain probe wave and Brillouin loss scheme. *Optics Letters* **43**, 4679-4682 (2018).
17. Sun, X. Z. et al. Genetic-optimised aperiodic code for distributed optical fibre sensors. *Nature Communications* **11**, 5774 (2020).
18. Zhou, Y. et al. Hybrid aperiodic coding for SNR improvement in a BOTDA fiber sensor. *Optics Express* **29**, 33926-33936 (2021).
19. Sun, X. Z. et al. Ultra-long Brillouin optical time-domain analyzer based on distortion compensating pulse and hybrid lumped–distributed amplification. *APL Photonics* **7**, 126107 (2022).
20. Wang, B. Z. et al. High spatial resolution: an integrative review of its developments on the Brillouin optical time- and correlation-domain analysis. *Measurement Science and Technology* **31**, 052001 (2020).
21. Li, W. H. et al. Differential pulse-width pair BOTDA for high spatial resolution sensing. *Optics Express* **16**, 21616-21625 (2008).
22. Foaleng, S. M. et al. High spatial and spectral resolution long-range sensing using brillouin echoes. *Journal of Lightwave Technology* **28**, 2993-3003 (2010).
23. Soto, M. A. et al. Optimization of a DPP-BOTDA sensor with 25 cm spatial resolution over 60 km standard single-mode fiber using Simplex codes and optical pre-amplification. *Optics Express* **20**, 6860-6869 (2012).
24. Zadok, A. et al. Random-access distributed fiber sensing. *Laser & Photonics Reviews* **6**, L1-L5 (2012).
25. Denisov, A., Soto, M. A. & Thévenaz, L. Going beyond 1000000 resolved points in a Brillouin distributed fiber sensor: theoretical analysis and experimental demonstration. *Light*: *Science & Applications* **5**, e16074 (2016).
26. Zarifi, A. et al. High resolution brillouin sensing of micro-scale structures. *Applied Sciences* **8**, 2572 (2018).
27. Li, Z. L. Hybrid Golay-coded Brillouin optical time-domain analysis based on differential pulses. *Optics Letters* **43**, 4574-4577 (2018).
28. Ge, Z. et al. Enabling variable high spatial resolution retrieval from a long pulse BOTDA sensor. *IEEE Internet of Things Journal* **10**, 1813-1821 (2023).
29. Peng, J. X. et al. High spatial resolution BOTDA based on deconvolution and all phase digital filtering. *IEEE Sensors Journal* **24**, 10024-10030 (2024).
30. Zhou, Y. et al. Long-range high-spatial-resolution distributed Brillouin sensing enabled by correlation-domain encoding. *Optics Letters* **48**, 3143-3146 (2023).
31. Youn, J. H. et al. Brillouin expanded time-domain analysis based on dual optical frequency combs. *Light*: *Science & Applications* **13**, 149 (2024).
32. Peled, Y., Motil, A. & Tur, M. Fast Brillouin optical time domain analysis for dynamic sensing. *Optics Express* **20**, 8584-8591 (2012).
33. Zhou, D. W. et al. Slope-assisted BOTDA based on vector SBS and frequency-agile technique for wide-strain-range dynamic measurements. *Optics Express* **25**, 1889-1902 (2017).
34. Zhou, D. W. et al. Single-shot BOTDA based on an optical chirp chain probe wave for distributed ultrafast measurement. *Light*: *Science & Applications* **7**, 32 (2018).
35. Thévenaz, L., Mafang, S. F. & Lin, J. Effect of pulse depletion in a Brillouin optical time-domain analysis system. *Optics Express* **21**, 14017-14035 (2013).
36. Iribas, H. et al. Non-local effects in Brillouin optical time-domain analysis sensors. *Applied Sciences* **7**, 761 (2017).
37. Yang, Z. S. et al. Design rules for optimizing unipolar coded Brillouin optical time-domain analyzers. *Optics Express* **26**, 16505-16523 (2018).
38. Gao, X. et al. Impact of optical noises on unipolar-coded Brillouin optical time-domain analyzers. *Optics Express* **29**, 22146-22158 (2021).
39. Bao, X. et al. Characterization of the Brillouin-loss spectrum of single-mode fibers by use of very short (<10-ns) pulses. *Optics Letters* **24**, 510-512 (1999).
40. Lecoeuche, V. et al. Transient response in high-resolution Brillouin-based distributed sensing using probe pulses shorter than the acoustic relaxation time. *Optics Letters* **25**, 156-158 (2000).
41. Bao, X. Y. et al. Influence of transient phonon relaxation on the Brillouin loss spectrum of nanosecond pulses. *Optics Letters* **31**, 888-890 (2006).
42. Dong, Y. K., Chen, L. & Bao, X. Y. Truly distributed birefringence measurement of polarization-maintaining fibers based on transient Brillouin grating. *Optics Letters* **35**, 193-195 (2010).
43. Dong, Y. K. et al. 2 cm spatial-resolution and 2 km range Brillouin optical fiber sensor using a transient differential pulse pair. *Applied Optics* **51**, 1229-1235 (2012).
44. Zhou, D. W. et al. Millimeter-level recognition capability of BOTDA based on a transient pump pulse and algorithm enhancement. *Optics Letters* **46**, 3440-3443 (2021).
45. Tsuji, K., Noda, H. & Onodera, N. Sweep-free brillouin optical time domain analysis using two individual laser sources. *Optical Review* **19**, 381-387 (2012).
46. Oppenheim, A. V. & Willsky, A. S. Signals & Systems. (Pearson Educación, 1997).
47. Deng, Z. G. et al. A high-temperature superconducting maglev-evacuated tube transport (HTS Maglev-ETT) test system. *IEEE Transactions on Applied Superconductivity* **27**, 3602008 (2017).
48. Deng, Z. G. et al. A high-speed running test platform for high-temperature superconducting maglev. *IEEE Transactions on Applied Superconductivity* **32**, 3600905 (2022).
49. Decker, K. et al. Conceptual feasibility study of the hyperloop vehicle for next-generation transport. Proceedings of the 55th AIAA Aerospace Sciences Meeting. Grapevine, TX, USA: American Inst. of Aeronautics and Astronautics, 2017, 9-13.
50. Abdelrahman, A. S., Sayeed, J. & Youssef, M. Z. Hyperloop transportation system: analysis, design, control, and implementation. *IEEE Transactions on Industrial Electronics* **65**, 7427-7436 (2018).
51. Long, Z. H. et al. One-wire reconfigurable and damage-tolerant sensor matrix inspired by the auditory tonotopy. *Science Advances* **9**, eadi6633 (2023).
52. Inaudi, D. & Glisic, B. Development of distributed strain and temperature sensing cables. Proceedings of the SPIE 5855, 17th International Conference on Optical Fibre Sensors. Bruges, Belgium: SPIE, 2005, 222.
53. Ashby, M. F., Shercliff, H. & Cebon, D. Materials: Engineering, Science, Processing and Design. (Amsterdam: Butterworth-Heinemann, 2007).
54. Deng, Z. G. et al. A high-temperature superconducting maglev ring test line developed in Chengdu, China. *IEEE Transactions on Applied Superconductivity* **26**, 3602408 (2016).
55. Jin, J. X. et al. Applied superconductivity and electromagnetic devices - principles and current exploration highlights. *IEEE Transactions on Applied Superconductivity* **31**, 7000529 (2021).
56. Zhou, X. C. et al. Vertical dynamic response analysis of HTS maglev vehicle excited by a designed coreless-typed PMLSM. *IEEE Transactions on Transportation Electrification* **9**, 3421-3433 (2023).
57. Pei, Z. L. et al. Temperature field calculation and water-cooling structure design of coreless permanent magnet synchronous linear motor. *IEEE Transactions on Industrial Electronics* **68**, 1065-1076 (2021).
58. Sui, Y. et al. Impact of vacuum degree on the aerodynamics of a high-speed train capsule running in a tube. *International Journal of Heat and Fluid Flow* **88**, 108752 (2021).
59. Keshmiri, H. et al. Brillouin light scattering anisotropy microscopy for imaging the viscoelastic anisotropy in living cells. *Nature Photonics* **18**, 276-285 (2024).
60. Zarifi, A. et al. Highly localized distributed Brillouin scattering response in a photonic integrated circuit. *APL Photonics* **3**, 036101 (2018).
61. Yan, L. S., Yu, Q. & Willner, A. E. Uniformly distributed states of polarization on the Poincare Sphere using an improved polarization scrambling scheme. *Optics Communications* **249**, 43-50 (2005).
62. Yao, L. et al. A novel scheme for achieving quasi-uniform rate polarization scrambling at 752 krad/s. *Optics Express* **20**, 1691-1699 (2012).

**Refs. in Supplementary Information:**

1. Zhang, C. K., Yang, Y. H. & Li, A. Q. Application of Levenberg–Marquardt algorithm in the Brillouin spectrum fitting. Proceedings of the SPIE 7129, Seventh International Symposium on Instrumentation and Control Technology, 2008, Beijing, China: SPIE, 2008, 443.
2. Urricelqui, J., Sagues, M. & Loayssa, A. Brillouin optical time-domain analysis sensor assisted by Brillouin distributed amplification of pump pulses. *Optics Express* **23**, 30448 (2015).
3. Soto, M. A., Bolognini, G. & Pasquale, F. D. Optimization of long-range BOTDA sensors with high resolution using first-order bi-directional Raman amplification. *Optics Express* **19**, 4444–4457 (2011).
4. Farahani M. A. et al. Reduction in the Number of Averages Required in BOTDA Sensors Using Wavelet Denoising Techniques. *Journal of Lightwave Technology* **30**, 1134–1142 (2012).
5. Zaslawski, S., Yang, Z. S. & Thevenaz, L. On the 2D Post-Processing of Brillouin Optical Time-Domain Analysis. *Journal of Lightwave Technology* **38**, 3723–3736 (2020).
6. Liu, C. et al. Overcoming EDFA slow transient effect in a Golay-coded BOTDA sensor by a distributed depletion mapping method. *Optics Express* **29**, 27340–27349 (2021).
7. Zhou, D. W., Dong, Y. K. & Yao, J. P. Truly Distributed and Ultra-Fast Microwave Photonic Fiber-Optic Sensor. *Journal of Lightwave Technology* **38**, 4150–4159 (2020).
8. Zhao, C. et al. BOTDA using channel estimation with direct-detection optical OFDM technique. *Opt. Express* **25**, 12698 (2017).
9. Bernini, R., Minardo, A. & Zeni, L. Dynamic strain measurement in optical fibers by stimulated Brillouin scattering. *Optics Letters* **34**, 2613–2615 (2009).
10. Ba, D. X. et al. Distributed measurement of dynamic strain based on multi-slope assisted fast BOTDA. *Optics Express* **24**, 9781–9793 (2016).
11. Wang, S. et al. Study on the signal-to-noise ratio of Brillouin optical-time domain analyzers. *Optics Express* **28**, 19864–19876 (2020).
12. Lopez-Gil, A. et al. Simple Method for the Elimination of Polarization Noise in BOTDA Using Balanced Detection and Orthogonal Probe Sidebands. *Journal of Lightwave Technology* **33**, 2605–2610 (2015).
13. Li, Y. et al. Rail Component Detection, Optimization, and Assessment for Automatic Rail Track Inspection. *IEEE Transactions on Intelligent Transportation Systems* **15**, 760–770 (2014).
14. Wang, X. G. Intelligent multi-camera video surveillance: A review. *Pattern recognition letters* **34**, 3–19 (2013).
15. Moreira, A. et al. A tutorial on synthetic aperture radar. *IEEE Geoscience and remote sensing magazine* **1**, 6–43 (2013).
16. Burke, M. et al. Using satellite imagery to understand and promote sustainable development. *Science* **371**, eabe8628 (2021).
17. Alfredo Osornio-Rios, R., Antonino-Daviu, J. A. & de Jesus Romero-Troncoso, R. Recent Industrial Applications of Infrared Thermography: A Review. *IEEE transactions on industrial informatics* **15**, 615–625 (2019).
18. Behroozpour, B. et al. Lidar System Architectures and Circuits. *IEEE Communications Magazine* **55**, 135–142 (2017).
